# The Microtubule Regulator NEK7 Coordinates the Wiring of Cortical Parvalbumin Interneurons

**DOI:** 10.1016/j.celrep.2018.06.115

**Published:** 2018-07-31

**Authors:** Antonio Jesús Hinojosa, Rubén Deogracias, Beatriz Rico

**Affiliations:** 1 Centre for Developmental Neurobiology, Institute of Psychiatry, Psychology and Neuroscience, King’s College London, London SE1 1UL, UK; 2 MRC Centre for Neurodevelopmental Disorders, King’s College London, London SE1 1UL, UK; 3 Instituto de Neurociencias, Consejo Superior de Investigaciones Científicas & Universidad Miguel Hernández, Sant Joan d’Alacant 03550, Spain

**Keywords:** parvalbumin interneurons, axon development, synapse formation, microtubules, diversity

## Abstract

Functional networks in the mammalian cerebral cortex rely on the interaction between glutamatergic pyramidal cells and GABAergic interneurons. Both neuronal populations exhibit an extraordinary divergence in morphology and targeting areas, which ultimately dictate their precise function in cortical circuits. How these prominent morphological differences arise during development is not well understood. Here, we conducted a high-throughput screen for genes differentially expressed by pyramidal cells and interneurons during cortical wiring. We found that NEK7, a kinase involved in microtubule polymerization, is mostly expressed in parvalbumin (PV+) interneurons at the time when they establish their connectivity. Functional experiments revealed that NEK7-deficient PV+ interneurons show altered microtubule dynamics, axon growth cone steering and reduced axon length, arbor complexity, and total number of synaptic contacts formed with pyramidal cells. Altogether, our results reveal a molecular mechanism by which the microtubule-associated kinase NEK7 regulates the wiring of PV+ interneurons.

## Highlights

• *Nek7* is specifically expressed by GABAergic interneurons during cortical wiring
• Loss of *Nek7 in vitro* alters microtubule dynamics, axon steering, and morphology
• Loss of *Nek7 in vivo* impairs the morphology and synaptic outputs of PV interneurons

## Graphical Abstract

**Figure:**
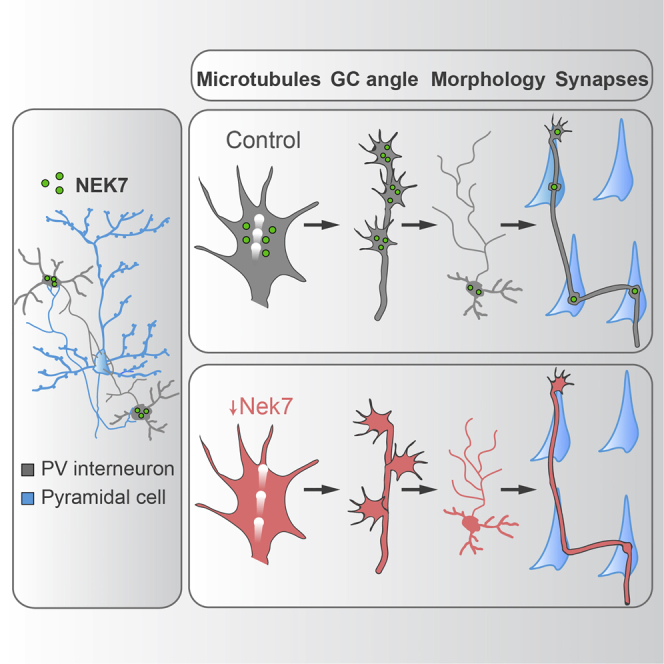

## Teaser

Mature cortical circuits emerge during development by the integration of two morphologically distinguished neuronal populations, pyramidal cells and interneurons. Hinojosa et al. reveal a molecular program by which a microtubule-associated kinase specifically controls the shape and wiring of cortical interneurons.

## Introduction

Identifying the mechanisms by which neurons are precisely wired in specific circuits is critical to elucidate how the extraordinary complexity of brain function emerges. During development, wiring takes place through an ordered sequence of steps. The classical view of neural circuitry assembly proposes that axons first grow toward their target area, arborize through collateral extensions that are subsequently remodeled, and finally form synapses with the corresponding cellular targets (Kolodkin and Tessier-Lavigne, 2011, Shen and Scheiffele, 2010). These events are triggered by local extracellular cues, which instruct active crosstalk with different components of the cytoskeleton (Conde and Cáceres, 2009, Hoogenraad and Bradke, 2009, Kalil and Dent, 2014, Navarro and Rico, 2014).

There are two main classes of neurons in the mammalian cerebral cortex, glutamatergic pyramidal cells, which are excitatory projection neurons, and GABAergic interneurons, which form inhibitory local connections (Custo Greig et al., 2013, DeFelipe et al., 2002, Fishell and Rudy, 2011). The wide diversity of pyramidal cells and interneurons is defined by a unique set of neurochemical, morphological, connectivity, and firing features that are built by specific molecular programs. Among these features, one striking difference between pyramidal cells and interneurons is the morphology of their axons and dendrites. Pyramidal cell dendrites expand toward different cortical layers in a stereotyped fashion, and their axons typically target cells in other layers, as well as other cortical and subcortical areas. On the contrary, interneuron dendrites and axons very often remain in relatively close proximity to the cell soma. In addition, whereas the axons of pyramidal cells are straight, interneuron axons are profusely branched and follow convoluted paths that often correlate with the position of their postsynaptic targets (Dumitriu et al., 2007, Huang et al., 2007, Stepanyants et al., 2004, Tremblay et al., 2016). The molecular mechanisms regulating axon development and synapse formation in pyramidal cells have been studied in detail (de Wit and Ghosh, 2016, Conde and Cáceres, 2009, Kalil and Dent, 2014, Kolodkin and Tessier-Lavigne, 2011, McAllister, 2007, Navarro and Rico, 2014), but our understanding of these mechanisms in interneurons is very rudimentary. A large body of evidence suggests that small variations in the development of GABAergic interneurons may lead to neurodevelopmental disorders (Marín, 2012). Therefore, the identification of molecules that coordinate the development of these cells not only represents a major scientific advance but also has important biomedical implications.

Previous studies have identified genes that are differentially expressed among various populations of cortical neurons (Cembrowski et al., 2016, Sugino et al., 2006, Zeisel et al., 2015). However, how these unique molecular profiles emerge during development is not well understood. Here, we took a transcriptome approach to identify genes that are differentially expressed by GABAergic interneurons and pyramidal cells during the wiring of cortical circuits. We identify the never in mitosis A (NIMA)-related kinase *Nek7* (Morris, 1976, O’Connell et al., 2003) as one of the most enriched transcripts in interneurons during this critical period in cortical development. Targeted downregulation of *Nek7* disrupts microtubule and growth cone dynamics, alters axonal arbor morphology, and causes a reduction in synaptic contacts made by parvalbumin (PV+) interneurons. Our study identifies NEK7 as an essential regulator of a molecular program that regulates microtubule dynamics and axon development in PV+ interneurons.

## Results

### Identification of Differentially Expressed Genes during the Wiring of GABAergic Interneurons

**Figure 1:**
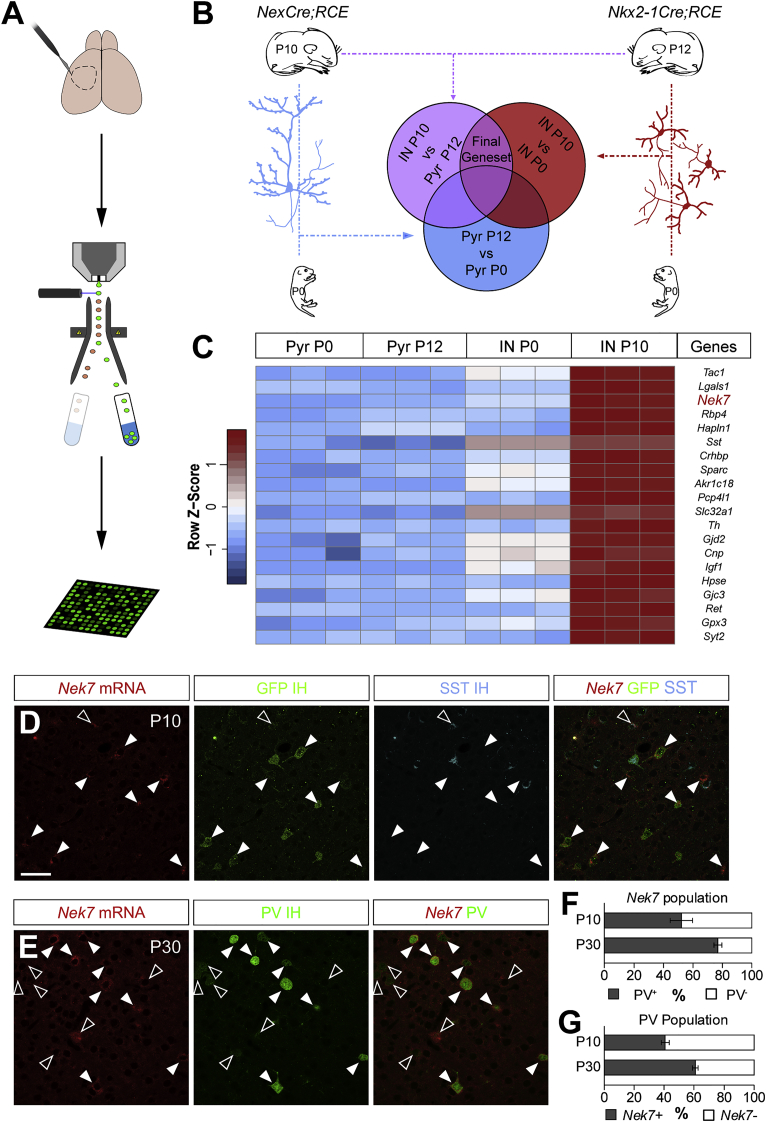
*Nek7* Transcripts Are Upregulated during GABAergic Interneuron Wiring and Expressed Mostly in PV+ Cells (A) Schematic of genetic screening. Cortices from different GFP+ reporter lines were dissected, and cells were dissociated and FACS sorted and their RNA hybridized to microarrays. (B) Bioinformatic comparisons of neuronal populations. (C) Heatmap showing the expression levels of the 20 first ranked genes. *Nek7* is highlighted in red. (D) Confocal images showing *in situ* hybridization for *Nek7* (red) and immunohistochemistry for GFP (green) and SST+ (cyan) in the somatosensory cortex of P10 *Lhx6Cre;RCE* mice. GFP+ and SST+ are somatostatin cells, GFP+ and SST− are putative PV+ interneurons. (E) Confocal images showing *in situ* hybridization for *Nek7* (red) and immunohistochemistry for PV+ cells (green) in the somatosensory cortex of P30 wild-type mice. In (D) and (E), filled arrowheads denote colocalization, and open arrowheads denote no colocalization. (F) Percentage of PV+ (gray) among all *Nek7*-expressing cortical cells at P10 and P30. (G) Percentage of *Nek7*-positive cells among all PV-expressing neurons at P10 and P30. IN, interneurons (red); Pyr, pyramidal cells (blue). Data are represented as mean ± SEM. Scale bar represents 50 μm. See also Figures S1–S4.

Pyramidal cells and interneurons are remarkably different in morphology and synaptic targeting with distinct contributions to the cortical network. To understand how this heterogeneity emerges, we searched for differentially expressed genes between these two neuronal populations during cortical wiring. We selected the temporal window when cortical neurons develop axons and synapses to establish mature circuitries (Ben-Ari, 2002, De Felipe et al., 1997, Larsen and Callaway, 2006) (Figure S1). We used fluorescence-activated cell sorting (FACS) to isolate pyramidal cells and interneurons using mice reporting these populations with GFP at two different stages and subsequently carried out transcriptome analyses (Figure 1A). We performed an unsupervised principal-component analysis demonstrating that each replicate clusters with the corresponding conditions (Figure S2A). In addition, we found no outliers when comparing the overall distribution of signal intensities between microarrays (Figure S2B). To assess the purity of the isolated populations, we searched for genes that are known to be enriched in each of these cell types and upregulated between the different postnatal ages (Figure S2C; Table S1). We then carried out further bioinformatic analyses to search for genes differentially expressed in cortical interneurons compared with pyramidal cells and with higher expression at P10 than at P0 (Figure 1B). We identified a gene set of 133 transcripts with these characteristics (Table S1). The Gene Ontology (GO) database revealed that the most abundant categories included axon- and synapse-related structures, synaptic neurotransmission pathways, and genes encoding for extracellular matrix proteins (Figures S2D and S2E).

We next ranked the 133 differential expressed genes according to two criteria: the specificity ratio, defined by the normalized mean expression within the population of interneurons at P10 compared with the expression across other populations (Kryuchkova-Mostacci and Robinson-Rechavi, 2017), and the transcript expression levels in interneurons at P10 (Figure 1C). On the basis of these criteria, one of the first candidate genes was the serine/threonine kinase *Nek7*, which encodes a protein involved in microtubule dynamics (Cohen et al., 2013) (Figure 1C). qPCR experiments confirmed these findings (Figures S3A–S3D). Moreover, we found that half of *Nek7*-expressing cells are putative PV+ cells at P10 (52.4 ± 7.6%; Figures 1D and 1F) and that *Nek7* was expressed in a subpopulation of these cells (40.6 ± 2.8%; Figures 1D and 1G). We confirmed that the expression of *Nek7* was predominantly in PV+ interneurons at P30 (PV+, 77.0 ± 2.7%; Figures 1E and 1F; SST+, 10.2 ± 0.5%; Figures S3E–S3G) and that a large number of PV+ cells express *Nek7* (60.9 ± 2.0%; Figures 1E and 1G). Our observations are consistent with those of other studies showing that the majority of PV+ interneurons express *Nek7* transcripts (Nakajima et al., 2014, Tasic et al., 2016). Within the PV population, we found that the percentage of NEK7+ PV+ cells was significantly higher in layer VI than in other layers (Figures S4A and S4B). We did not find any correlation with the soma size or with the levels of PV, consistent with the previously reported expression of the kinase in all PV clusters (Figures S4C–S4E; Tasic et al., 2016). These results reveal that *Nek7* is a gene highly enriched in PV+ interneurons and upregulated during postnatal development, which suggests that it might play a role in the morphological differentiation and network integration of PV+ cells.

### *Nek7* Knockdown Accelerates Microtubule Dynamics and Alters Axon Steering

**Figure 2:**
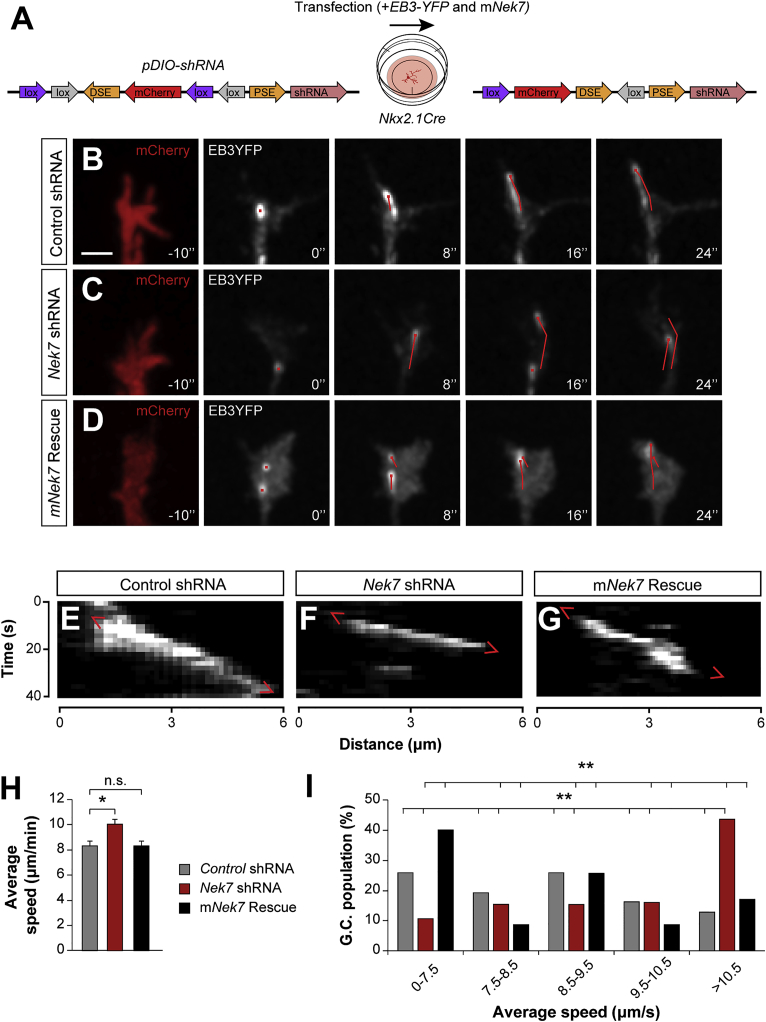
*Nek7* Knockdown Accelerates Microtubule Growth (A) Diagram of the Cre-dependent constructs expressing *mCherry* and shRNA. The plasmids were co-transfected with *EB3-YFP*, and m*Nek7* for the rescue, in primary cortical cultures from *Nkx2-1Cre* mice at 4 DIV, and axons were recorded at 7 DIV. (B–D) Confocal Z projection frames from control shRNA (B), *Nek7* shRNA (C), and rescue with m*Nek7* (D) *Nkx2-1Cre* growth cones expressing mCherry (red, before the time-lapse) and EB3-YFP (gray, time-lapse). The path of EB3-YFP comets is tracked with a red line. Scale bar represents 2 μm. (E–G) Kymographs of EB3 comets in control (E), *Nek7* shRNA (F), and rescue (G) growth cones showing their existence time as a function of distance. (H and I) Average speed (H) and speed distribution of EB3 comets (I) comparing control (n = 31 growth cones), *Nek7*-depleted cells (n = 35 growth cones), and m*Nek7* rescue cells (n = 35 growth cones) from three independent cultures. All growth cones with EB3 comets were quantified. One-way ANOVA (H) and χ^2^ test (I). ^∗^p < 0.05 and ^∗∗^p < 0.01. Data are represented as mean ± SEM (H) or total cell percentage (I). See also Figures S5–S7 and Videos S1, S2, and S3.

NEK7 has been shown to mediate microtubule-dependent processes in both dividing and non-dividing cells (Fry et al., 2012, Kim et al., 2007, Yissachar et al., 2006), probably through the regulation of microtubule dynamics (Cohen et al., 2013). Because microtubule dynamics are essential for axon and dendrite development in neurons (Conde and Cáceres, 2009, Dent et al., 2011, Gordon-Weeks, 2004, Kalil and Dent, 2014), we hypothesized that NEK7 could also regulate microtubule dynamics during the maturation of interneurons. Indeed, NEK7 was enriched in the central domain of interneuron growth cones, a subcellular compartment often populated by highly dynamic microtubules (tyrosinated microtubules) (Dent and Kalil, 2001) (Figure S5). To test this hypothesis, we explored the dynamic behavior of microtubules as reported by the plus-end-binding protein 3 (EB3) using *in vitro* time-lapse imaging (Stepanova et al., 2003). Similarly to *in vivo*, *Nek7* expression was also increasing during axonal development *in vitro* (Figure S3D). To downregulate the expression of *Nek7*, we engineered a Cre-dependent conditional vector expressing a short hairpin RNA (shRNA) against *Nek7* along with the fluorescent marker mCherry as a reporter of recombination (Figure 2A). We assessed the ability of *Nek7* shRNA to decrease *Nek7* levels *in vitro* in HEK cells expressing exogenous full-length *Nek7* (Figures S6A and S6B). To visualize local microtubule polymerization, we co-transfected *Nkx2-1Cre* primary cortical cultures at 7 days *in vitro* (DIV) with Cre-dependent plasmids for *Nek7* or a control shRNA and a plasmid encoding *EB3-YFP* (Figure 2A). Although *Nkx2-1Cre* does not exclusively drive recombination in PV+ cells (Maroof et al., 2010), it allows the recombination of the conditional constructs when the axons are still highly dynamic. We observed that downregulation of *Nek7* in interneurons increases the average speed of EB3-YFP comets compared with controls (Figures 2B–2H and Videos S1, S2, and S3). As expected, differences were observed only in a subpopulation of *Nkx2-1* growth cones, presumably in putative PV+ interneurons that normally express *Nek7* (Figure 2I). We confirmed that the abnormal microtubule behavior was specifically caused by Nek7 knockdown, because we rescued the average speed of EB3-YFP comets with dual infections of *Nek7* shRNA with an shRNA-resistant full-length NEK7 (m*Nek7*; Figures 2D, 2G, and 2H).

Video S1. Microtubule Dynamics in a Growth Cone Expressing Control shRNA, Related to Figure 2 The path of EB3-YFP comets is tracked with a red line.

Video S2. Microtubule Dynamics in a Growth Cone Expressing Nek7 shRNA, Related to Figure 2 The path of EB3-YFP comets is tracked with a red line.

**Figure 3:**
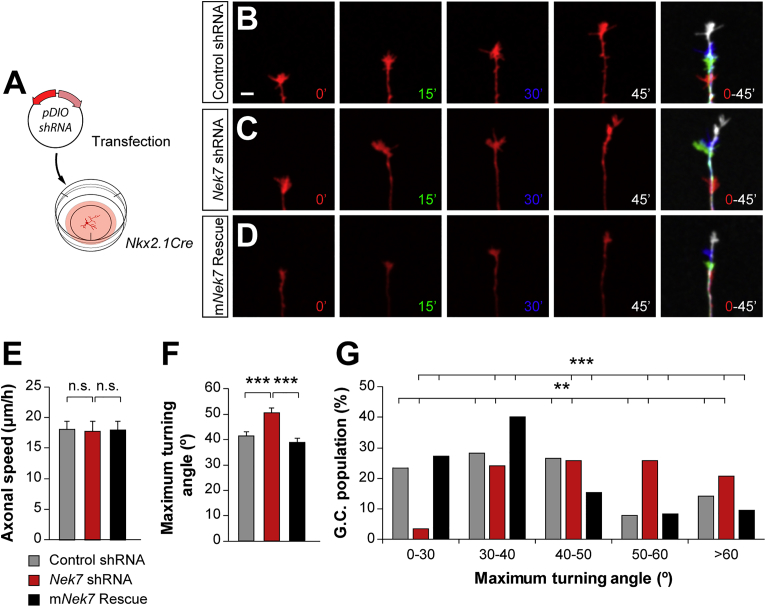
*Nek7* Depletion Impairs Axonal Growth Cone Dynamics *In Vitro* (A) Schematic of experimental design with the Cre-dependent plasmids transfected in *Nkx2-1Cre* primary cortical cultures at 4 DIV and axons recorded at 7 DIV. (B–D) Confocal Z projection frames from axons expressing control (B), *Nek7* shRNA (C), and m*Nek7* rescue (D). The last frame in the sequence shows superimposed images of the frames t = 0 min (red), t = 15 min (green), t = 30 min (blue), and t = 45 min (white). Scale bar represents 5 μm. (E–G) Axonal speed (E), average of growth cone maximum turning angle (F), and its distribution (G) from cells expressing control shRNA (n = 64 growth cones), *Nek7* shRNA (n = 59 growth cones), and m*Nek7* (n = 85 growth cones) from three or four independent cultures. All growth cones increasing in length were quantified. Kruskal-Wallis test, pairwise comparisons (E and F), and χ^2^ test (G). Data are represented as mean ± SEM (E and F) or total cell percentage (G). ^∗∗^p < 0.01 and ^∗∗∗^p < 0.001; n.s., not significant. See also Figures S6 and S7 and Videos S4, S5, and S6.

Video S3. Microtubule Dynamics in a Growth Cone Expressing mNek7, Related to Figure 3 The path of EB3-YFP comets is tracked with a red line.

To examine whether the deficits in microtubule growth led to alterations in axon development, we explored the dynamic behavior of axons *in vitro* (Figure 3; Videos S4, S5, and S6). Although axon growth speed was similar between control and *Nek7* shRNA-expressing neurons, we observed that *Nek7*-depleted growth cones have increased turning angles compared with controls, which suggested a meandering behavior (Figures 3B–3F). This abnormal turning angle was also rescued by expressing m*Nek7* in interneurons (Figures 3D–3F). As before, differences in growth cone turning were observed only in a subpopulation of *Nkx2-1* neurons, most likely the *Nek7*-expressing PV+ interneurons (Figure 3G). Together, these results demonstrate that NEK7 regulates microtubule dynamics and axon steering in interneurons.

Video S4. Growth Cone Dynamics in an Axon Expressing Control shRNA, Related to Figure 3 The angle of the mCherry-labeled axon is tracked with a red line.

Video S5. Growth Cone Dynamics in an Axon Expressing Nek7 shRNA, Related to Figure 3 The angle of the mCherry-labeled axon is tracked with a red line.

Video S6. Growth Cone Dynamics in an Axon Expressing mNek7, Related to Figure 3 The angle of the mCherry-labeled axon is tracked with a red line.

### NEK7 Function Is Required for the Morphological Development of Interneurons

**Figure 4:**
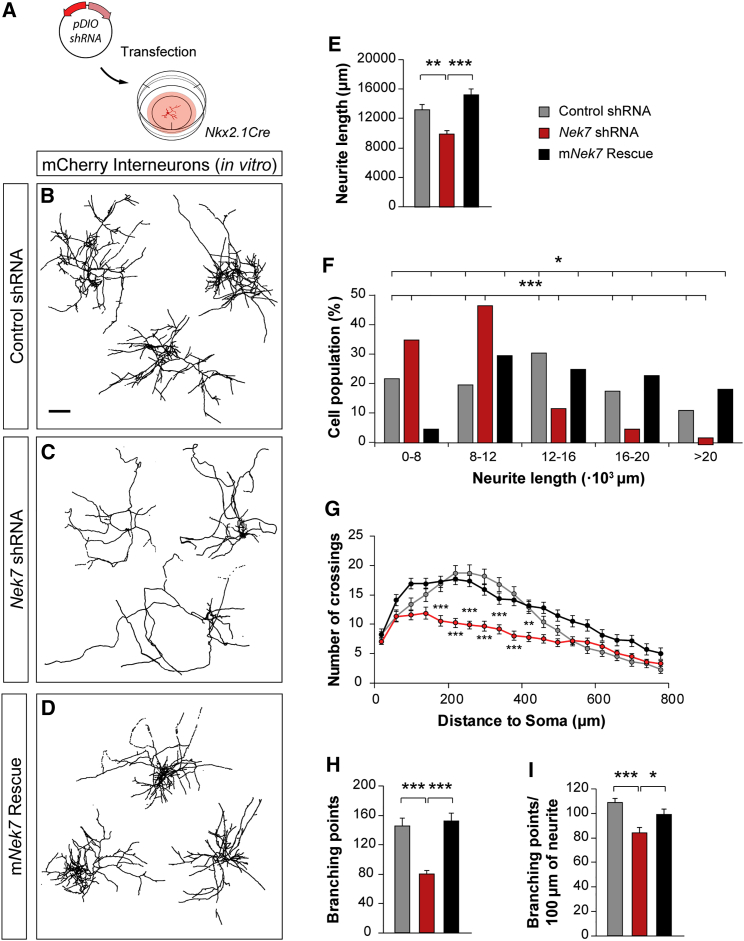
Loss of NEK7 Alters Interneuron Morphology *In Vitro* (A) Schematic of experimental design. *pDIO-shRNA* was transfected in *Nkx2-1Cre* primary cortical cultures. (B–D) Confocal Z projections of *Nkx2-1Cre* interneurons from cortical cultures transfected with control shRNA (B), *Nek7* shRNA (C), and m*Nek7* for rescue (D) expressing mCherry. The cells were automatically reconstructed and masked in black at 12 DIV. Scale bars represent 200 μm. (E–I) Average of total neurite length (E) and its distribution (F), Sholl analysis (G), total branching points (H), and branching points per unit length (I) of control shRNA (n = 47), *Nek7* shRNA (n = 45), and rescue (n = 46) transfected neurons from three or four independent cultures. All imaged cells were quantified. Kruskal-Wallis test, pairwise comparisons (E, H, and I), χ^2^ test (F), and two-way ANOVA with Bonferroni correction (G). ^∗^p < 0.05, ^∗∗^p < 0.01, and ^∗∗∗^p < 0.001. Data are represented as mean ± SEM. See also Figures S6 and S7.

Our previous results suggest that microtubule dynamics and axonal behavior in interneurons seem to depend on NEK7 function. Because interneuron axonal arbors are highly branched and convoluted (Dumitriu et al., 2007, Jiang et al., 2015, Tremblay et al., 2016), defects in axon dynamics may affect normal axonal morphology in these cells. To test whether NEK7 is required for interneuron axonal arborization, we transfected primary cortical cultures obtained from *Nkx2-1Cre* mice with control or *Nek7* shRNA expression vectors and compared the morphology of axonal arbors in both conditions (Figure 4A). We found that the average neurite length was significantly decreased in interneurons with *Nek7* knockdown compared with controls (Figures 4B–4E). As for other parameters, the reduction in neurite length was restricted to a fraction of the cells, presumably PV+ interneurons (Figure 4F). We found that axon complexity and the number of branches were also decreased in interneurons expressing *Nek7* shRNA compared with controls (Figures 4B, 4C, and 4G–4I). Consistently, the total neurite length, complexity, and axonal branches were rescued by m*Nek7* expression in interneurons (Figures 4D–4I). Our results demonstrate that NEK7 regulates different aspects of axon development in interneurons.

**Figure 5:**
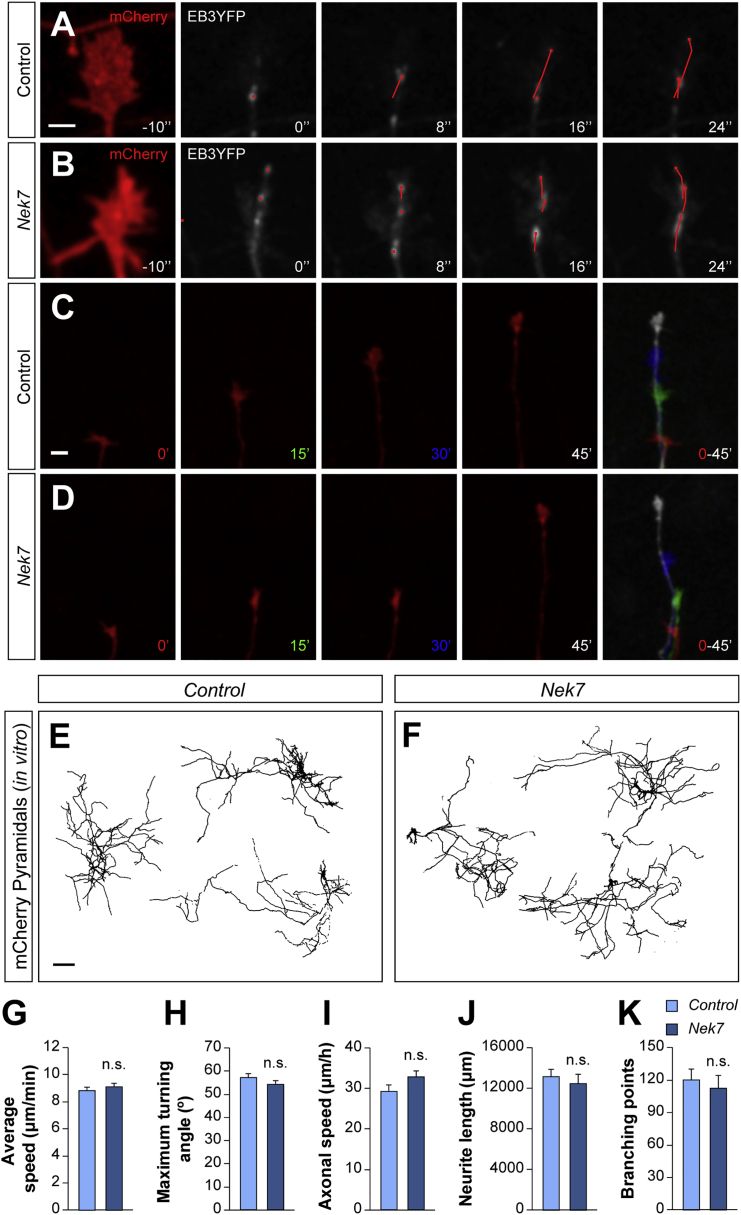
*Nek7* Overexpression in Pyramids Does Not Alter Their Axonal Development (A and B) Confocal Z projection frames from *NexCre* growth cones expressing mCherry (red, before the time-lapse), EB3-YFP (gray, time-lapse), and m*Nek7* in the *Nek7* condition. Control (A) and m*Nek7* (B). The path of EB3-YFP comets is tracked with a red line. (C and D) Confocal Z projection frames from *NexCre* growth cones. Control (C) and m*NEK7* (D). The last frame in the sequence shows superimposed images of the frames t = 0 min (red), t = 15 min (green), t = 30 min (blue), and t = 45 min (white). (E and F) Confocal Z projections of *NexCre* interneurons expressing mCherry. Control (E) and m*Nek7* (F). The cells were automatically reconstructed and masked in black at 8 DIV. (G) Average speed of EB3 comets comparing control pyramids (A; n = 53 growth cones) and *Nek7*-expressing pyramids (B; n = 54 growth cones) from three independent cultures. (H and I) Average of growth cone maximum turning angle (H) and axonal speed (I) from control pyramids (C; n = 73 growth cones) and *Nek7*-expressing pyramids (D; n = 74 growth cones) from three independent cultures. (J and K) Neurite length (J) and branching points (K) comparing control pyramids (E; n = 44 cells) and *Nek7*-expressing pyramids (F; n = 38 cells) transfected neurons from three independent cultures. One-way ANOVA (G and I) and Mann-Whitney test (H, J, and K). n.s., not significant. Data are represented as mean ± SEM. Scale bars represent 2 μm (A and B), 5 μm (C and D), and 200 μm (E and F).

Given that pyramidal cells do not expressed NEK7 (Figures 1C, S3A, and S3B), we wondered whether NEK7 was sufficient to modify microtubule dynamics and axonal development if expressed ectopically in pyramidal cells. Specific overexpression of *Nek7* in pyramids showed similar microtubule dynamics, axonal behavior, and morphology as control pyramidal cells (Figure 5). Altogether, our findings reveal that NEK7 is part of an interneuron-specific molecular program orchestrating axon development.

### Kinase-Dependent and Kinase-Independent Functions of NEK7 in Axon Development

NEK7 is a serine/threonine kinase with a highly conserved catalytic domain (Haq et al., 2015, O’Connell et al., 2003). To examine whether the catalytic activity of NEK7 was required for microtubule dynamics and axon behavior, we engineered a Cre-dependent *Nek7* kinase-dead mutant (*Nek7* KD) that was transfected in primary cultures as described above. We found that the NEK7 kinase-dead mutant did not rescue the microtubule dynamics and growth cone turning from *Nek7* knockdown, showing that they depend on the catalytic activity of NEK7; intriguingly, neurite length and branching points were rescued (Figure S7). These different rescue outcomes could be explained by previously reported residual activity of the kinase-dead mutant (O’Regan and Fry, 2009) or by a secondary function of NEK7 independent of its kinase activity.

### PV+ Interneuron Axon Complexity and Wiring Require NEK7 Function *In Vivo*

**Figure 6:**
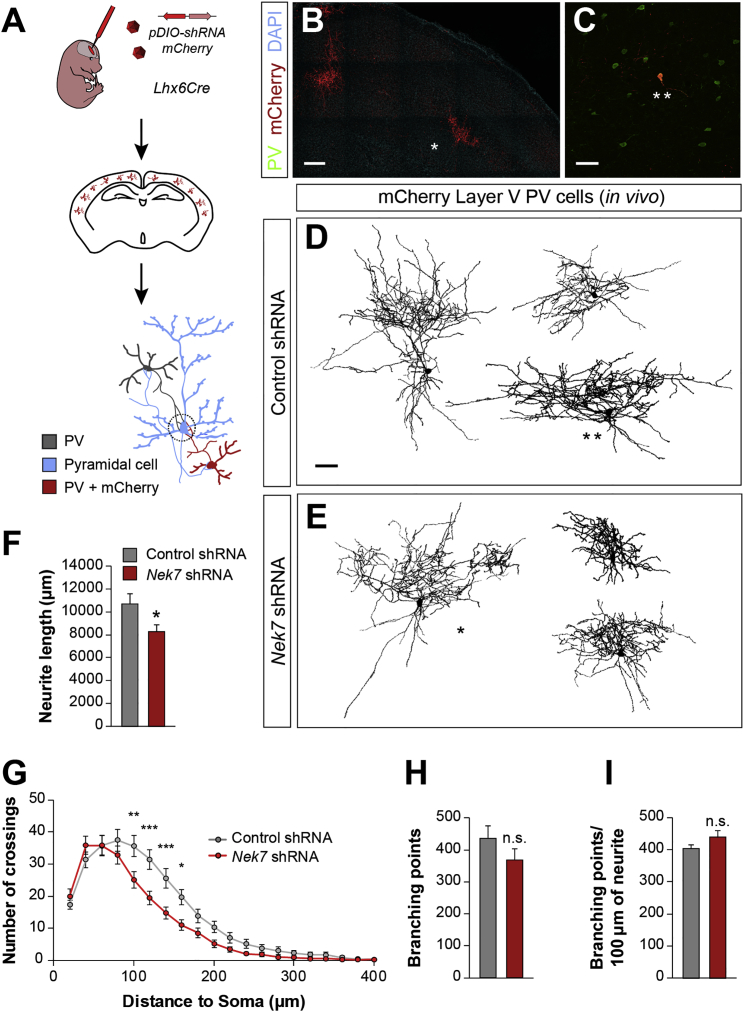
Loss of NEK7 Causes Abnormal PV+ Neuronal Morphology *In Vivo* (A) Schematic of experimental design. Cre-dependent AAVs expressing shRNA and the fluorescent marker mCherry were injected in E15.5 *Lhx6Cre* mice *in vivo*. (B and C) Targeted mCherry+ interneurons (B) that were PV+ (C) were selected for the analysis. (D and E) Confocal Z projections of PV+ interneurons expressing control shRNA (D) and *Nek7* shRNA (E) reported by mCherry. The cells were automatically reconstructed and masked in black from layer V of somatosensory cortex at P21. (F–I) Average of total neurite length (F), Sholl analysis (G), total branching points (H), and branching points per 100 μm of neurite (I) of control shRNA (n = 30 PV+ neurons, from 10 mice) and *Nek7* shRNA (n = 27 PV+ neurons, from 8 mice) infected neurons. One-way ANOVA (F, H, and I) and two-way ANOVA with Bonferroni correction (G). ^∗^p < 0.05, ^∗∗^p < 0.01, and ^∗∗∗^p < 0.001; n.s., not significant. Data are represented as mean ± SEM. Scale bars represent 200 μm (B) and 50 μm (C–E). See also Figure S6.

To investigate whether NEK7 regulates PV interneuron axon development *in vivo*, we introduced Cre-dependent conditional vectors expressing *Nek7* shRNA or control shRNA in adeno-associated viral vectors (AAV) and validated the ability of *Nek7* shRNA to knockdown *Nek7* levels *in vivo* (Figures S6C–S6G). We next performed *in utero* ventricular viral injections in *Lhx6Cre* mice (Figure 6A), which allow a sparse infection of the virus to efficiently target isolated PV+ cells for subsequent reconstruction of their neurite arbor labeled with mCherry (Figures 6B and 6C). We found that the total neurite length of *Nek7*-knockdown cells was reduced compared with control neurons (Figures 6D–6F). In addition, we observed a consistent decrease in neurite complexity in the distal neurites (Figures 6D, 6E, and 6G). Because PV+ cell dendrites are simpler compared with their axons (Ascoli et al., 2008), the observed morphological deficits were likely illustrating an impaired axon development. In contrast to our *in vitro* data, axonal branching parameters were similar between conditions (Figures 6H and 6I). Altogether, these findings demonstrate that NEK7 is required for the normal development of PV+ interneuron axonal arbor.

**Figure 7:**
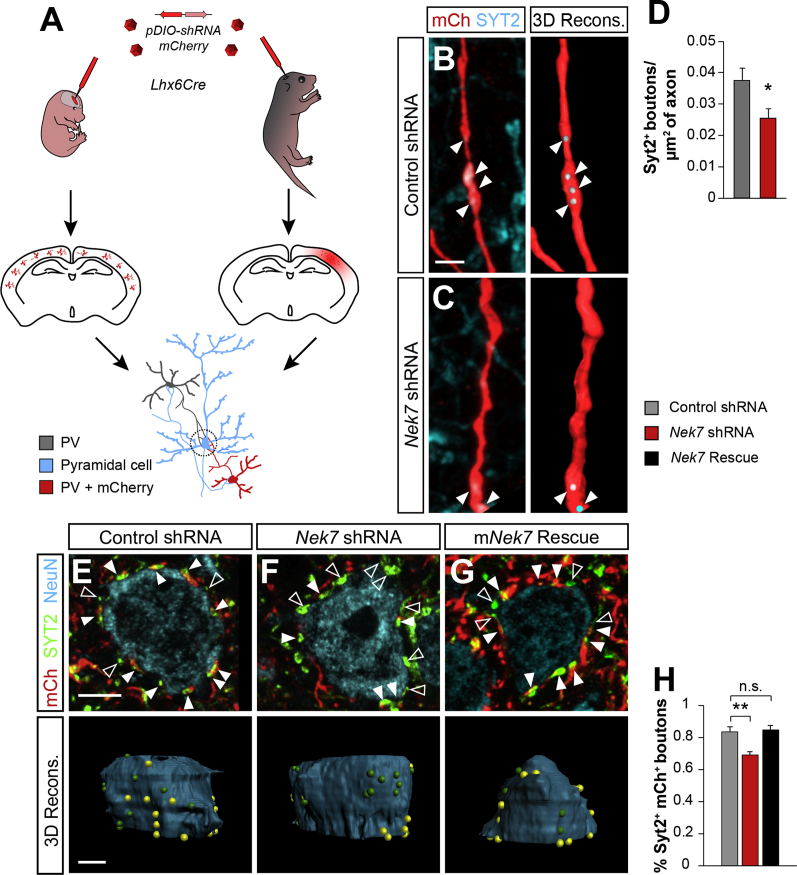
*Nek7* Knockdown Decreases PV+ Interneuron Outputs (A) Schematic of the experimental design. Cre-dependent virus expressing shRNA and the fluorescent marker *mCherry* were injected in E15.5 (left) and P3 (right) *Lhx6Cre* mice *in vivo*. Boutons co-stained with SYT2 and mCherry were quantified either inside PV+ arbors or onto NeuN+ somata. (B and C) Confocal Z projections and Imaris 3D reconstructions showing control shRNA (B) and *Nek7* shRNA (C) infected PV+ cells expressing mCherry (red) in axons containing SYT2+ boutons (blue). (D) SYT2+ bouton density per unit area of neurite comparing control shRNA (n = 24 PV+ neurons from four mice) and *Nek7* shRNA (n = 22 PV+ neurons from six mice). Student’s t test. (E–G) Confocal images and 3D Imaris reconstructions showing mCherry+ (red), SYT2+ (green) synaptic boutons from infected PV+ cells expressing control shRNA (E), *Nek7* shRNA (F), and *Nek7* shRNA with m*Nek7* for rescue (G) contacting pyramidal cells NeuN+ (blue). SYT2+ mCherry+ (filled arrowheads, yellow spheres), SYT2+ mCherry− (open arrowheads, green spheres). (H) Percentage of SYT2+ mCherry+ somatic boutons contacting the pyramidal cells normalized to the percentage of PV+ cells infected in the area, comparing shRNA (n = 182 PV+ neurons from seven mice), *Nek7* shRNA (n = 200 pyramidal cells from six mice), and m*Nek7* (n = 109 pyramidal cells from four mice). Kruskal-Wallis test, pairwise comparisons. ^∗^p < 0.05 and ^∗∗^p < 0.01; n.s., not significant. Data are represented as mean ± SEM. Scale bars represent 2 μm (B and C) and 5 μm (E–G). See also Figure S6.

There is a strong correlation between axon development and synapse formation. Indeed, the growth of axonal arbors seems largely dictated by the formation of stable synaptic contacts (Alsina et al., 2001, Meyer and Smith, 2006, Ruthazer et al., 2006), and changes in the axonal arborization will influence the number of presynaptic inputs (Rico et al., 2004). To test whether the lack of NEK7 causes synaptic deficits, we first quantified the density of PV+ synaptic terminals (containing synaptotagmin 2, SYT2+ boutons) (Sommeijer and Levelt, 2012) in reconstructed PV+ cell arbors (Figure 7A). We found that PV+ interneurons lacking NEK7 had a lower density of SYT2+ synaptic boutons compared with control conditions (Figures 7B–7D). This result suggested that NEK7-deficient PV+ axons form fewer synaptic terminals than control interneurons.

Next, we investigated whether abnormal axon and synaptic terminal development may affect the final wiring of PV+ interneurons. To this end, we assessed the connectivity between PV+ interneurons and pyramidal cells by carrying out postnatal infections to target a large number of interneurons, as previously described (Favuzzi et al., 2017) (Figure 7A). We quantified SYT2+ boutons from mCherry+ targeted PV+ cells contacting NeuN+ somata. We observed that the percentage of PV+ cells infected in the area was linearly correlated with the percentage of SYT2+/mCherry+ boutons (Figure S6H), and we therefore normalized the number of mCherry+ boutons on each pyramidal cell soma to the percentage of infected PV+ cells in the area to compare across samples. We found a consistent decrease in the percentage of SYT2+/mCherry+ boutons in *Nek7*-knockdown interneurons compared with controls (Figures 7E–7H). The synaptic phenotype was consistently restored by m*Nek7* (Figures 7G, 7H, S6I, and S6J). Taken together, these results reinforce the notion that *Nek7* depletion from PV+ interneurons causes defects in axonal arborization and altered synaptic connectivity.

## Discussion

During postnatal development, pyramidal cells and interneurons undergo extraordinarily divergent transformations in their morphologies. Pyramidal cell dendrites acquire a very stereotyped polarity and complexity, and pyramidal cell axons follow a near-linear path toward their long-range targets (Spruston, 2008, Stepanyants et al., 2004). In contrast, interneuron dendrites are less elaborated than those of pyramidal cells, but their axonal arbors have a remarkably tortuous outgrowth to form dense networks of local connections (Jiang et al., 2015, Stepanyants et al., 2004). Although these morphological differences are very evident, little is known about the molecular mechanisms underlying their divergent development. In this study, we addressed this question by carrying out a high-throughput genetic screen to identify differentially expressed genes during the wiring of GABAergic interneurons, highlighting the kinase NEK7 as a regulator of PV+ interneuron connectivity. Loss of NEK7 from putative PV+ cells alters the dynamics of both microtubules and growth cones and causes an abnormal axonal arborization and a reduction in the number of PV+ synaptic inputs impinging onto pyramidal cells. Altogether, our results unveil a specific molecular mechanism by which the microtubule-associated kinase NEK7 instructs the wiring of PV+ interneurons.

Several transcriptome analyses in the mouse cerebral cortex have previously defined the molecular profiles of mature glutamatergic pyramidal cells and GABAergic interneurons (Sugino et al., 2006, Tasic et al., 2016). However, information is missing about the developmental emergence of the molecular programs that define the profound morphological and functional characteristics that distinguish pyramidal cells and interneurons. An elegant forward genetic screening *in vitro*, aiming to identify differentially expressed genes between glutamatergic and GABAergic neurons during synaptogenesis, described mostly common mediators for both types of synapses rather than specific regulators of each type (Paradis et al., 2007). Our study unveils a set of 133 genes enriched in interneurons during axon development and synapse formation *in vivo*. The validation of NEK7, one of the most prominent candidates, as a regulator of interneuron axon development supports our approach to identify molecules involved in GABAergic wiring, thus providing a solid database to further expand this analysis to other candidate genes.

NEK7 has been previously involved in the formation of microtubule-based structures and the regulation of microtubule dynamics in cell lines and fibroblasts (Cohen et al., 2013, Kim et al., 2007, O’Regan and Fry, 2009, Salem et al., 2010, Yissachar et al., 2006). Specifically, loss of NEK7 decreases the growth and catastrophe rates of the microtubules in these cells (Cohen et al., 2013). In the cerebral cortex, *Nek7* transcripts are expressed mainly in PV+ interneurons, which are characterized by very complex and unique axonal arbors (Dumitriu et al., 2007, Jiang et al., 2015, Tremblay et al., 2016). As in non-neuronal cells, NEK7 also regulates microtubule dynamics in PV+ interneurons and, as a consequence, axon development. Interestingly, we have found that not only is the expression of *Nek7* specific to interneurons in the cerebral cortex, but its exogenous overexpression in pyramidal cells is not sufficient to change microtubule dynamics and axonal development in this population (Figure 5). This suggests that NEK7 is part of a broader molecular program present in interneurons but not in pyramidal cells. Further studies will be required to elucidate the molecular mechanisms by which NEK7 mediates microtubule dynamics in PV+ interneurons.

The motility and growth of axons in response to extracellular cues require precise interactions between the actin and microtubule cytoskeletons (Conde and Cáceres, 2009, Geraldo and Gordon-Weeks, 2009, Kalil and Dent, 2014). Although actin polymerization is the driving force of axon protrusion, it is only when microtubules invade the filopodia that those become axonal branches. Thus, changes in microtubule dynamics can alter the development of the axon (Conde and Cáceres, 2009). Consistent with this idea, we found that *Nek7* knockdown impairs microtubule dynamics, which leads to axons with altered growth cone steering.

Intriguingly, although microtubule dynamics and axonal behavior depend on NEK7 kinase activity, the arborization phenotype seems to be kinase independent. It is plausible that higher levels of the protein expressed by transfection together with its residual kinase activity (O’Regan and Fry, 2009) may not be sufficient to increase microtubule dynamics and axonal growth but could compensate the morphological phenotype after several DIV. Alternatively, NEK7 may contribute to the final formation of the axonal arbor through a molecular mechanism independent of its kinase activity, as kinase-independent interactions of NEK7 have been previously described (He et al., 2016). Future experiments targeting the dead-kinase domain at the endogenous locus will shed light on this interesting phenomenon.

We observed a decrease in the synaptic connections of PV+ interneurons onto pyramidal cells that might be independent of the length of the axons. Supporting this idea, synaptic structure and function are also sensitive to changes in microtubule dynamics (Roos et al., 2000). It is also plausible that shorter and misrouted axons with less elaborated arbors, as observed in NEK7-deficient PV+ cells, may have fewer opportunities to reach all the appropriated pyramidal cell targets to form synapses.

The precise function of NEK7 in microtubule dynamics and axonal wiring, together with its specific expression in PV+ cells, suggests that interneurons use specific mechanisms for axon growth and synaptic targeting that are not present in other cortical cells, including pyramidal cells. Compared with the axons of their neighboring pyramidal cells, cortical interneuron axonal paths are more tortuous and form a greater number of crossings with their synaptic targets (Huang et al., 2007, Stepanyants et al., 2004, Wierenga et al., 2008). An attractive explanation for these observations is that the assembly of GABAergic synapses might be mainly the outcome of the exploratory behavior of axons driven by a unique molecular program that is absent in pyramidal cells. It is tempting to speculate that a collection of proteins such as NEK7 confers unique behaviors to the PV+ axons that allow them to increase their axonal arbor complexity and their efficiency to establish specific connections with the highest possible number of neighbor pyramidal cells.

## Experimental Procedures

### Mice

Animal procedures were approved by ethical committees (IN-CSIC and King’s College London) and conducted in accordance with Spanish and European Union regulations and Home Office personal and project licenses under the UK Animals (Scientific Procedures) 1986 Act. All the experiments *in vivo* were performed in males, and gender was not distinguishable for primary cultures.

### FACS, Microarrays, and qPCR

Neurons expressing GFP were isolated by FACS (FACSAria II; BD Biosciences) and snap-frozen in liquid nitrogen to be kept at −80°C. Total RNA for microarray analysis and qPCR was extracted and purified using the RNeasy Micro extraction kit (QIAGEN) and Tri-reagent extraction method (Sigma-Aldrich) following the manufacturers’ instructions and hybridized to Mouse Gene 1.0 ST Array (Affymetrix).

Microarray fluorescence intensity data were analyzed using the R package BioConductor (Ihaka and Gentleman, 1996). Normalization was carried out with the algorithm Robust Multiarray Average (RMA). Finally, statistical significance for differential expression between the samples (IN P10 versus IN P0, PyrP12 versus Pyr P0, and IN P10 versus Pyr P12) was measured using significance analysis of microarrays (SAM), selecting genes with a false discovery rate (FDR) lower than 0.05 and a fold change greater than 2. Subsequently, these lists were compared among them to obtain genes specifically expressed in IN P10. The selected candidate was confirmed by qPCR.

### DNA Constructs, Viral Production, and Viral Injections

All shRNAs, including *Nek7* shRNA, were designed using Block-iT (Thermo Fisher Scientific) against the open reading frame (ORF) sequence adding EcoRI and *AvrII* enzymatic restriction sites at the 5′ and 3′ ends, respectively. Control shRNAs were designed against *gfp* and *lacZ* gene sequences, which are absent in the mouse. shRNAs were synthetized as single-stranded DNA (ssDNA) and annealed to their complementary strand before being cloned into the *pDIO-shRNA-mCherry* vector. The engineering of the *pDIO-shRNA-mCherry* vector has been described previously (Favuzzi et al., 2017).

To generate the *shRNA*-resistant NEK7 full-length (m*Nek7*), we introduced synonymous mutations in the shRNA targeting sequence (5′GAGAGAACCGTTTGGAAATAC-3′) and added 3 × FLAG sequences as a tag at the C-terminal end of the coding sequence. The synthetic double-stranded DNA (dsDNA) sequence (Strings-GeneArt Gene Synthesis Service; Thermo Fisher Scientific) was A′-tailed and cloned into the *pGEMT*-Easy Vector (Promega) and sub-cloned, after sequence verification, into the vector *pDIO-Cheta-TdTomato* (37755; Addgene). The CDS *Cheta-TdTomato* sequence was substituted by m*Nek7* using *AscI* and *NheI*.

The coding sequence for EB3 was PCR-amplified from a plasmid kindly provided by Dr. Niels Galjart (Erasmus MC) and sub-cloned into *pILES-YFP* vector to produce the *pILES-EB3-YFP* as previously described (Geraldo et al., 2008).

To obtain isolated infected cells for morphological reconstructions, we injected 1 μL of AAVs diluted 1:30, in PBS with FastGreen 0.5%, into the telencephalic lateral ventricle of E15.5 *Lhx6Cre* embryos. To increase the yield of infection for the analysis of PV+ wiring in pyramidal cells, P3–P4 mice were anesthetized with isoflurane and placed in a stereotaxic frame. For detailed procedures, see Supplemental Experimental Procedures.

### Primary Cultures and Transfection

Cortices from *Nkx2-1Cre* or *NexCre* embryos at E17.5–E18.5 were dissected, and cells were dissociated with trypsin (1 mg/mL, 15 min at 37°C) followed by gentle mechanical trituration as described previously (Rico et al., 2004). For detailed procedures, see Supplemental Experimental Procedures.

### Immunohistochemistry, Immunocytochemistry, and *In Situ* Hybridization

Mice were anesthetized and perfused with PBS followed by 4% paraformaldehyde (PFA) in PBS. Similarly, cortical cultures were fixed in 4% PFA and 4% sucrose diluted in culture media during 20 min at 12 DIV (interneurons) or 8 DIV (pyramidal cells) for immunocytochemistry. For detailed procedures, see Supplemental Experimental Procedures.

For dual-color fluorescence *in situ* hybridization combined with immunohistochemistry, mice were perfused with 4% PFA in PBS and the dissected brains fixed overnight in the same solution. Brains were then cut at 30 μm, and free-floating coronal sections were subsequently hybridized with digoxigenin-labeled probes and antibodies as described previously (Favuzzi et al., 2017). Probe sequence against *Nek7* was obtained from the Allen Brain Atlas database (Lein et al., 2007) and amplified using the following primers: RP_050725_01_H03, 5′-CGGAGGAGCTACGACAGC-3′, 5′-TGACTATCACGCCAGGCA-3′. Nitro blue tetrazolium (NBT)/5-bromo-4-chloro-3-indolyl phosphate (BCIP) colorimetric *in situ* hybridization was performed as described previously (Flames et al., 2007).

### Image Acquisition and Analysis

Images were acquired at 1,024 × 1,024 pixel resolution, 8-bit depth, and 400 Hz in an inverted Leica TCS-SP8 confocal microscope, blind to the experimental condition. Image analysis was performed using Imaris 8.1.2 (Bitplane) after applying background subtraction and Gaussian filtering with this software. Time-lapse experiments were carried out in an incubation chamber to keep the cultures at 37°C and 4% CO_2_, and recordings were done at 7 DIV using a resonant scanner (15%) and a hybrid detector (8,000 Hz). For microtubule dynamics quantification, confocal image stacks (100×, 1.44 NA objective, digital zoom 2.2, 0.2 μm step size) were taken at 2 s intervals during 2 min and pixel resolution was reduced to 512 × 512 to increase the scanning speed. For growth cone dynamics analysis, confocal image stacks (20×, 0.5 NA objective, digital zoom 4.0, 1 μm step size) were taken at 2.5 min intervals during 1 hr.

For *in vitro* morphological reconstructions, neurons were imaged with a 20× objective (0.5 NA, digital zoom 0.75, 600 Hz, 1 μm z-step size). Conversely, *in vivo* morphologies were imaged with a 63× objective (1.4 NA, digital zoom 0.75 at 400 Hz, 0.5 μm z-step size). Although we could not confirm PV identity *in vitro* because of the early time point of the primary cultures that benefit the trace of isolated axons, we confirmed that the neuron was positive for PV *in vivo*, before any morphological reconstruction.

For synaptic bouton quantifications, the same 100× objective was used to image 10 μm z stacks from the surface of the slice. For the synapses in isolated neurons, once PV+ cells were identified, random regions of the neurite arbor targeted with a Cre-dependent *lacZ* shRNA or *Nek7* shRNA reported by mCherry were imaged, and a surface was generated with the mCherry channel.

Single plane confocal images were acquired with a 40× objective (1.3 NA, digital zoom 0.75) and cell density and colocalization quantified with the Cell Counter plug-in for ImageJ (NIH). Cells were quantified independently in the different channels and then compared to identify co-expression of two markers. The same experimental procedure was used for the *in situ* colocalizations of *Nek7*.

### Statistical Analysis

Statistical analysis was performed using SPSS software (IBM). To obtain unbiased data, experimental mice from the different conditions were processed together, and quantifications were performed blind to the experimental condition. Data were analyzed with parametric tests, Student’s t test or ANOVA, when datasets met assumptions of normality (Kolmogorov-Smirnov test) and homoscedasticity (Levene test). In ANOVA, this was followed by Bonferroni post hoc analysis for comparisons of multiple conditions. Non-parametric tests for independent groups were applied when normality was not met (Mann-Whitney, Kruskal-Wallis). For the Kruskal-Wallis test, the Dunn-Bonferroni post hoc test was used for comparison of multiple samples. Sholl analysis data were analyzed using two-way ANOVA with Bonferroni correction. Finally, the Mantel-Haenszel χ^2^ test was used to compare the population distributions. Data are expressed as mean ± SEM. Population distributions are expressed as total cell percentages. Differences were considered statistically significant at p < 0.05.

## Data and Software Availability

The accession number for the microarray data reported in this paper is GEO: GSE115948.

## Supplemental Information

Document S1. Supplemental Experimental Procedures, Figures S1–S7, and Table S1

Document S2. Article plus Supplemental Information
