# Supplementary material for: The Microtubule Regulator NEK7 Coordinates the Wiring of Cortical Parvalbumin Interneurons
Source: Cell Rep. 2018 Jul 31;24(5):1231–42. doi: 10.1016/j.celrep.2018.06.115 (PMC6088228; doi:10.1016/j.celrep.2018.06.115)
Supplement: Document S1. Supplemental Experimental Procedures, Figures S1–S7, and Table S1 [file mmc1.pdf]

Cell Reports, Volume *24*

Supplemental Information

The Microtubule Regulator NEK7 Coordinates the Wiring of Cortical Parvalbumin Interneurons

Antonio Jesús Hinojosa, Rubén Deogracias, and Beatriz Rico

SUPPLEMENTAL INFORMATION

SUPPLEMENTAL FIGURES

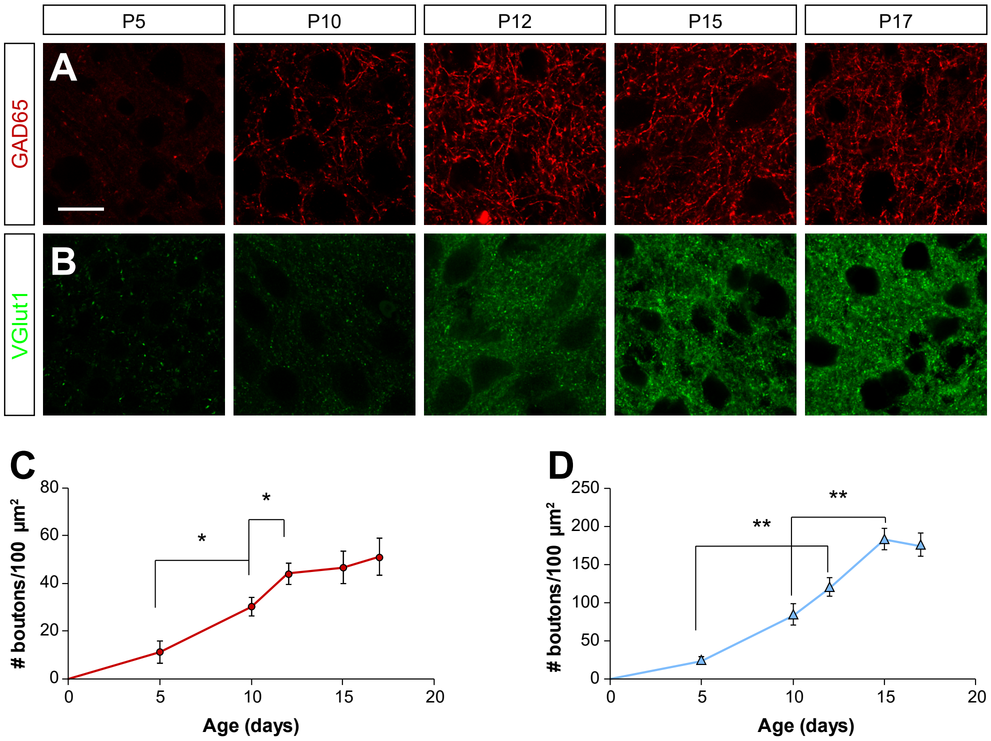

Figure S1. Time course of the development of GABAergic and glutamatergic inputs. Related to Figure 1.
(A and B) Single confocal images showing GAD65 (A, red) and VGlut1 (B, green) immunostaining during postnatal development in layer II/III of wild-type somatosensory cortex.
(C and D) GAD65 (C) and VGlut1 (D) bouton density (n = 3 mouse per age).
One-way ANOVA, Post hoc Bonferroni, * $p < 0.05$, ** $p < 0.01$. Data are represented as mean ± SEM. Scale bar represents 10 µm.

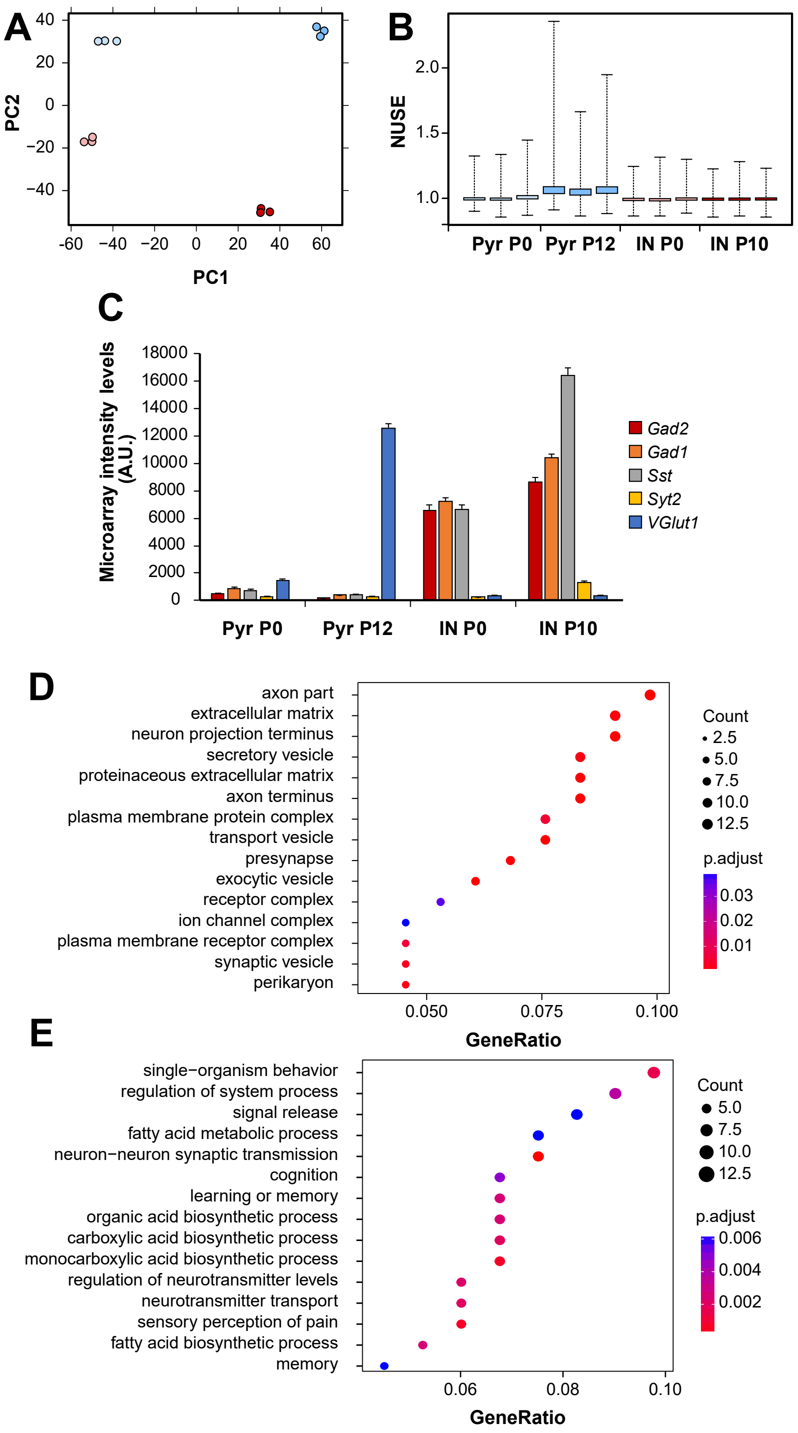

Figure S2. Quality control experiments for microarrays and gene ontology (GO). Related to Figure 1.
(A) Scatterplot showing the distribution of the different microarray condition replicates along the first two principal components.
(B) Boxplot showing the error of the median intensity values for each probe-set normalized to 1 (NUSE, Normalized Unscaled Standard Error).
(C) Microarray intensity levels of cell population specific markers [Interneurons: Gad1 (GAD67), Gad2 (GAD65), Sst and Syt2; pyramidal cells: Slc17a7 (VGlut1)] (A.U.: arbitrary units).
(D and E) Representative categories of the GO analysis. Ratio of genes from the filtered gene set belonging to the most representative categories of the cellular (D) and biological (E) component domain.
Data are represented as mean ± SEM.

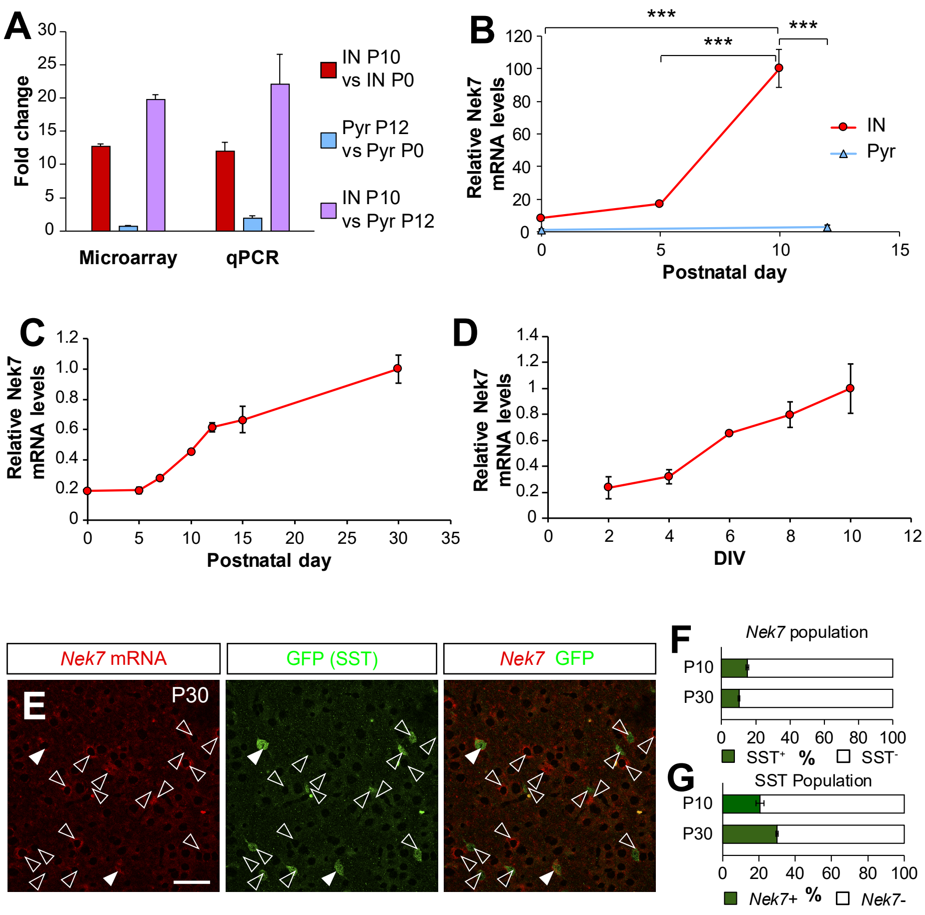

Figure S3. ***Nek7*** **transcript expression levels along development. Related to Figure 1.**
(A) Fold change values from comparisons between the different experimental conditions using Microarray and qPCR analyses.
(B) *Nek7* mRNA levels measured by qPCR in FACs sorted neurons from *Nkx2-1Cre;RCE* (IN, n = 3 mice per age) and *NexCre;RCE* (Pyr, n = 3 mice per age) relative to 18S.
(C) *Nek7* mRNA levels normalized to 18S in whole cortices at different postnatal stages (n = 4 mouse per age). All data is normalized to the average of the higher age.
(D) Same as in C for different DIV (n = 3 cultures per age).
(E) Confocal images showing in situ hybridization for *Nek7* (red) and immunohistochemistry for GFP (green) in the somatosensory cortex of P30 *SSTCre;RCE* mice. Filled arrowheads: colocalization, open arrowheads: no colocalization.
(F) Percentage of SST+ (Green) among all *Nek7*-expressing cells at P10 and P30.
(G) Percentage of *Nek7* positive cells among all SST expressing neurons at P10 and P30.
For representative pictures of P10 SST and *Nek7* staining refer to figure 1D.
One-way ANOVA, Post hoc Bonferroni, *** $p < 0.001$, Data are represented as mean ± SEM.

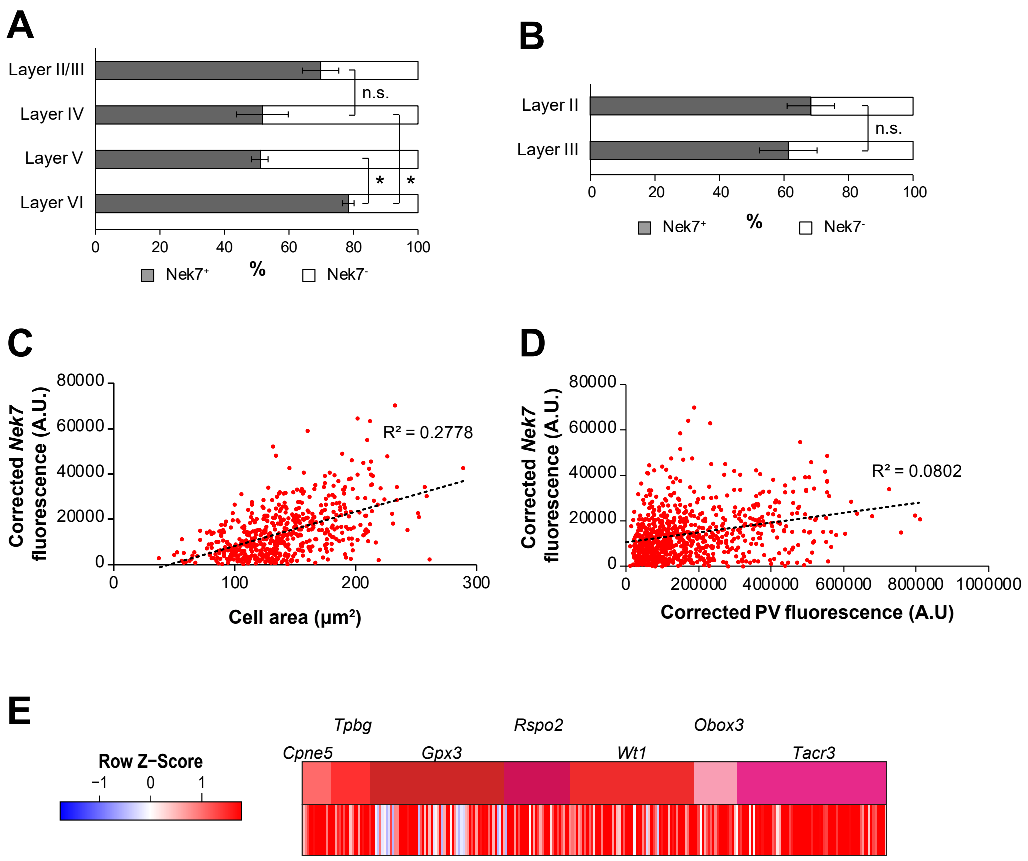

Figure S4. *Nek7* seems to be expressed in most types of PV+ cells. Related to Figure 1.
(A, B) Percentage of *Nek7* positive cells among PV expressing neurons across cortical layers in the somatosensory cortex of P30 wild-type mice. Layer II-III was divided in half to consider putative layer II and III.
(C) *Nek7 in situ* fluorescence intensity as a function of somatic area of PV+ cells.
(D) *Nek7 in situ* fluorescence as a function of PV fluorescence.
(E) Heatmap showing the expression levels of Nek7 in individual PV cells (lower row) from different genetic clusters (upper row). Row Z-score: standard deviation from the mean intensity values. (Data extracted from Tasic *et al.*, 2016).
Data are represented as mean ± SEM (A, B) or individual values for each cell (C, D).

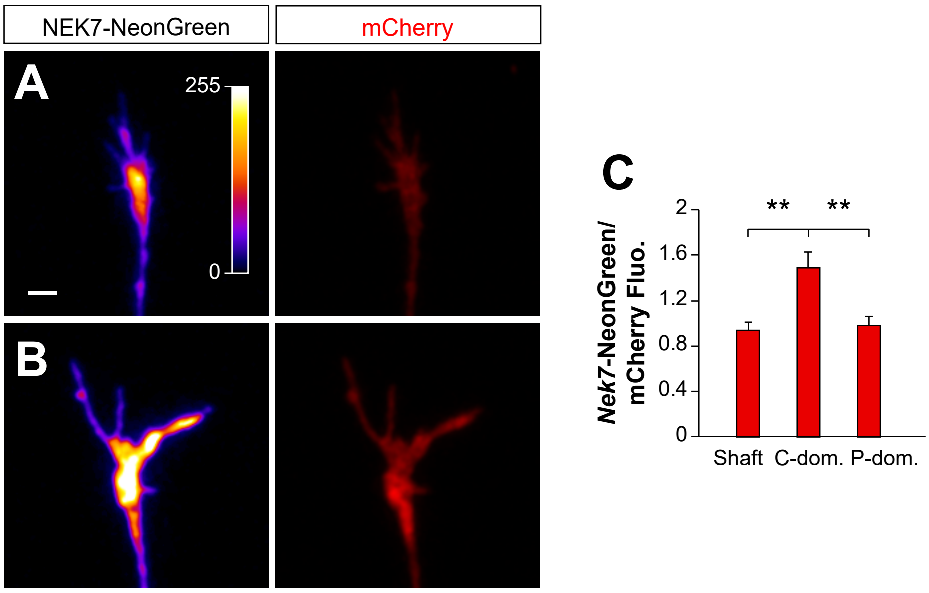

Figure S5. NEK7 is enriched in the central domain of growth cones. Related to Figure 2.

(A, B) Confocal Z-projection frames of two examples of Nkx2-1Cre growth cones co-expressing NEK7 NeonGreen (pseudocolor) and mCherry (red).

(C) NEK7 NeonGreen fluorescence intensity normalized to mCherry fluorescence in different regions of the growth cone (Shaft: axonal shaft, C-dom: central domain, P-dom: peripheral domain).

Kruskal-Wallis test, pairwise comparisons, ** $p < 0.01$. Data are represented as mean ± SEM.

Scale bar represents 2 µm.

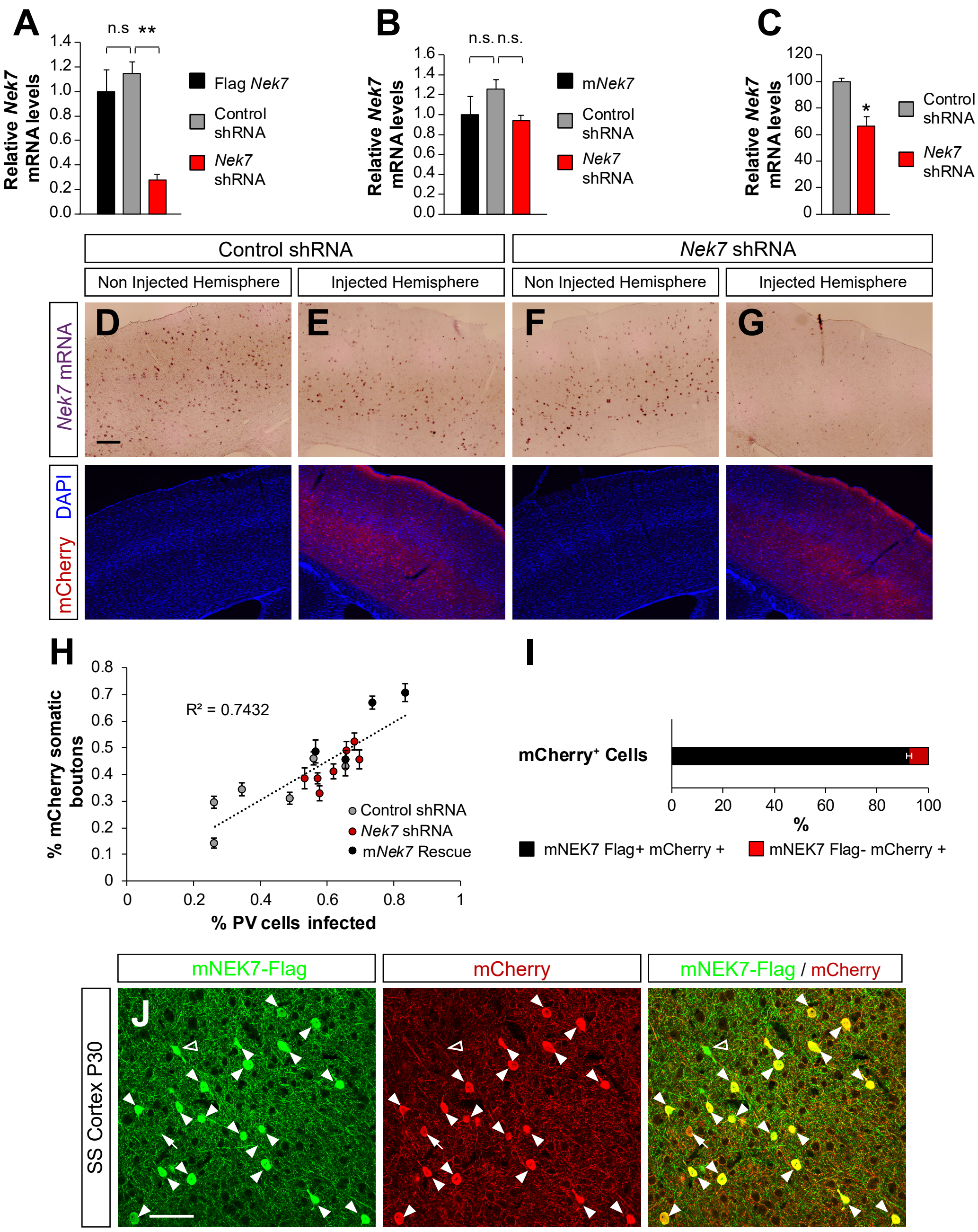

Figure S6. *Nek7* shRNA validation assays. Related to Figures 2, 3, 4, 6, 7.
(A) *Nek7* mRNA levels in HEK293 cells transfected with plasmids containing Cre, and the Cre-dependent plasmids Flag Nek7 alone (black, n = 5 cultures) or together with control shRNA (grey, n = 4 cultures) or *Nek7* shRNA (red, n = 5 cultures). Levels are relative to the non-shRNA condition (black).
(B) Same experimental conditions than (A) but using the synonymously mutated form of *Nek7* (m*Nek7*) alone (black, n = 5 cultures) or together with control shRNA (grey, n = 4 cultures) or *Nek7* shRNA (red, n = 3 cultures).
(C) *Nek7* mRNA levels in dissected cortices from P30 *Lhx6Cre* mice infected with an AAV containing control shRNA (n = 3 mice) or *Nek7* shRNA (n = 4 mice).
(D-G) Top panels, colorimetric in situ hybridization illustrating *Nek7* mRNA expression in P30 *Lhx6Cre* mice of non-injected hemispheres (D and F) and the contralateral hemispheres injected with AAV viruses expressing control shRNA (E) and *Nek7* shRNA (G). Bottom panels, contiguous coronal sections to the ones used in the top panels showing the area of infection labeled by mCherry (red). One-way ANOVA, Post hoc Bonferroni (A, B), Mann-Whitney test (C). * $p < 0.05$, ** $p < 0.01$, n.s. not significant.
(H) Percentage of mCherry+ somatic boutons (SYT2+) as a function of the percentage of PV+ cells infected in the area.
(I) Percentage of mCherry+ expressing cells that co-express m*Nek7* (Flag+) during coinfection of both AAVs.
(J) Single confocal images showing Flag+NEK7 (green) and mCherry+ (red) immunostaining in the somatosensory cortex of P30 Lhx6Cre mice coinfected with an AAV expressing *Nek7* shRNA and m*Nek7*. Colocalizing cells (filled arrowheads), mCherry- NEK7+ (open arrowheads). Data are represented as mean ± SEM.
Scale bars represent 200 µm (D-G) and 50 µm (J).

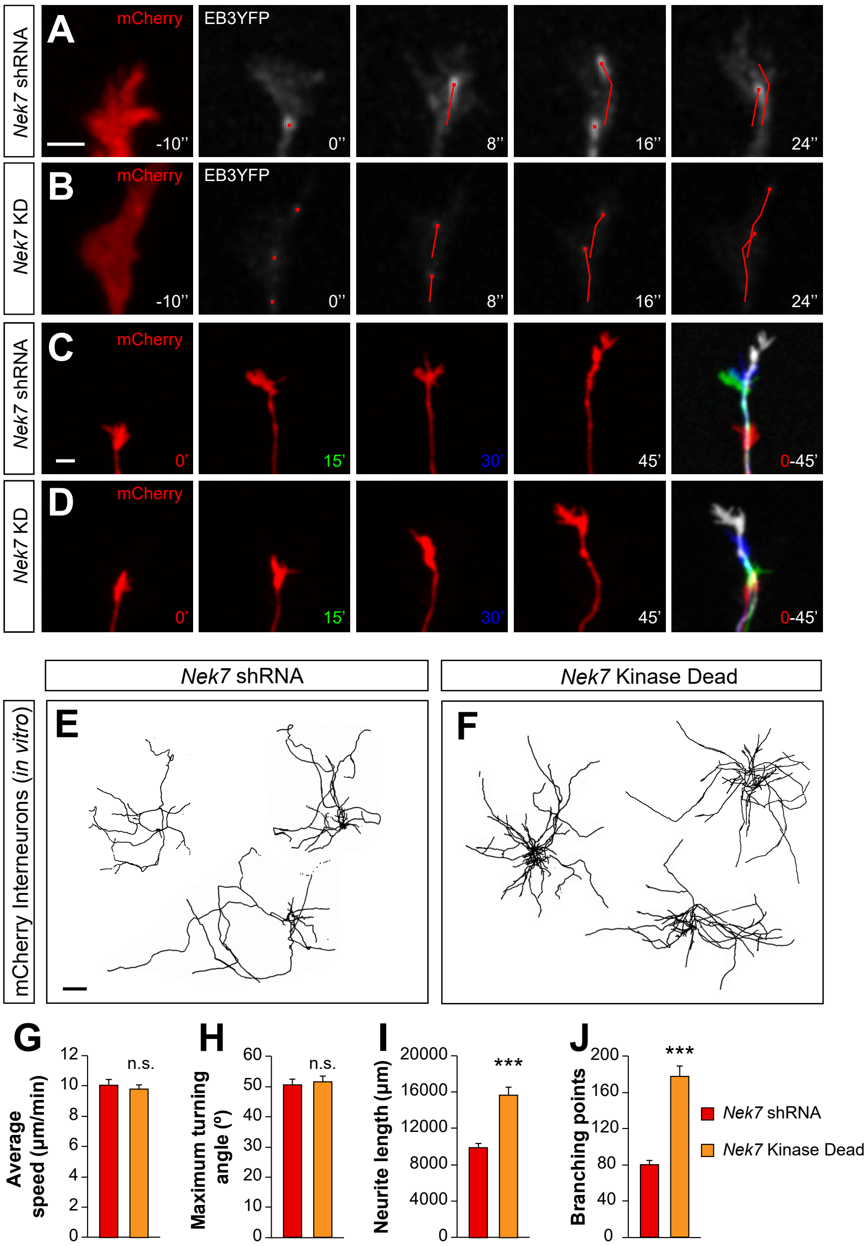

Figure S7. NEK7 kinase activity regulates different aspects of interneuron axonal development. Related to Figures 2, 3, 4.

(A, B) Confocal Z-projection frames from *Nkx2-1Cre* growth cones expressing mCherry (red, before the time-lapse) and EB3-YFP (gray, time-lapse). The path of EB3-YFP comets is tracked with a red line.

(C, D) Confocal Z-projection frames from *Nkx2-1Cre* growth cones. The last frame in the sequence shows superimposed images of the frames t = 0 min (red), t = 15 min (green), t = 30 min (blue) and t = 45 min (white).

(E, F) Confocal Z-projections of *Nkx2-1Cre* interneurons expressing mCherry. The cells were automatically reconstructed and masked in black at 12 DIV.

(G) Average speed of EB3-comets comparing *Nek7* depleted cells (A, n = 35 growth cones) and *Nek7* KD (B, n = 39 growth cones) from 3 independent cultures.

(H) Average of growth cone maximum turning angle from cells expressing *Nek7* shRNA (C, n = 59 growth cones), and *Nek7* KD (D, n = 75 growth cones) from 3 independent cultures.

(I-J) Neurite length (I) and branching points (J) comparing *Nek7* shRNA (E, n = 45 cells) and *Nek7* KD (F, n = 43 cells) transfected neurons from 3 independent cultures.

One-way ANOVA (G), Kruskal-Wallis test, pairwise comparisons (H-J). *** $p < 0.001$, n.s. not significant. Note that we used the panels and data from controls showed in Figures 2, 3 and 4 for the statistical comparisons. *Nek7* KD: *Nek7* Kinase Dead. Data are represented as mean ± SEM.

Scale bars represent 2 µm (A, B), 5 µm (C, D) and 200 µm (E, F).

SUPPLEMENTAL TABLES

Table S1

		INP10 vs INP0		INP10 vs PyrP12		
Gene symbol	**Probeset**	**Q-value**	**Fold change**	**Q-value**	**Fold change**	**τ**
Tac1	10536363	0.044	17.900	0.039	61.607	0.971
Hapln1	10406519	0.044	27.553	0.039	15.673	0.954
Nek7	10358259	0.044	12.648	0.039	19.695	0.944
Rbp4	10467319	0.044	14.438	0.039	13.506	0.935
Crhbp	10411215	0.044	10.481	0.039	18.558	0.932
Lgals1	10425161	0.044	13.226	0.039	17.109	0.931
Akr1c18	10407435	0.044	7.571	0.039	18.970	0.919
Pcp4l1	10360053	0.044	10.955	0.039	9.737	0.907
Th	10569370	0.044	8.353	0.039	8.551	0.882
Hpse	10531737	0.044	7.935	0.039	8.167	0.872
Gjc3	10534960	0.044	4.770	0.039	8.438	0.859
Sparc	10386058	0.044	4.282	0.039	7.682	0.847
Sst	10438730	0.044	2.487	0.039	45.753	0.845
Ret	10547227	0.044	6.475	0.039	6.853	0.842
Igf1	10365559	0.044	4.006	0.039	9.341	0.841
Cnp	10381154	0.044	3.692	0.039	7.468	0.837
Gpx3	10376201	0.044	4.649	0.039	6.088	0.832
Gjd2	10485979	0.044	3.607	0.039	6.528	0.823
Syt2	10350077	0.044	6.159	0.039	5.317	0.823
Slc32a1	10478124	0.044	2.378	0.039	18.729	0.821
Penk	10511363	0.044	7.371	0.039	3.847	0.815
Nrsn2	10488617	0.044	6.333	0.039	3.761	0.814
Grin2d	10563421	0.044	3.223	0.039	7.047	0.812
Moxd1	10362186	0.044	3.790	0.039	5.691	0.811
Pdyn	10487613	0.044	5.898	0.039	4.309	0.810
Mybpc1	10371627	0.044	5.144	0.039	4.897	0.803
Gpr83	10583286	0.044	5.038	0.039	4.653	0.799
Trmt2b	10606658	0.044	4.504	0.039	5.032	0.798
Zcchc12	10599187	0.044	2.612	0.039	8.489	0.798
Calb2	10581654	0.044	8.527	0.039	4.040	0.796
Nmbr	10361818	0.044	5.284	0.039	3.773	0.789
Oprd1	10516852	0.045	3.501	0.039	4.171	0.773
Pacsin2	10430997	0.044	4.949	0.039	3.109	0.769
Cnn2	10364593	0.044	3.602	0.039	4.961	0.766
Akr1b10	10537157	0.044	4.526	0.039	3.183	0.764
Adamts15	10591988	0.044	4.362	0.039	4.234	0.761
Ndrg2	10419578	0.045	2.510	0.039	7.708	0.759
Pnoc	10420853	0.044	3.341	0.039	5.230	0.758
Rassf4	10547177	0.044	3.435	0.039	5.002	0.758
Zfp804a	10473244	0.044	4.963	0.039	4.639	0.755
Lpl	10572130	0.044	16.734	0.039	2.390	0.750
Pdlim3	10571601	0.044	3.929	0.039	3.855	0.750
Mme	10492355	0.044	2.335	0.039	10.416	0.750
Cthrc1	10423836	0.044	3.551	0.039	3.857	0.747

Gpr37l1	10358023	0.045	2.745	0.039	4.659	0.746
Frmpd1	10504534	0.044	4.364	0.039	3.801	0.744
Alk	10452734	0.044	2.643	0.039	4.851	0.741
Sytl5	10598603	0.044	2.613	0.039	5.664	0.741
Filip1	10595298	0.044	4.430	0.039	2.740	0.729
Nnmt	10593219	0.044	3.796	0.039	3.562	0.724
Cplx1	10532180	0.044	4.292	0.039	2.718	0.723
Hap1	10391084	0.044	2.981	0.039	3.258	0.720
Ankrd29	10457536	0.044	3.376	0.039	3.274	0.719
Fam134b	10423333	0.044	4.010	0.039	2.596	0.719
Eya1	10353192	0.044	1.977	0.039	5.906	0.718
Vwc2	10374315	0.044	2.021	0.039	6.188	0.714
Pip5kl1	10471443	0.044	3.628	0.039	2.957	0.711
Igsf11	10435733	0.045	2.763	0.039	2.986	0.710
Ch25h	10467136	0.044	3.515	0.039	3.028	0.708
Ankrd34b	10406590	0.044	3.935	0.039	2.651	0.704
Cldn1	10438769	0.044	4.623	0.039	4.588	0.702
Egln3	10400304	0.044	2.301	0.039	6.583	0.701
Stac2	10390560	0.044	2.137	0.039	8.648	0.700
Adamts5	10440534	0.046	1.959	0.039	5.354	0.699
Btbd11	10365428	0.044	2.345	0.039	6.688	0.695
Arhgef6	10604713	0.044	2.447	0.039	4.205	0.694
Rab3b	10506883	0.044	2.805	0.039	3.261	0.693
Rcn1	10485645	0.044	3.935	0.039	2.581	0.692
Ptgs1	10471721	0.044	2.797	0.039	3.213	0.692
Gpr149	10498441	0.044	3.193	0.039	3.908	0.689
Klf3	10522051	0.044	2.136	0.039	6.698	0.685
Nacc2	10480901	0.044	2.290	0.039	3.502	0.685
Dmrt2	10462231	0.045	3.200	0.039	3.910	0.684
Pvt1	10424404	0.044	3.920	0.039	2.164	0.684
Ifit2	10462613	0.050	2.230	0.039	3.915	0.682
Thbs2	10447951	0.044	4.505	0.039	2.048	0.681
Ppif	10413222	0.044	2.884	0.039	3.283	0.681
Lypd6	10472034	0.044	2.586	0.039	3.589	0.680
Zim1	10559790	0.044	2.872	0.039	3.548	0.680
Flt3	10535780	0.044	3.764	0.039	2.261	0.675
Sfrp2	10492798	0.044	2.382	0.039	7.359	0.670
Grm1	10367830	0.044	2.394	0.039	3.162	0.666
Nefh	10383920	0.044	3.776	0.039	2.069	0.666
Id4	10404975	0.044	2.137	0.039	5.512	0.666
Kcnab1	10492402	0.044	8.561	0.039	2.133	0.665
Slc27a2	10475653	0.044	2.916	0.039	2.907	0.660
Aldh5a1	10408335	0.044	2.825	0.039	2.476	0.657
Doc2b	10388465	0.044	4.076	0.039	3.027	0.655
Alcam	10439895	0.044	3.266	0.039	2.335	0.651
Ndst3	10501924	0.044	5.214	0.039	2.368	0.645
Zfp385a	10433104	0.044	2.313	0.039	2.702	0.644
Myo5b	10456653	0.044	3.914	0.039	2.844	0.643

Phactr2	10367945	0.044	2.099	0.039	3.154	0.643
Ppapdc1a	10558049	0.044	2.180	0.039	3.363	0.642
Pgm2	10506188	0.044	2.049	0.039	2.787	0.640
Alox8	10387257	0.044	3.051	0.039	2.397	0.638
Rragb	10602692	0.044	3.048	0.039	2.288	0.636
Optn	10479833	0.044	3.082	0.039	2.670	0.634
Btn2a2	10408185	0.044	2.898	0.039	2.533	0.631
Adamts6	10406982	0.044	2.278	0.039	3.575	0.630
Drd2	10585169	0.044	2.433	0.039	3.023	0.628
Sema3c	10519886	0.045	3.229	0.039	2.020	0.619
Chrna2	10416071	0.045	2.605	0.039	2.591	0.619
Plch1	10498448	0.044	2.573	0.039	2.034	0.617
Crh	10497417	0.045	2.852	0.039	2.282	0.616
Pygb	10476969	0.044	3.053	0.039	2.589	0.616
Htr1d	10509238	0.044	2.567	0.039	2.414	0.608
Tmhs	10443383	0.044	2.706	0.039	2.633	0.606
Prdx3	10468869	0.046	2.663	0.039	2.242	0.604
Fam101b	10388488	0.044	2.716	0.039	2.010	0.603
Gys1	10552945	0.045	2.475	0.039	2.566	0.598
Acaa2	10456699	0.044	2.991	0.039	3.493	0.598
Kcnh2	10528548	0.044	2.146	0.039	2.885	0.591
Wif1	10366653	0.044	2.383	0.039	2.409	0.591
Dgkk	10598251	0.044	2.690	0.039	2.102	0.585
Tac2	10367024	0.048	2.440	0.039	2.167	0.582
Uprt	10601328	0.045	2.548	0.039	2.196	0.581
Acadl	10355246	0.044	2.529	0.039	2.074	0.579
Lypd6b	10472022	0.044	2.051	0.039	2.565	0.575
Shisa9	10433618	0.044	2.862	0.039	2.002	0.572
Fam195a	10449142	0.044	2.441	0.039	2.006	0.562
Prss23	10565456	0.044	2.218	0.039	2.168	0.561
Tacr1	10539244	0.044	2.092	0.039	2.440	0.558
C330016O10Rik	10385709	0.044	2.013	0.039	2.702	0.555
Rerg	10548899	0.044	2.217	0.039	2.989	0.554
Arhgap18	10362294	0.044	2.450	0.039	2.242	0.554
Baiap3	10448878	0.044	2.056	0.039	2.354	0.552
Fam150b	10395136	0.045	1.980	0.039	2.095	0.528
Fam43a	10434932	0.045	2.030	0.039	2.065	0.519
Hpcal1	10394778	0.045	2.052	0.039	2.531	0.511
Timp3	10365482	0.044	2.732	0.039	3.302	0.509
Luzp2	10553537	0.044	2.114	0.039	3.882	0.486
Rgag1	10602196	0.044	2.185	0.039	2.747	0.107

Table S1. Differentially and specifically upregulated genes during GABAergic wiring. Related to Figure 1. List of 133 genes upregulated during GABAergic wiring (IN P0 versus IN P10) and specifically expressed in interneurons (IN P10 versus Pyr P12). Pairwise comparisons were done using Significance Analysis of Microarrays (SAM) and genes with a false discovery rate (FDR) lower than 0.05 and a fold change higher than 2 were selected. The table reports: gene symbol, microarray probeset number, q-value (adjusted p-value), fold change for both comparisons, and specificity ratio for IN P10 (τ). Genes are sorted according to τ.

SUPPLEMENTAL EXPERIMENTAL PROCEDURES

Mice

Mice were maintained in a C57BL/6J background. *Nkx2-1Cre* (Sussel et al., 1999) and *NexCre* (Goebbels et al., 2006) mice were crossed with the Cre dependent reporter line R26R CAG-boosted EGFP (*RCE:LoxP*) (Miyoshi et al., 2010) for the genomic screening to label interneurons and pyramidal cells respectively. *Nek7 in vivo* loss of function experiments were carried out in *Lhx6Cre* mice (Fogarty et al., 2007), which were always kept in heterozygosis. Heterozygous *Nkx2-1Cre* mice were used for the primary cortical cultures. Homozygous Sst-IRES-Cre mice (Taniguchi et al., 2011) were crossed with *RCE:loxP* for colocalization with *Nek7* transcript.

FACS, microarrays and qPCR

For the FACS experiments, mice were anesthetized with sodium pentobarbital and the somatosensory cortex, in *NexCre;RCE:LoxP*, and whole neocortex, in *Nkx2-1Cre;RCE:LoxP* (to increase cell yield), were dissociated as described before (Catapano et al., 2001). Two to six mice were collected per run until having a minimum of 10^5 cells per microarray. The total number of cells per condition was a follows: for pyramidal cells, *NexCre;RCE,* $1.6 \cdot 10^6$ and $5.7 \cdot 10^5$ for P0 and P12, respectively; for interneurons, *Nkx2-1Cre;RCE*, $1.3 \cdot 10^6$ and $6.9 \cdot 10^5$ for P0 and P10, respectively. For RNA purity validation, microarray hybridization and bioinformatic analysis, RNA samples were sent to the genomics facility of "Centro de Investigación del Cancer" at University of Salamanca. Three replicates were done for all the conditions assayed. Briefly, 100-300 ng of total RNA were amplified using the WT Expression Kit (Ambion), labelled using the GeneChip WT Terminal Labeling Kit (Affymetrix) and hybridized to Mouse Gene 1.0 ST Array (Affymetrix).

Given the expression of *Nkx2-1* in a population of oligodendrocytes (Nery et al., 2001), we filtered our final list to remove genes specifically expressed in oligodendrocytes during early postnatal development (Cahoy et al., 2008). The final 133 genes were ranked according to the specificity ratio (τ) in IN P10. The 20 first genes were selected and scored according to both their specificity ratio and the total levels in IN P10. τ was calculated as described before (Kryuchkova-Mostacci and Robinson-Rechavi, 2017). Finally, gene lists were analyzed for possible known roles in the literature using Gene Ontology (Ashburner et al., 2000). To do this the R-package ClusterProfiler was used in R (Yu et al., 2012). A q-value cut off of 0.05 was applied in all the analysis carried out and the 15 most significant categories are shown.

RNA samples used in the quantitative PCR (qPCR) experiments were retronstranscribed using SuperScript IV according to manufacturer instructions (ThermoFisher Scientific). qPCR was carried out in triplicates using SYBR Green PCR Master Mix (Roche) on a LightCycler 96 Instrument (Roche). Normalized mRNA levels of *Nek7* relatively to those of 18S or mCherry were calculated using the number of cycles to reach a threshold signal (Cq) according to the $2^{-\Delta\Delta Cq}$ equation. qPCR primer sequences were as follows: *Nek7* (5'-AGCCACAGAAGGCATTACGG-3', 5'-CTACCGGCACTCCATCCAAG-3'), 18S (5'- GTAACCCGTTGAACCCCATTCGT-3', 5'-GTGTGTACAAAGGGCAGGGACTTAA-3') and mCherry (5'-CATCCTGTCCCCTCAGTTCATG-3', 5'- CATCCTGTCCCCTCAGTTCATG-3').

DNA constructs, viral production and viral injections

The ssDNA oligonucleotides to generate the shRNAs were: *Nek7* shRNA (5'-CTAGGAGAGAACCGTTTGGAAATACCCTGACCCAGTATTTCCAAACGGTTCTCTCTTTTTG-3' and 5'-AATTCAAAAAGAGAGAACCGTTTGGAAATACTGGGTCAGGGTATTTCCAAACGGTTCTCTC-3'), gfp shRNA (5'-CTAGGCCACAACGTCTATATCATGGCCTGACCCACCATGATATAGACGTTGTGGCTTTTTG-3' and 5'-AATTCAAAAAGCCACAACGTCTATATCATGGTGGGTCAGGCCATGATATAGACGTTGTGGC-3') and *lacZ* shRNA (5'-CTAGAAATCGCTGATTTGTGTAGTCCCTGACCCAGACTACACAAATCAGCGATTTTTTTG-3' and 5'-AATTCAAAAAAAATCGCTGATTTGTGTAGTCTGGGTCAGGGACTACACAAATCAGCGATT

T-3').

NeonGreen was PCR amplified from a plasmid containing the gene (Allele biotechnology, Shaner et al., 2013), a Flag tag was added and it was subcloned in the C-terminus of m*Nek7* using *AscI* and *Sph1*. The kinase dead mutation was generated through targeted mutagenesis of m*Nek7* (K63M/K64M, (O'Regan and Fry, 2009).

We used the described constructs to generate AAVs (serotype rep2/cap8) as described previously (Favuzzi et al., 2017). Briefly, 80% confluent HEK293FT cells (ThermoFisher Scientific) were transfected with *pDIO-shRNA-mCherry* or m*Nek7* and AAV8 capside plasmid (pDP8.ape vector, PlasmidFactory) with polyethylenimine (PEI, SigmaAldrich) at a DNA:PEI ratio of 1:4. Viruses were harvested after three days, purified by ultracentrifugation in iodixanol gradients and filtered-concentrated using Amicon Ultra Centrifugal Filters 100 kDa (Millipore). Only AAV batches with a titer higher than 1 x 10^{12} viral genome copies/mL were used for *in vivo* experiments. For morphological reconstructions, we obtained isolated cells by injecting 1 mL of AAVs diluted 1:30, in PBS with FastGreen 0.5%, into the telencephalic lateral ventricle of E15.5 *Lhx6Cre* embryos. Pregnant females were deeply anesthetized with isoflurane (5% induction, 2% maintenance), the abdominal cavity was cut opened and the uterus exposed making the embryos accessible. After injection, the uterus was placed back in the abdominal cavity, the incision was sutured and buprenorphine 0.1 mg/Kg was used for analgesia. For the analysis of PV+ wiring in pyramidal cells, we increased the yield of infection by performing postnatal AAV stereotaxic injections in P3-P4 *Lhx6Cre mice*. After making a small incision in the skull (2.2 mm lateral and 2.6 mm anterior to interaural midpoint), two injections of 250 nL each containing these same viruses diluted 1:1 were infused at 50 nL/min using a Nanoinjector2010 (WPI).

Primary cultures and transfection

Cells were resuspended in supplemented Neurobasal A medium (Invitrogen) with 500 mM Glutamax-L, 2% v/v B-27 and 0.01 U/mL penicillin/streptomycin; and plated at a density of 100.000 cells/cm^2 on glass-bottom plates (MatTek corporation) previously coated with 0.5 mg/mL Poly-L-lysine (Sigma-Aldrich) overnight at 37ºC. Cultures at day *in vitro* 4 (4 DIV) were transfected with *Nek7* shRNA or *lacZ* shRNA using Lipofectamine 2000 (ThermoFisher Scientific) during 3 hours at a final total concentration of 0.5 μg/mL of DNA on the plate according to manufacturer's instructions. To assay microtubule dynamics, plasmids expressing EB3-YFP were co-transfected with the shRNA plasmids (ratio 1:3) using the same culture conditions as described above.

Rescue experiments were carried out by co-transfection of Cre-dependent *Nek7* shRNA together with m*Nek7* or *Nek7* KD at a ratio 1:1. To assay the effect of Nek7 in pyramidal cells, Cre-dependent constructs expressing m*Nek7* or an empty vector together with mCherry were co-transfected in *NexCre* primary cultures (ratio 1:1). Finally, NEK7 dynamics was visualized co-transfecting Cre-dependent plasmids reporting mCherry and *Nek7* NeonGreen (ratio 1:1).

Immunohistochemistry and immunocytochemistry

P21 or P30 mice were anesthetized with sodium pentobarbital and perfused transcardially with PBS followed by 4% paraformaldehyde (PFA) in PBS. Coronal slices of 40 μm (or 100 μm for the morphological reconstructions) were cut in a sliding microtome (Leica) for immunohistochemistry. Free-floating brain sections were blocked with 5% BSA, 0.3% Triton and 10% normal donkey serum during 2 hours at room temperature and incubated with primary antibodies at 4ºC in 1% BSA, 0.3% triton and 5% normal donkey serum overnight (40 μm sections) or 2 days at room temperature (100 μm). The following day, sections were rinsed in PBS and incubated in the appropriated secondary antibodies for 2 hours (40 μm sections) or 4 hours (100 μm) at room temperature in the same solution as the primary antibodies. Finally, the slices were rinsed in PBS, incubated with DAPI and mounted in Mowiol/DABCO.

Fixed primary cultures were rinsed and permeabilized with 0.5% triton during 15 min. The plates were blocked in blocking solution (2% BSA and 2% normal donkey serum) during 1 hour and incubated with primary antibodies diluted in the same solution overnight. The following day they were rinsed in PBS, incubated with secondary antibodies for 1 hour, stained with DAPI and mounted with Mowiol/DABCO.

The following primary antibodies were used: rabbit anti-DsRed (1:500, Clontech 632496), mouse anti-Flag (1:500, Sigma F1804), goat anti-mCherry (1:500-1:1000, antibodies-online ABIN1440057), mouse anti-NeuN (1:500, Millipore MAB377), rabbit anti-PV (1:5000, Swant PV-25), chicken anti-PV (1:500, SySy 195 006), mouse anti-SYT2 (1:500, ZFIN ZDB-ATB-081002-25), chicken anti-GFP (Aves labs GFP-1020) and rat anti-SST (1:200, Chemicon MAB354). Secondary antibodies and Alexa-conjugated streptavidin were purchased from Molecular Probes (#A-21206, #A-31572, #A-21202, #A-31571, #A-21241, #A-21121, #A-21432, #A-21208), Jackson (#711-165-152, #016-470-084, #703-225-155) and Vector (#BA-9500).

Image acquisition and analysis

The number of mCherry+ SYT2+ boutons depends on the infection efficiency. Indeed, the proportion of SYT2+ mCherry+ boutons increased linearly with the number of PV+ infected (Fig S9A). For this reason, we normalized bouton percentages to the number of infected PV+ cells in the area (200 μm from the quantified pyramidal cell) (Fino et al., 2013).

To measure PV and NEK7 intensity levels in these *in situ* colocalizations, 12-bit images were acquired and fluorescence levels were measured with ImageJ. The Corrected fluorescence was calculated with the following formula:

Corrected fluo. = Integrated intensity- (Cell Area · Background mean fluo.)

NEK7 NeonGreen intensity levels *in vitro* were acquired in 8-bit and normalized with mCherry to correct for growth cone volume as described before (Myers et al., 2012).

For EB3 comets, Imaris spots were manually drawn on EB3-YFP comets in each frame and their tracks automatically quantified along time. For growth cone dynamics analysis, Imaris filaments were semi-automatically drawn in each frame and their tracks followed and quantified automatically along time. The turning angle was quantified as the angle formed between the growth cone center of mass and the underlying axon shaft. Neuronal morphology was automatically reconstructed by generating, first, an Imaris surface comprising the whole cell morphology, then producing a new masked channel containing exclusively the signal within this surface and, finally, automatically creating a filament with the signal coming from the masked channel.

For synaptic bouton quantification, Spots, putatively labeling PV synaptic terminals reported with SYT2 staining, were identified in the SYT2 channel (average diameter 0.5 μm) and the Imaris XTension "Split spots into surface" was used to quantify the synaptic boutons (spots) within the neurites. For the analysis of somatic SYT2 boutons onto pyramidal cells, we used the mCherry surface, SYT2 boutons and a surface from NeuN channel. First the XTension "Spots close to surface" was used to filter the spots at 0.25 μm from the NeuN surfaces, *i.e.* the somatic boutons. Subsequently, somatic boutons coming from infected cells were identified using "Split spots into surface".

SUPPLEMENTAL REFERENCES

Ashburner, M., Ball, C.A., Blake, J.A., Botstein, D., Butler, H., Cherry, J.M., Davis, A.P., Dolinski, K., Dwight, S.S., Eppig, J.T., et al. (2000). Gene ontology: tool for the unification of biology. The Gene Ontology Consortium. Nat. Genet. *25*, 25–29.

Cahoy, J.D., Emery, B., Kaushal, A., Foo, L.C., Zamanian, J.L., Christopherson, K.S., Xing, Y., Lubischer, J.L., Krieg, P.A., Krupenko, S.A., et al. (2008). A transcriptome database for astrocytes, neurons, and oligodendrocytes: a new resource for understanding brain development and function. J. Neurosci. 28, 264–278.

Catapano, L.A., Arnold, M.W., Perez, F.A., and Macklis, J.D. (2001). Specific neurotrophic factors support the survival of cortical projection neurons at distinct stages of development. J. Neurosci. *21*, 8863–8872.

Fino, E., Packer, A.M., and Yuste, R. (2013). The logic of inhibitory connectivity in the neocortex. Neuroscientist *19*, 228–237.

Fogarty, M., Grist, M., Gelman, D., Marín, O., Pachnis, V., and Kessaris, N. (2007). Spatial genetic patterning of the embryonic neuroepithelium generates GABAergic interneuron diversity in the adult cortex. J. Neurosci. 27, 10935–10946.

Goebbels, S., Bormuth, I., Bode, U., Hermanson, O., Schwab, M.H., and Nave, K.-A. (2006). Genetic targeting of principal neurons in neocortex and hippocampus of NEX-Cre mice. Genesis 44, 611–621.

Miyoshi, G., Hjerling-Leffler, J., Karayannis, T., Sousa, V.H., Butt, S.J.B., Battiste, J., Johnson, J.E., Machold, R.P., and Fishell, G. (2010). Genetic fate mapping reveals that the caudal ganglionic eminence produces a large and diverse population of superficial cortical interneurons. J. Neurosci. 30, 1582–1594.

Myers, J.P., Robles, E., Ducharme-Smith, A., and Gomez, T.M. (2012). Focal adhesion kinase modulates Cdc42 activity downstream of positive and negative axon guidance cues. J. Cell Sci. *125*, 2918–2929.

Nery, S., Wichterle, H., and Fishell, G. (2001). Sonic hedgehog contributes to oligodendrocyte specification in the mammalian forebrain. Development 128, 527–540.

Shaner, N.C., Lambert, G.G., Chammas, A., Ni, Y., Cranfill, P.J., Baird, M.A., Sell, B.R., Allen, J.R., Day, R.N., Israelsson, M., et al. (2013). A bright monomeric green fluorescent protein derived from Branchiostoma lanceolatum. Nat. Methods *10*, 407–409.

Sussel, L., Marin, O., Kimura, S., and Rubenstein, J.L. (1999). Loss of Nkx2.1 homeobox gene function results in a ventral to dorsal molecular respecification within the basal telencephalon: evidence for a transformation of the pallidum into the striatum. Development 126, 3359–3370.

Taniguchi, H., He, M., Wu, P., Kim, S., Paik, R., Sugino, K., Kvitsiani, D., Fu, Y., Lu, J., Lin, Y., et al. (2011). A resource of Cre driver lines for genetic targeting of GABAergic neurons in cerebral cortex. Neuron 71, 995–1013.

Yu, G., Wang, L.-G., Han, Y., and He, Q.-Y. (2012). clusterProfiler: an R package for comparing biological themes among gene clusters. OMICS *16*, 284–287.
