# Supplementary material for: The Microtubule Regulator NEK7 Coordinates the Wiring of Cortical Parvalbumin Interneurons
Source: Cell Rep. 2018 Jul 31;24(5):1231–42. doi: 10.1016/j.celrep.2018.06.115 (PMC6088228; doi:10.1016/j.celrep.2018.06.115)
Supplement: Document S2. Article plus Supplemental Information [file mmc8.pdf]

Cell Reports

Article

The Microtubule Regulator NEK7 Coordinates the Wiring of Cortical Parvalbumin Interneurons

Graphical Abstract

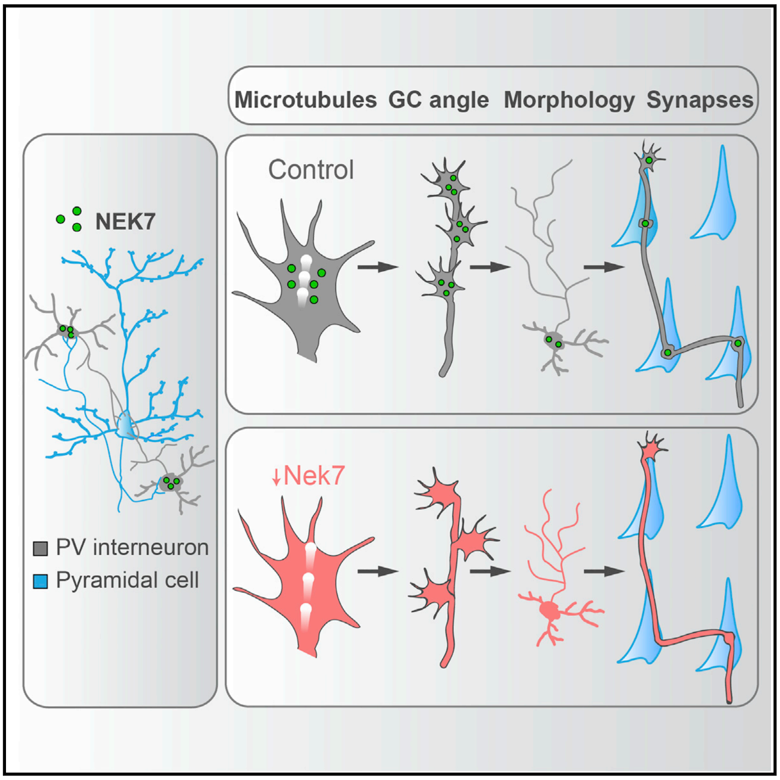

Authors

Antonio Jesús Hinojosa, Rubén Deogracias, Beatriz Rico

Correspondence

beatriz.rico@kcl.ac.uk

In Brief

Mature cortical circuits emerge during development by the integration of two morphologically distinguished neuronal populations, pyramidal cells and interneurons. Hinojosa et al. reveal a molecular program by which a microtubule-associated kinase specifically controls the shape and wiring of cortical interneurons.

Highlights

- *Nek7* is specifically expressed by GABAergic interneurons during cortical wiring
- Loss of *Nek7 in vitro* alters microtubule dynamics, axon steering, and morphology
- Loss of *Nek7 in vivo* impairs the morphology and synaptic outputs of PV interneurons

Data and Software Availability

GSE115948

Hinojosa et al., 2018, Cell Reports *24*, 1231–1242
July 31, 2018 Crown Copyright © 2018
https://doi.org/10.1016/j.celrep.2018.06.115

CellPress

Cell Reports
Article

OPEN ACCESS
CellPress

The Microtubule Regulator NEK7 Coordinates the Wiring of Cortical Parvalbumin Interneurons

Antonio Jesús Hinojosa,[1,2,3] Rubén Deogracias,[1,2,3] and Beatriz Rico[1,2,3,4,*]
[1]Centre for Developmental Neurobiology, Institute of Psychiatry, Psychology and Neuroscience, King's College London, London SE1 1UL, UK
[2]MRC Centre for Neurodevelopmental Disorders, King's College London, London SE1 1UL, UK
[3]Instituto de Neurociencias, Consejo Superior de Investigaciones Científicas & Universidad Miguel Hernández, Sant Joan d'Alacant 03550, Spain
[4]Lead Contact
*Correspondence: beatriz.rico@kcl.ac.uk
https://doi.org/10.1016/j.celrep.2018.06.115

SUMMARY

Functional networks in the mammalian cerebral cortex rely on the interaction between glutamatergic pyramidal cells and GABAergic interneurons. Both neuronal populations exhibit an extraordinary divergence in morphology and targeting areas, which ultimately dictate their precise function in cortical circuits. How these prominent morphological differences arise during development is not well understood. Here, we conducted a high-throughput screen for genes differentially expressed by pyramidal cells and interneurons during cortical wiring. We found that NEK7, a kinase involved in microtubule polymerization, is mostly expressed in parvalbumin (PV+) interneurons at the time when they establish their connectivity. Functional experiments revealed that NEK7-deficient PV+ interneurons show altered microtubule dynamics, axon growth cone steering and reduced axon length, arbor complexity, and total number of synaptic contacts formed with pyramidal cells. Altogether, our results reveal a molecular mechanism by which the microtubule-associated kinase NEK7 regulates the wiring of PV+ interneurons.

INTRODUCTION

Identifying the mechanisms by which neurons are precisely wired in specific circuits is critical to elucidate how the extraordinary complexity of brain function emerges. During development, wiring takes place through an ordered sequence of steps. The classical view of neural circuitry assembly proposes that axons first grow toward their target area, arborize through collateral extensions that are subsequently remodeled, and finally form synapses with the corresponding cellular targets (Kolodkin and Tessier-Lavigne, 2011; Shen and Scheiffele, 2010). These events are triggered by local extracellular cues, which instruct active crosstalk with different components of the cytoskeleton (Conde and Cáceres, 2009; Hoogenraad and Bradke, 2009; Kalil and Dent, 2014; Navarro and Rico, 2014).

There are two main classes of neurons in the mammalian cerebral cortex, glutamatergic pyramidal cells, which are excitatory projection neurons, and GABAergic interneurons, which form inhibitory local connections (Custo Greig et al., 2013; DeFelipe et al., 2002; Fishell and Rudy, 2011). The wide diversity of pyramidal cells and interneurons is defined by a unique set of neurochemical, morphological, connectivity, and firing features that are built by specific molecular programs. Among these features, one striking difference between pyramidal cells and interneurons is the morphology of their axons and dendrites. Pyramidal cell dendrites expand toward different cortical layers in a stereotyped fashion, and their axons typically target cells in other layers, as well as other cortical and subcortical areas. On the contrary, interneuron dendrites and axons very often remain in relatively close proximity to the cell soma. In addition, whereas the axons of pyramidal cells are straight, interneuron axons are profusely branched and follow convoluted paths that often correlate with the position of their postsynaptic targets (Dumitriu et al., 2007; Huang et al., 2007; Stepanyants et al., 2004; Tremblay et al., 2016). The molecular mechanisms regulating axon development and synapse formation in pyramidal cells have been studied in detail (de Wit and Ghosh, 2016; Conde and Cáceres, 2009; Kalil and Dent, 2014; Kolodkin and Tessier-Lavigne, 2011; McAllister, 2007; Navarro and Rico, 2014), but our understanding of these mechanisms in interneurons is very rudimentary. A large body of evidence suggests that small variations in the development of GABAergic interneurons may lead to neurodevelopmental disorders (Marín, 2012). Therefore, the identification of molecules that coordinate the development of these cells not only represents a major scientific advance but also has important biomedical implications.

Previous studies have identified genes that are differentially expressed among various populations of cortical neurons (Cembrowski et al., 2016; Sugino et al., 2006; Zeisel et al., 2015). However, how these unique molecular profiles emerge during development is not well understood. Here, we took a transcriptome approach to identify genes that are differentially expressed by GABAergic interneurons and pyramidal cells during the wiring of cortical circuits. We identify the never in mitosis A (NIMA)-related kinase *Nek7* (Morris, 1976; O'Connell et al., 2003) as one of the most enriched transcripts in interneurons during this critical period in cortical development. Targeted downregulation of *Nek7* disrupts microtubule and growth cone dynamics, alters

This is an open access article under the CC BY license (http://creativecommons.org/licenses/by/4.0/).

axonal arbor morphology, and causes a reduction in synaptic contacts made by parvalbumin (PV+) interneurons. Our study identifies NEK7 as an essential regulator of a molecular program that regulates microtubule dynamics and axon development in PV+ interneurons.

RESULTS

Identification of Differentially Expressed Genes during the Wiring of GABAergic Interneurons

Pyramidal cells and interneurons are remarkably different in morphology and synaptic targeting with distinct contributions to the cortical network. To understand how this heterogeneity emerges, we searched for differentially expressed genes between these two neuronal populations during cortical wiring. We selected the temporal window when cortical neurons develop axons and synapses to establish mature circuitries (Ben-Ari, 2002; De Felipe et al., 1997; Larsen and Callaway, 2006) (Figure S1). We used fluorescence-activated cell sorting (FACS) to isolate pyramidal cells and interneurons using mice reporting these populations with GFP at two different stages and subsequently carried out transcriptome analyses (Figure 1A). We performed an unsupervised principal-component analysis demonstrating that each replicate clusters with the corresponding conditions (Figure S2A). In addition, we found no outliers when comparing the overall distribution of signal intensities between microarrays (Figure S2B). To assess the purity of the isolated populations, we searched for genes that are known to be enriched in each of these cell types and upregulated between the different postnatal ages (Figure S2C; Table S1). We then carried out further bioinformatic analyses to search for genes differentially expressed in cortical interneurons compared with pyramidal cells and with higher expression at P10 than at P0 (Figure 1B). We identified a gene set of 133 transcripts with these characteristics (Table S1). The Gene Ontology (GO) database revealed that the most abundant categories included axon- and synapse-related structures, synaptic neurotransmission pathways, and genes encoding for extracellular matrix proteins (Figures S2D and S2E).

We next ranked the 133 differential expressed genes according to two criteria: the specificity ratio, defined by the normalized mean expression within the population of interneurons at P10 compared with the expression across other populations (Kryuchkova-Mostacci and Robinson-Rechavi, 2017), and the transcript expression levels in interneurons at P10 (Figure 1C). On the basis of these criteria, one of the first candidate genes was the serine/threonine kinase *Nek7*, which encodes a protein involved in microtubule dynamics (Cohen et al., 2013) (Figure 1C). qPCR experiments confirmed these findings (Figures S3A–S3D). Moreover, we found that half of *Nek7*-expressing cells are putative PV+ cells at P10 (52.4 ± 7.6%; Figures 1D and 1F) and that *Nek7* was expressed in a subpopulation of these cells (40.6 ± 2.8%; Figures 1D and 1G). We confirmed that the expression of *Nek7* was predominantly in PV+ interneurons at P30 (PV+, 77.0 ± 2.7%; Figures 1E and 1F; SST+, 10.2 ± 0.5%; Figures S3E–S3G) and that a large number of PV+ cells express *Nek7* (60.9 ± 2.0%; Figures 1E and 1G). Our observations are consistent with those of other studies showing that the majority of PV+ interneurons express *Nek7* transcripts (Nakajima et al., 2014; Tasic et al., 2016). Within the PV population, we found that the percentage of NEK7+ PV+ cells was significantly higher in layer VI than in other layers (Figures S4A and S4B). We did not find any correlation with the soma size or with the levels of PV, consistent with the previously reported expression of the kinase in all PV clusters (Figures S4C–S4E; Tasic et al., 2016). These results reveal that *Nek7* is a gene highly enriched in PV+ interneurons and upregulated during postnatal development, which suggests that it might play a role in the morphological differentiation and network integration of PV+ cells.

Nek7 Knockdown Accelerates Microtubule Dynamics and Alters Axon Steering

NEK7 has been shown to mediate microtubule-dependent processes in both dividing and non-dividing cells (Fry et al., 2012; Kim et al., 2007; Yissachar et al., 2006), probably through the regulation of microtubule dynamics (Cohen et al., 2013). Because microtubule dynamics are essential for axon and dendrite development in neurons (Conde and Cáceres, 2009; Dent et al., 2011; Gordon-Weeks, 2004; Kalil and Dent, 2014), we hypothesized that NEK7 could also regulate microtubule dynamics during the maturation of interneurons. Indeed, NEK7 was enriched in the central domain of interneuron growth cones, a subcellular compartment often populated by highly dynamic microtubules (tyrosinated microtubules) (Dent and Kalil, 2001) (Figure S5). To test this hypothesis, we explored the dynamic behavior of microtubules as reported by the plus-end-binding protein 3 (EB3) using *in vitro* time-lapse imaging (Stepanova et al., 2003). Similarly to *in vivo*, *Nek7* expression was also increasing during axonal development *in vitro* (Figure S3D). To downregulate the expression of *Nek7*, we engineered a Cre-dependent conditional vector expressing a short hairpin RNA (shRNA) against *Nek7* along with the fluorescent marker mCherry as a reporter of recombination (Figure 2A). We assessed the ability of *Nek7* shRNA to decrease *Nek7* levels *in vitro* in HEK cells expressing exogenous full-length *Nek7* (Figures S6A and S6B). To visualize local microtubule polymerization, we co-transfected *Nkx2-1Cre* primary cortical cultures at 7 days *in vitro* (DIV) with Cre-dependent plasmids for *Nek7* or a control shRNA and a plasmid encoding *EB3-YFP* (Figure 2A). Although *Nkx2-1Cre* does not exclusively drive recombination in PV+ cells (Maroof et al., 2010), it allows the recombination of the conditional constructs when the axons are still highly dynamic. We observed that downregulation of *Nek7* in interneurons increases the average speed of EB3-YFP comets compared with controls (Figures 2B–2H and Videos S1, S2, and S3). As expected, differences were observed only in a subpopulation of *Nkx2-1* growth cones, presumably in putative PV+ interneurons that normally express *Nek7* (Figure 2I). We confirmed that the abnormal microtubule behavior was specifically caused by Nek7 knockdown, because we rescued the average speed of EB3-YFP comets with dual infections of *Nek7* shRNA with an shRNA-resistant full-length NEK7 (m*Nek7*; Figures 2D, 2G, and 2H).

To examine whether the deficits in microtubule growth led to alterations in axon development, we explored the dynamic

OPEN ACCESS
CellPress

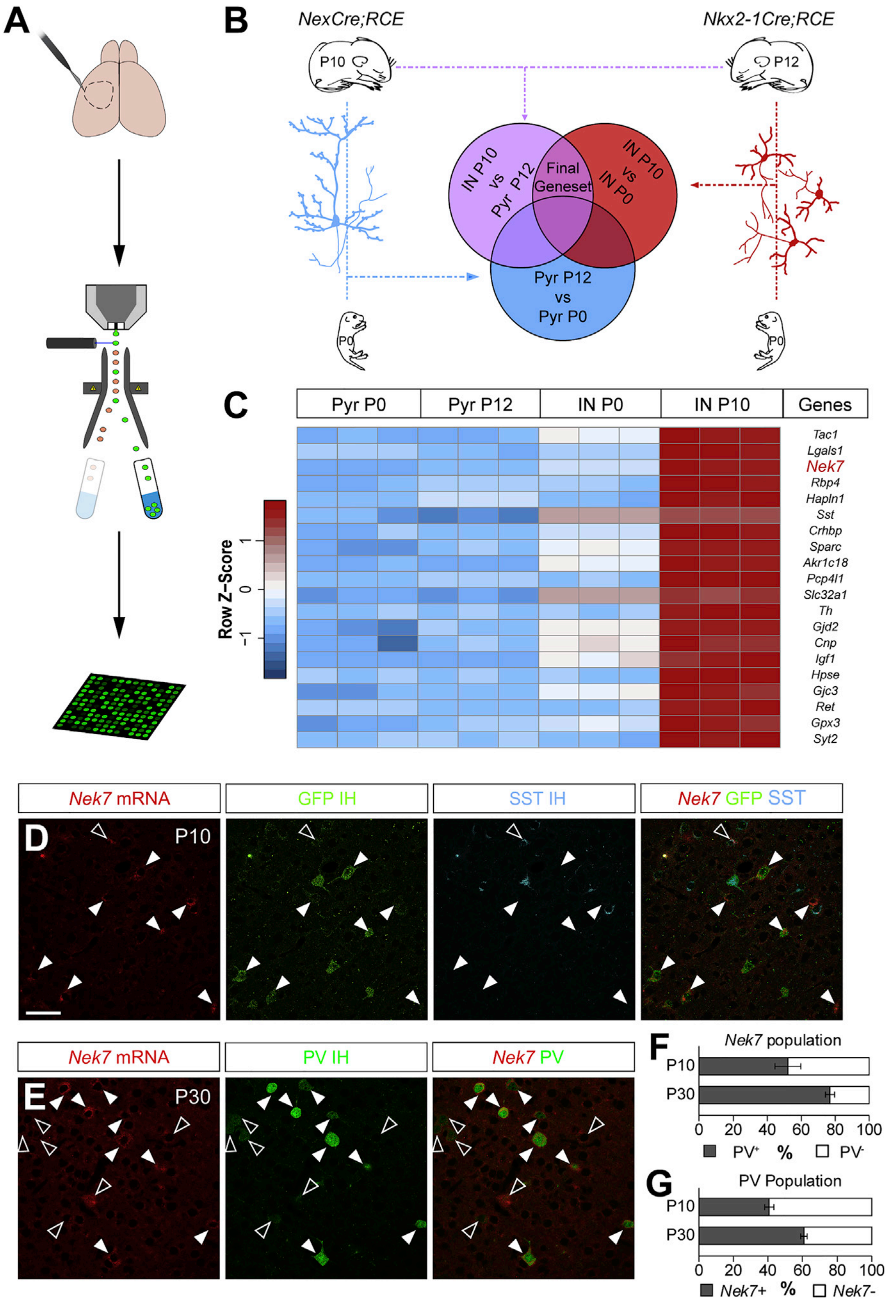

Figure 1. *Nek7* Transcripts Are Upregulated during GABAergic Interneuron Wiring and Expressed Mostly in PV+ Cells

(A) Schematic of genetic screening. Cortices from different GFP+ reporter lines were dissected, and cells were dissociated and FACS sorted and their RNA hybridized to microarrays.

(B) Bioinformatic comparisons of neuronal populations.

(C) Heatmap showing the expression levels of the 20 first ranked genes. *Nek7* is highlighted in red.

(D) Confocal images showing *in situ* hybridization for *Nek7* (red) and immunohistochemistry for GFP (green) and SST+ (cyan) in the somatosensory cortex of P10 *Lhx6Cre;RCE* mice. GFP+ and SST+ are somatostatin cells, GFP+ and SST− are putative PV+ interneurons.

(E) Confocal images showing *in situ* hybridization for *Nek7* (red) and immunohistochemistry for PV+ cells (green) in the somatosensory cortex of P30 wild-type mice. In (D) and (E), filled arrowheads denote colocalization, and open arrowheads denote no colocalization.

(F) Percentage of PV+ (gray) among all *Nek7*-expressing cortical cells at P10 and P30.

(G) Percentage of *Nek7*-positive cells among all PV-expressing neurons at P10 and P30. IN, interneurons (red); Pyr, pyramidal cells (blue). Data are represented as mean ± SEM. Scale bar represents 50 μm.

See also Figures S1–S4.

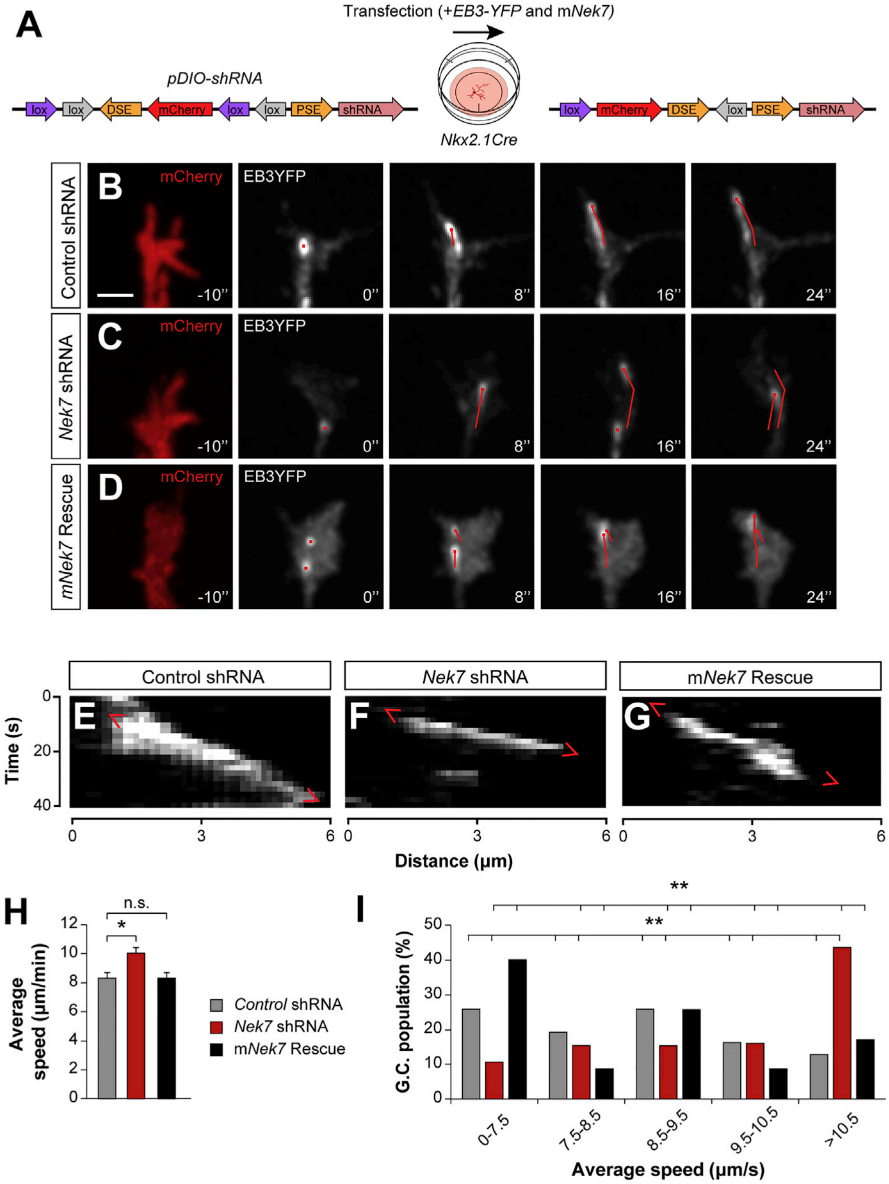

Figure 2. ***Nek7*** **Knockdown Accelerates Microtubule Growth**

(A) Diagram of the Cre-dependent constructs expressing *mCherry* and shRNA. The plasmids were co-transfected with *EB3-YFP*, and m*Nek7* for the rescue, in primary cortical cultures from *Nkx2-1Cre* mice at 4 DIV, and axons were recorded at 7 DIV.

(B–D) Confocal Z projection frames from control shRNA (B), *Nek7* shRNA (C), and rescue with m*Nek7* (D) *Nkx2-1Cre* growth cones expressing mCherry (red, before the time-lapse) and EB3-YFP (gray, time-lapse). The path of EB3-YFP comets is tracked with a red line. Scale bar represents 2 μm.

(E–G) Kymographs of EB3 comets in control (E), *Nek7* shRNA (F), and rescue (G) growth cones showing their existence time as a function of distance.

(H and I) Average speed (H) and speed distribution of EB3 comets (I) comparing control (n = 31 growth cones), *Nek7*-depleted cells (n = 35 growth cones), and m*Nek7* rescue cells (n = 35 growth cones) from three independent cultures. All growth cones with EB3 comets were quantified. One-way ANOVA (H) and χ^2 test (I). $^*p < 0.05$ and $^{**}p < 0.01$. Data are represented as mean ± SEM (H) or total cell percentage (I).

See also Figures S5–S7 and Videos S1, S2, and S3.

behavior of axons *in vitro* (Figure 3; Videos S4, S5, and S6). Although axon growth speed was similar between control and *Nek7* shRNA-expressing neurons, we observed that *Nek7*-depleted growth cones have increased turning angles compared with controls, which suggested a meandering behavior (Figures 3B–3F). This abnormal turning angle was also rescued by expressing m*Nek7* in interneurons (Figures 3D–3F). As before, differences in growth cone turning were observed only in a subpopulation of *Nkx2-1* neurons, most likely the *Nek7*-expressing PV+ interneurons (Figure 3G). Together, these results demonstrate that NEK7 regulates microtubule dynamics and axon steering in interneurons.

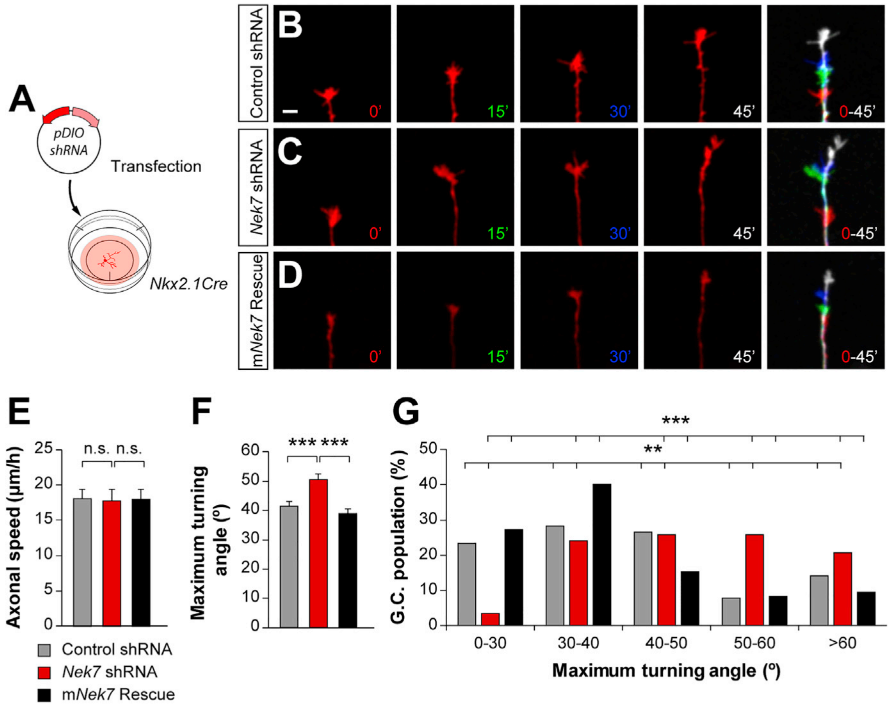

Figure 3. ***Nek7*** **Depletion Impairs Axonal Growth Cone Dynamics** ***In Vitro***

(A) Schematic of experimental design with the Cre-dependent plasmids transfected in *Nkx2-1Cre* primary cortical cultures at 4 DIV and axons recorded at 7 DIV.

(B–D) Confocal Z projection frames from axons expressing control (B), *Nek7* shRNA (C), and m*Nek7* rescue (D). The last frame in the sequence shows superimposed images of the frames t = 0 min (red), t = 15 min (green), t = 30 min (blue), and t = 45 min (white). Scale bar represents 5 µm.

(E–G) Axonal speed (E), average of growth cone maximum turning angle (F), and its distribution (G) from cells expressing control shRNA (n = 64 growth cones), *Nek7* shRNA (n = 59 growth cones), and m*Nek7* (n = 85 growth cones) from three or four independent cultures. All growth cones increasing in length were quantified. Kruskal-Wallis test, pairwise comparisons (E and F), and χ^2 test (G). Data are represented as mean ± SEM (E and F) or total cell percentage (G).
**p < 0.01 and ***p < 0.001; n.s., not significant. See also Figures S6 and S7 and Videos S4, S5, and S6.

NEK7 Function Is Required for the Morphological Development of Interneurons

Our previous results suggest that microtubule dynamics and axonal behavior in interneurons seem to depend on NEK7 function. Because interneuron axonal arbors are highly branched and convoluted (Dumitriu et al., 2007; Jiang et al., 2015; Tremblay et al., 2016), defects in axon dynamics may affect normal axonal morphology in these cells. To test whether NEK7 is required for interneuron axonal arborization, we transfected primary cortical cultures obtained from *Nkx2-1Cre* mice with control or *Nek7* shRNA expression vectors and compared the morphology of axonal arbors in both conditions (Figure 4A). We found that the average neurite length was significantly decreased in interneurons with *Nek7* knockdown compared with controls (Figures 4B–4E). As for other parameters, the reduction in neurite length was restricted to a fraction of the cells, presumably PV+ interneurons (Figure 4F). We found that axon complexity and the number of branches were also decreased in interneurons expressing *Nek7* shRNA compared with controls (Figures 4B, 4C, and 4G–4I). Consistently, the total neurite length, complexity, and axonal branches were rescued by m*Nek7* expression in interneurons (Figures 4D–4I). Our results demonstrate that NEK7 regulates different aspects of axon development in interneurons.

Given that pyramidal cells do not expressed NEK7 (Figures 1C, S3A, and S3B), we wondered whether NEK7 was sufficient to modify microtubule dynamics and axonal development if expressed ectopically in pyramidal cells. Specific overexpression of *Nek7* in pyramids showed similar microtubule dynamics, axonal behavior, and morphology as control pyramidal cells (Figure 5). Altogether, our findings reveal that NEK7 is part of an interneuron-specific molecular program orchestrating axon development.

Kinase-Dependent and Kinase-Independent Functions of NEK7 in Axon Development

NEK7 is a serine/threonine kinase with a highly conserved catalytic domain (Haq et al., 2015; O'Connell et al., 2003). To examine whether the catalytic activity of NEK7 was required for microtubule dynamics and axon behavior, we engineered a Cre-dependent *Nek7* kinase-dead mutant (*Nek7* KD) that was transfected in primary cultures as described above. We found that the NEK7 kinase-dead mutant did not rescue the microtubule dynamics and growth cone turning from *Nek7* knockdown, showing that they depend on the catalytic activity of NEK7; intriguingly, neurite length and branching points were rescued (Figure S7). These different rescue outcomes could be explained by previously reported residual activity of the kinase-dead

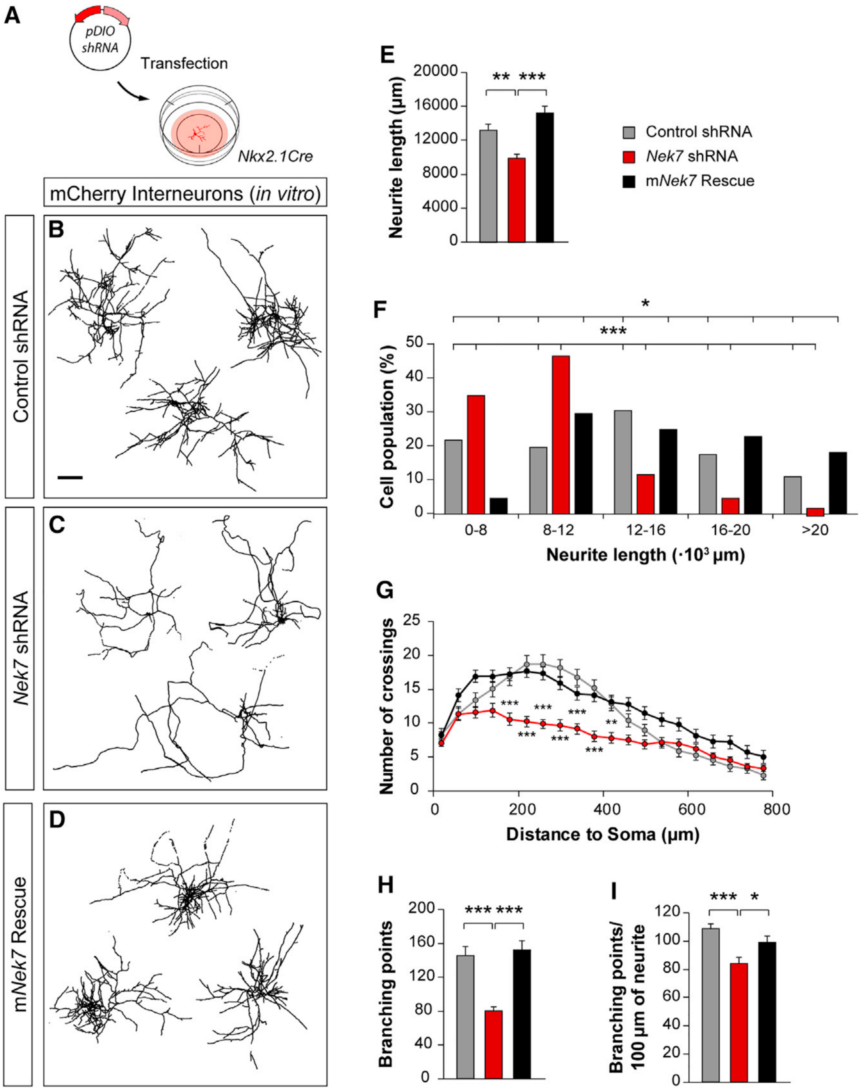

Figure 4. Loss of NEK7 Alters Interneuron Morphology *In Vitro*

(A) Schematic of experimental design. *pDIO-shRNA* was transfected in *Nkx2-1Cre* primary cortical cultures.

(B–D) Confocal Z projections of *Nkx2-1Cre* interneurons from cortical cultures transfected with control shRNA (B), *Nek7* shRNA (C), and m*Nek7* for rescue (D) expressing mCherry. The cells were automatically reconstructed and masked in black at 12 DIV. Scale bars represent 200 μm.

(E–I) Average of total neurite length (E) and its distribution (F), Sholl analysis (G), total branching points (H), and branching points per unit length (I) of control shRNA (n = 47), *Nek7* shRNA (n = 45), and rescue (n = 46) transfected neurons from three or four independent cultures. All imaged cells were quantified. Kruskal-Wallis test, pairwise comparisons (E, H, and I), χ^2 test (F), and two-way ANOVA with Bonferroni correction (G).

*p < 0.05, **p < 0.01, and ***p < 0.001. Data are represented as mean ± SEM. See also Figures S6 and S7.

mutant (O'Regan and Fry, 2009) or by a secondary function of NEK7 independent of its kinase activity.

PV+ Interneuron Axon Complexity and Wiring Require NEK7 Function *In Vivo*

To investigate whether NEK7 regulates PV interneuron axon development *in vivo*, we introduced Cre-dependent conditional vectors expressing *Nek7* shRNA or control shRNA in adeno-associated viral vectors (AAV) and validated the ability of *Nek7* shRNA to knockdown *Nek7* levels *in vivo* (Figures S6C–S6G). We next performed *in utero* ventricular viral injections in *Lhx6Cre* mice (Figure 6A), which allow a sparse infection of the virus to efficiently target isolated PV+ cells for subsequent reconstruction of their neurite arbor labeled with mCherry (Figures 6B and 6C). We found that the total neurite length of *Nek7*-knockdown cells was reduced compared with control neurons (Figures 6D–6F). In addition, we observed a consistent decrease in neurite complexity in the distal neurites (Figures 6D, 6E, and 6G). Because PV+ cell dendrites are simpler compared with their axons (Ascoli et al., 2008), the observed morphological deficits were likely illustrating an impaired axon development. In contrast to our *in vitro* data, axonal branching parameters were similar between conditions (Figures 6H and 6I). Altogether, these findings demonstrate that NEK7 is required for the normal development of PV+ interneuron axonal arbor.

There is a strong correlation between axon development and synapse formation. Indeed, the growth of axonal arbors seems largely dictated by the formation of stable synaptic contacts (Alsina et al., 2001; Meyer and Smith, 2006; Ruthazer et al., 2006), and changes in the axonal arborization will influence the number of presynaptic inputs (Rico et al., 2004). To test whether the lack of NEK7 causes synaptic deficits, we first quantified the density of PV+ synaptic terminals (containing synaptotagmin 2, SYT2+ boutons) (Sommeijer and Levelt, 2012) in reconstructed PV+ cell arbors (Figure 7A). We found that PV+ interneurons lacking NEK7 had a lower density of SYT2+ synaptic boutons compared with control conditions (Figures 7B–7D). This result suggested that NEK7-deficient PV+ axons form fewer synaptic terminals than control interneurons.

Next, we investigated whether abnormal axon and synaptic terminal development may affect the final wiring of PV+ interneurons. To this end, we assessed the connectivity between PV+ interneurons and pyramidal cells by carrying out postnatal infections to target a large number of interneurons, as previously described (Favuzzi et al., 2017) (Figure 7A). We quantified SYT2+ boutons from mCherry+ targeted PV+ cells contacting NeuN+ somata. We observed that the percentage of PV+ cells infected in the area was linearly correlated with the percentage

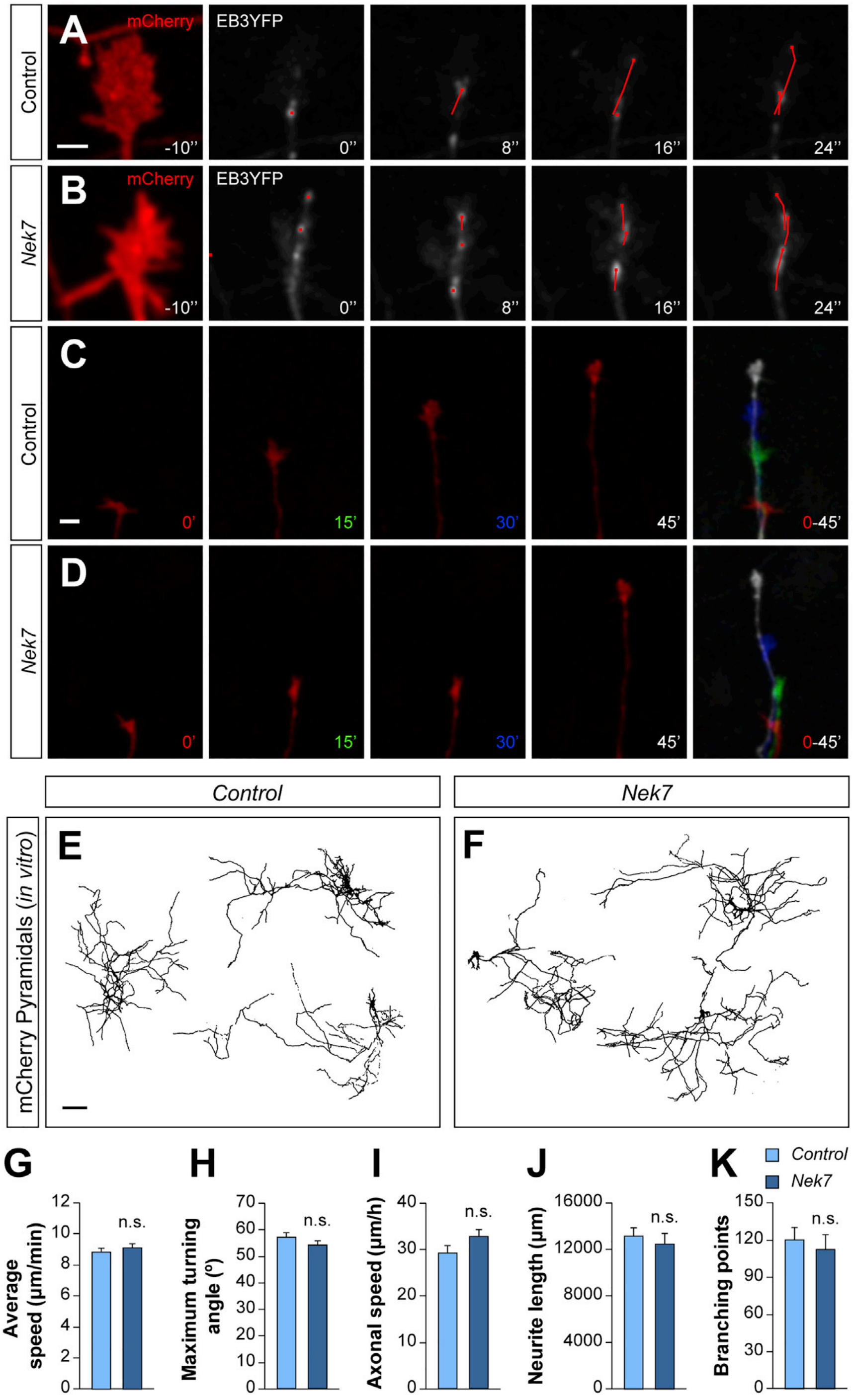

Figure 5. *Nek7* Overexpression in Pyramids Does Not Alter Their Axonal Development

(A and B) Confocal Z projection frames from *NexCre* growth cones expressing mCherry (red, before the time-lapse), EB3-YFP (gray, time-lapse), and m*Nek7* in the *Nek7* condition. Control (A) and m*Nek7* (B). The path of EB3-YFP comets is tracked with a red line.

(C and D) Confocal Z projection frames from *NexCre* growth cones. Control (C) and m*NEK7* (D). The last frame in the sequence shows superimposed images of the frames t = 0 min (red), t = 15 min (green), t = 30 min (blue), and t = 45 min (white).

(E and F) Confocal Z projections of *NexCre* interneurons expressing mCherry. Control (E) and m*Nek7* (F). The cells were automatically reconstructed and masked in black at 8 DIV.

(G) Average speed of EB3 comets comparing control pyramids (A; n = 53 growth cones) and *Nek7*-expressing pyramids (B; n = 54 growth cones) from three independent cultures.

(H and I) Average of growth cone maximum turning angle (H) and axonal speed (I) from control pyramids (C; n = 73 growth cones) and *Nek7*-expressing pyramids (D; n = 74 growth cones) from three independent cultures.

(J and K) Neurite length (J) and branching points (K) comparing control pyramids (E; n = 44 cells) and *Nek7*-expressing pyramids (F; n = 38 cells) transfected neurons from three independent cultures.

One-way ANOVA (G and I) and Mann-Whitney test (H, J, and K). n.s., not significant. Data are represented as mean ± SEM. Scale bars represent 2 µm (A and B), 5 µm (C and D), and 200 µm (E and F).

of SYT2+/mCherry+ boutons (Figure S6H), and we therefore normalized the number of mCherry+ boutons on each pyramidal cell soma to the percentage of infected PV+ cells in the area to compare across samples. We found a consistent decrease in the percentage of SYT2+/mCherry+ boutons in *Nek7*-knockdown interneurons compared with controls (Figures 7E–7H). The synaptic phenotype was consistently restored by m*Nek7* (Figures 7G, 7H, S6I, and S6J). Taken together, these results reinforce the notion that *Nek7* depletion from PV+ interneurons causes defects in axonal arborization and altered synaptic connectivity.

DISCUSSION

During postnatal development, pyramidal cells and interneurons undergo extraordinarily divergent transformations in their morphologies. Pyramidal cell dendrites acquire a very stereotyped polarity and complexity, and pyramidal cell axons follow a near-linear path toward their long-range targets (Spruston, 2008; Stepanyants et al., 2004). In contrast, interneuron dendrites are less elaborated than those of pyramidal cells, but their axonal arbors have a remarkably tortuous outgrowth to form dense networks of local connections (Jiang et al., 2015; Stepanyants et al., 2004). Although these morphological differences are very evident, little is known about the molecular mechanisms underlying their divergent development. In this study, we addressed this question by carrying out a high-throughput

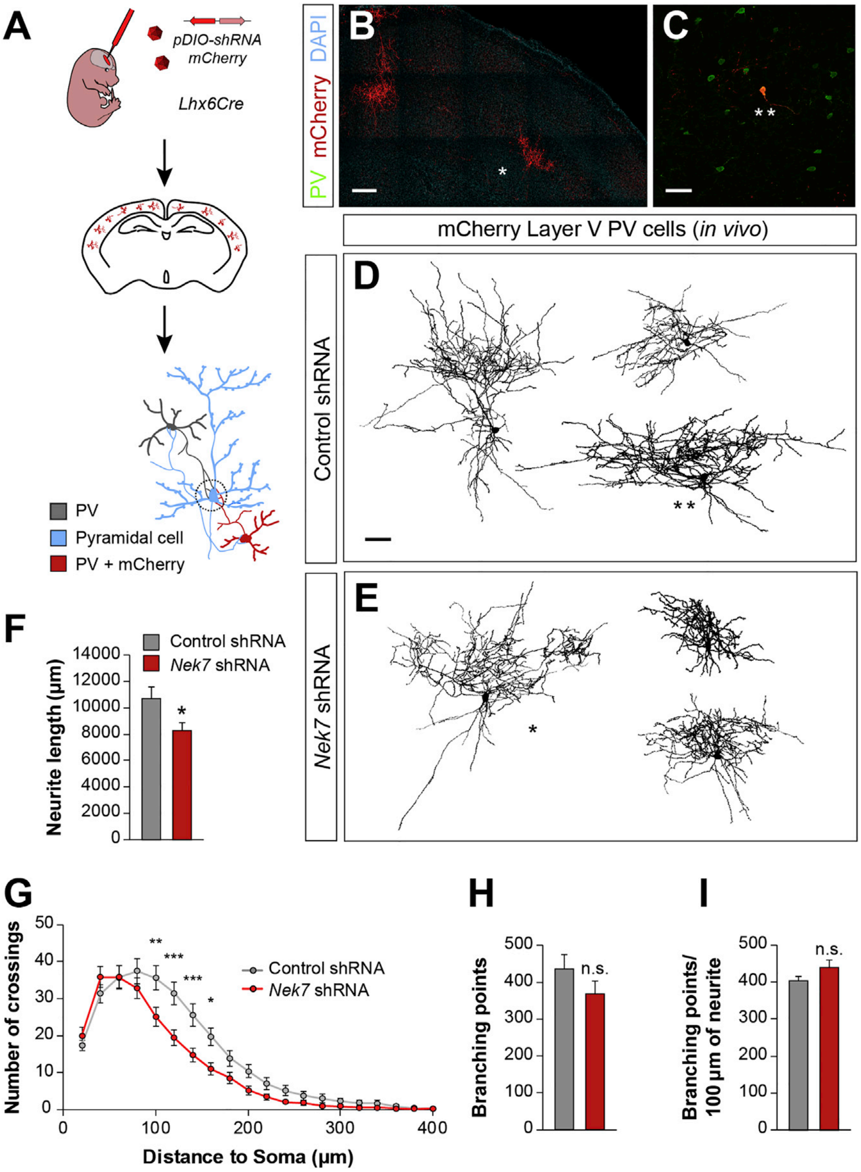

Figure 6. Loss of NEK7 Causes Abnormal PV+ Neuronal Morphology *In Vivo*

(A) Schematic of experimental design. Cre-dependent AAVs expressing shRNA and the fluorescent marker mCherry were injected in E15.5 *Lhx6Cre* mice *in vivo*.

(B and C) Targeted mCherry+ interneurons (B) that were PV+ (C) were selected for the analysis.

(D and E) Confocal Z projections of PV+ interneurons expressing control shRNA (D) and *Nek7* shRNA (E) reported by mCherry. The cells were automatically reconstructed and masked in black from layer V of somatosensory cortex at P21.

(F–I) Average of total neurite length (F), Sholl analysis (G), total branching points (H), and branching points per 100 µm of neurite (I) of control shRNA (n = 30 PV+ neurons, from 10 mice) and *Nek7* shRNA (n = 27 PV+ neurons, from 8 mice) infected neurons. One-way ANOVA (F, H, and I) and two-way ANOVA with Bonferroni correction (G).

*$p < 0.05$, **$p < 0.01$, and ***$p < 0.001$; n.s., not significant. Data are represented as mean ± SEM. Scale bars represent 200 µm (B) and 50 µm (C–E). See also Figure S6.

genetic screen to identify differentially expressed genes during the wiring of GABAergic interneurons, highlighting the kinase NEK7 as a regulator of PV+ interneuron connectivity. Loss of NEK7 from putative PV+ cells alters the dynamics of both microtubules and growth cones and causes an abnormal axonal arborization and a reduction in the number of PV+ synaptic inputs impinging onto pyramidal cells. Altogether, our results unveil a specific molecular mechanism by which the microtubule-associated kinase NEK7 instructs the wiring of PV+ interneurons.

Several transcriptome analyses in the mouse cerebral cortex have previously defined the molecular profiles of mature glutamatergic pyramidal cells and GABAergic interneurons (Sugino et al., 2006; Tasic et al., 2016). However, information is missing about the developmental emergence of the molecular programs that define the profound morphological and functional characteristics that distinguish pyramidal cells and interneurons. An elegant forward genetic screening *in vitro*, aiming to identify differentially expressed genes between glutamatergic and GABAergic neurons during synaptogenesis, described mostly common mediators for both types of synapses rather than specific regulators of each type (Paradis et al., 2007). Our study unveils a set of 133 genes enriched in interneurons during axon development and synapse formation *in vivo*. The validation of NEK7, one of the most prominent candidates, as a regulator of interneuron axon development supports our approach to identify molecules involved in GABAergic wiring, thus providing a solid database to further expand this analysis to other candidate genes.

NEK7 has been previously involved in the formation of microtubule-based structures and the regulation of microtubule dynamics in cell lines and fibroblasts (Cohen et al., 2013; Kim et al., 2007; O'Regan and Fry, 2009; Salem et al., 2010; Yissachar et al., 2006). Specifically, loss of NEK7 decreases the growth and catastrophe rates of the microtubules in these cells (Cohen et al., 2013). In the cerebral cortex, *Nek7* transcripts are expressed mainly in PV+ interneurons, which are characterized by very complex and unique axonal arbors (Dumitriu et al., 2007; Jiang et al., 2015; Tremblay et al., 2016). As in non-neuronal cells, NEK7 also regulates microtubule dynamics in PV+ interneurons and, as a consequence, axon development. Interestingly, we have found that not only is the expression of *Nek7* specific to interneurons in the cerebral cortex, but its

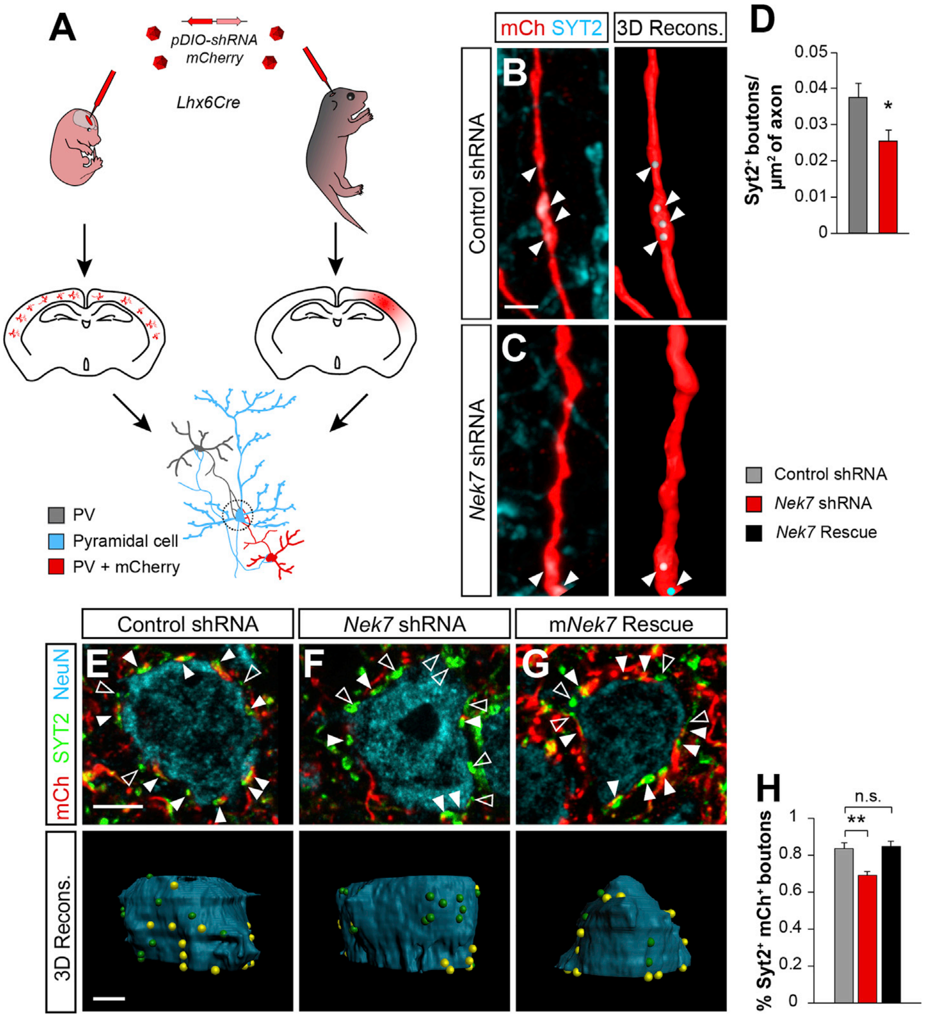

Figure 7. *Nek7* Knockdown Decreases PV+ Interneuron Outputs

(A) Schematic of the experimental design. Cre-dependent virus expressing shRNA and the fluorescent marker *mCherry* were injected in E15.5 (left) and P3 (right) *Lhx6Cre* mice *in vivo*. Boutons co-stained with SYT2 and mCherry were quantified either inside PV+ arbors or onto NeuN+ somata.

(B and C) Confocal Z projections and Imaris 3D reconstructions showing control shRNA (B) and *Nek7* shRNA (C) infected PV+ cells expressing mCherry (red) in axons containing SYT2+ boutons (blue).

(D) SYT2+ bouton density per unit area of neurite comparing control shRNA (n = 24 PV+ neurons from four mice) and *Nek7* shRNA (n = 22 PV+ neurons from six mice). Student's t test.

(E–G) Confocal images and 3D Imaris reconstructions showing mCherry+ (red), SYT2+ (green) synaptic boutons from infected PV+ cells expressing control shRNA (E), *Nek7* shRNA (F), and *Nek7* shRNA with m*Nek7* for rescue (G) contacting pyramidal cells NeuN+ (blue). SYT2+ mCherry+ (filled arrowheads, yellow spheres), SYT2+ mCherry− (open arrowheads, green spheres).

(H) Percentage of SYT2+ mCherry+ somatic boutons contacting the pyramidal cells normalized to the percentage of PV+ cells infected in the area, comparing shRNA (n = 182 PV+ neurons from seven mice), *Nek7* shRNA (n = 200 pyramidal cells from six mice), and m*Nek7* (n = 109 pyramidal cells from four mice). Kruskal-Wallis test, pairwise comparisons.

$^*p < 0.05$ and $^{**}p < 0.01$; n.s., not significant. Data are represented as mean ± SEM. Scale bars represent 2 µm (B and C) and 5 µm (E–G). See also Figure S6.

exogenous overexpression in pyramidal cells is not sufficient to change microtubule dynamics and axonal development in this population (Figure 5). This suggests that NEK7 is part of a broader molecular program present in interneurons but not in pyramidal cells. Further studies will be required to elucidate the molecular mechanisms by which NEK7 mediates microtubule dynamics in PV+ interneurons.

The motility and growth of axons in response to extracellular cues require precise interactions between the actin and microtubule cytoskeletons (Conde and Cáceres, 2009; Geraldo and Gordon-Weeks, 2009; Kalil and Dent, 2014). Although actin polymerization is the driving force of axon protrusion, it is only when microtubules invade the filopodia that those become axonal branches. Thus, changes in microtubule dynamics can alter the development of the axon (Conde and Cáceres, 2009). Consistent with this idea, we found that *Nek7* knockdown impairs microtubule dynamics, which leads to axons with altered growth cone steering.

Intriguingly, although microtubule dynamics and axonal behavior depend on NEK7 kinase activity, the arborization phenotype seems to be kinase independent. It is plausible that higher levels of the protein expressed by transfection together with its residual kinase activity (O'Regan and Fry, 2009) may not be sufficient to increase microtubule dynamics and axonal growth but could compensate the morphological phenotype after several DIV. Alternatively, NEK7 may contribute to the final formation of the axonal arbor through a molecular mechanism independent of its kinase activity, as kinase-independent interactions of NEK7 have been previously described (He et al., 2016). Future experiments targeting the dead-kinase domain at the endogenous locus will shed light on this interesting phenomenon.

We observed a decrease in the synaptic connections of PV+ interneurons onto pyramidal cells that might be independent of the length of the axons. Supporting this idea, synaptic structure and function are also sensitive to changes in microtubule dynamics (Roos et al., 2000). It is also plausible that shorter and misrouted axons with less elaborated arbors, as observed in NEK7-deficient PV+ cells, may have fewer opportunities to reach all the appropriated pyramidal cell targets to form synapses.

The precise function of NEK7 in microtubule dynamics and axonal wiring, together with its specific expression in PV+ cells, suggests that interneurons use specific mechanisms for axon growth and synaptic targeting that are not present in other cortical cells, including pyramidal cells. Compared with the axons of their neighboring pyramidal cells, cortical interneuron axonal paths are more tortuous and form a greater number of crossings with their synaptic targets (Huang et al., 2007; Stepanyants et al., 2004; Wierenga et al., 2008). An attractive explanation for these observations is that the assembly of GABAergic synapses might be mainly the outcome of the exploratory behavior of axons driven by a unique molecular program that is absent in pyramidal cells. It is tempting to speculate that a collection of proteins such as NEK7 confers unique behaviors to the PV+ axons that allow them to increase their axonal arbor complexity and their efficiency to establish specific connections with the highest possible number of neighbor pyramidal cells.

EXPERIMENTAL PROCEDURES

Mice

Animal procedures were approved by ethical committees (IN-CSIC and King's College London) and conducted in accordance with Spanish and European Union regulations and Home Office personal and project licenses under the UK Animals (Scientific Procedures) 1986 Act. All the experiments *in vivo* were performed in males, and gender was not distinguishable for primary cultures.

FACS, Microarrays, and qPCR

Neurons expressing GFP were isolated by FACS (FACSAria II; BD Biosciences) and snap-frozen in liquid nitrogen to be kept at −80°C. Total RNA for microarray analysis and qPCR was extracted and purified using the RNeasy Micro extraction kit (QIAGEN) and Tri-reagent extraction method (Sigma-Aldrich) following the manufacturers' instructions and hybridized to Mouse Gene 1.0 ST Array (Affymetrix).

Microarray fluorescence intensity data were analyzed using the R package BioConductor (Ihaka and Gentleman, 1996). Normalization was carried out with the algorithm Robust Multiarray Average (RMA). Finally, statistical significance for differential expression between the samples (IN P10 versus IN P0, PyrP12 versus Pyr P0, and IN P10 versus Pyr P12) was measured using significance analysis of microarrays (SAM), selecting genes with a false discovery rate (FDR) lower than 0.05 and a fold change greater than 2. Subsequently, these lists were compared among them to obtain genes specifically expressed in IN P10. The selected candidate was confirmed by qPCR.

DNA Constructs, Viral Production, and Viral Injections

All shRNAs, including *Nek7* shRNA, were designed using Block-iT (Thermo Fisher Scientific) against the open reading frame (ORF) sequence adding EcoRI and *AvrII* enzymatic restriction sites at the 5′ and 3′ ends, respectively. Control shRNAs were designed against *gfp* and *lacZ* gene sequences, which are absent in the mouse. shRNAs were synthetized as single-stranded DNA (ssDNA) and annealed to their complementary strand before being cloned into the *pDIO-shRNA-mCherry* vector. The engineering of the *pDIO-shRNA-mCherry* vector has been described previously (Favuzzi et al., 2017).

To generate the *shRNA*-resistant NEK7 full-length (m*Nek7*), we introduced synonymous mutations in the shRNA targeting sequence (5′GAGAGAA CCGTTTGGAAATAC-3′) and added 3 × FLAG sequences as a tag at the C-terminal end of the coding sequence. The synthetic double-stranded DNA (dsDNA) sequence (Strings-GeneArt Gene Synthesis Service; Thermo Fisher Scientific) was A′-tailed and cloned into the *pGEMT*-Easy Vector (Promega) and sub-cloned, after sequence verification, into the vector *pDIO-Cheta-TdTomato* (37755; Addgene). The CDS *Cheta-TdTomato* sequence was substituted by m*Nek7* using *AscI* and *NheI*.

The coding sequence for EB3 was PCR-amplified from a plasmid kindly provided by Dr. Niels Galjart (Erasmus MC) and sub-cloned into *pILES-YFP* vector to produce the *pILES-EB3-YFP* as previously described (Geraldo et al., 2008).

To obtain isolated infected cells for morphological reconstructions, we injected 1 μL of AAVs diluted 1:30, in PBS with FastGreen 0.5%, into the telencephalic lateral ventricle of E15.5 *Lhx6Cre* embryos. To increase the yield of infection for the analysis of PV+ wiring in pyramidal cells, P3–P4 mice were anesthetized with isoflurane and placed in a stereotaxic frame. For detailed procedures, see Supplemental Experimental Procedures.

Primary Cultures and Transfection

Cortices from *Nkx2-1Cre* or *NexCre* embryos at E17.5–E18.5 were dissected, and cells were dissociated with trypsin (1 mg/mL, 15 min at 37°C) followed by gentle mechanical trituration as described previously (Rico et al., 2004). For detailed procedures, see Supplemental Experimental Procedures.

Immunohistochemistry, Immunocytochemistry, and *In Situ* Hybridization

Mice were anesthetized and perfused with PBS followed by 4% paraformaldehyde (PFA) in PBS. Similarly, cortical cultures were fixed in 4% PFA and 4% sucrose diluted in culture media during 20 min at 12 DIV (interneurons) or 8 DIV (pyramidal cells) for immunocytochemistry. For detailed procedures, see Supplemental Experimental Procedures.

For dual-color fluorescence *in situ* hybridization combined with immunohistochemistry, mice were perfused with 4% PFA in PBS and the dissected brains fixed overnight in the same solution. Brains were then cut at 30 μm, and free-floating coronal sections were subsequently hybridized with digoxigenin-labeled probes and antibodies as described previously (Favuzzi et al., 2017). Probe sequence against *Nek7* was obtained from the Allen Brain Atlas database (Lein et al., 2007) and amplified using the following primers: RP_050725_01_H03, 5′-CGGAGGAGCTACGACAGC-3′, 5′-TGACTATCACG CCAGGCA-3′. Nitro blue tetrazolium (NBT)/5-bromo-4-chloro-3-indolyl phosphate (BCIP) colorimetric *in situ* hybridization was performed as described previously (Flames et al., 2007).

Image Acquisition and Analysis

Images were acquired at 1,024 × 1,024 pixel resolution, 8-bit depth, and 400 Hz in an inverted Leica TCS-SP8 confocal microscope, blind to the experimental condition. Image analysis was performed using Imaris 8.1.2 (Bitplane) after applying background subtraction and Gaussian filtering with this software. Time-lapse experiments were carried out in an incubation chamber to keep

the cultures at 37°C and 4% CO_2, and recordings were done at 7 DIV using a resonant scanner (15%) and a hybrid detector (8,000 Hz). For microtubule dynamics quantification, confocal image stacks (100×, 1.44 NA objective, digital zoom 2.2, 0.2 μm step size) were taken at 2 s intervals during 2 min and pixel resolution was reduced to 512 × 512 to increase the scanning speed. For growth cone dynamics analysis, confocal image stacks (20×, 0.5 NA objective, digital zoom 4.0, 1 μm step size) were taken at 2.5 min intervals during 1 hr.

For *in vitro* morphological reconstructions, neurons were imaged with a 20× objective (0.5 NA, digital zoom 0.75, 600 Hz, 1 μm z-step size). Conversely, *in vivo* morphologies were imaged with a 63× objective (1.4 NA, digital zoom 0.75 at 400 Hz, 0.5 μm z-step size). Although we could not confirm PV identity *in vitro* because of the early time point of the primary cultures that benefit the trace of isolated axons, we confirmed that the neuron was positive for PV *in vivo*, before any morphological reconstruction.

For synaptic bouton quantifications, the same 100× objective was used to image 10 μm z stacks from the surface of the slice. For the synapses in isolated neurons, once PV+ cells were identified, random regions of the neurite arbor targeted with a Cre-dependent *lacZ* shRNA or *Nek7* shRNA reported by mCherry were imaged, and a surface was generated with the mCherry channel.

Single plane confocal images were acquired with a 40× objective (1.3 NA, digital zoom 0.75) and cell density and colocalization quantified with the Cell Counter plug-in for ImageJ (NIH). Cells were quantified independently in the different channels and then compared to identify co-expression of two markers. The same experimental procedure was used for the *in situ* colocalizations of *Nek7*.

Statistical Analysis

Statistical analysis was performed using SPSS software (IBM). To obtain unbiased data, experimental mice from the different conditions were processed together, and quantifications were performed blind to the experimental condition. Data were analyzed with parametric tests, Student's t test or ANOVA, when datasets met assumptions of normality (Kolmogorov-Smirnov test) and homoscedasticity (Levene test). In ANOVA, this was followed by Bonferroni post hoc analysis for comparisons of multiple conditions. Non-parametric tests for independent groups were applied when normality was not met (Mann-Whitney, Kruskal-Wallis). For the Kruskal-Wallis test, the Dunn-Bonferroni post hoc test was used for comparison of multiple samples. Sholl analysis data were analyzed using two-way ANOVA with Bonferroni correction. Finally, the Mantel-Haenszel χ^2 test was used to compare the population distributions. Data are expressed as mean ± SEM. Population distributions are expressed as total cell percentages. Differences were considered statistically significant at $p < 0.05$.

DATA AND SOFTWARE AVAILABILITY

The accession number for the microarray data reported in this paper is GEO: GSE115948.

SUPPLEMENTAL INFORMATION

Supplemental Information includes Supplemental Experimental Procedures, seven figures, one table, and six videos and can be found with this article online at https://doi.org/10.1016/j.celrep.2018.06.115.

ACKNOWLEDGMENTS

We are thankful to D. Baeza and N. Carvajal for lab support, I. Andrew for mouse management, L. Doglio for her technical assistance, S.A. Anderson (University of Pennsylvania) for *Nkx2-1Cre* mice, N. Kessaris (University College London) for *Lhx6Cre* mice, S. Goebbels and K. A. Nave (Max Planck Institute of Experimental Medicine) for Nex^{Cre} mice, P. Gordon-Weeks (King's College London) for the *pILES-EB3-YFP* vector, Fazal Oozeer for his technical help on cloning *Nek7*-NeonGreen and *Nek7* KD, and R. Seeamber for helping test the shRNA system. We are grateful to O. Marín, P. Gordon-Weeks, C. Bernard, and L. Lim for critical reading of the manuscript and members of the Rico and Marín laboratories for stimulating discussions and ideas. This work was supported by grants from the Spanish Government (SAF2010-21723) and European Research Council (ERC-2012-StG 310021) to B.R. A.J.H. was a recipient of a Formación de Personal Investigador (FPI) fellowship from the Spanish Government (SAF2010-21723) and was subsequently supported by King's College London funds. B.R. is Wellcome Trust investigator.

AUTHOR CONTRIBUTIONS

B.R. and A.J.H. conceived the project. R.D. designed the plasmid backbones. A.J.H. performed all the experiments and analyzed the data. A.J.H. and B.R. interpreted the data and wrote the manuscript.

DECLARATION OF INTERESTS

The authors declare no competing interests. R.D. is currently working for Reneuron.

Received: October 10, 2017
Revised: May 29, 2018
Accepted: June 27, 2018
Published: July 31, 2018

REFERENCES

Alsina, B., Vu, T., and Cohen-Cory, S. (2001). Visualizing synapse formation in arborizing optic axons in vivo: dynamics and modulation by BDNF. Nat. Neurosci. *4*, 1093–1101.

Ascoli, G.A., Alonso-Nanclares, L., Anderson, S.A., Barrionuevo, G., Benavides-Piccione, R., Burkhalter, A., Buzsáki, G., Cauli, B., Defelipe, J., Fairén, A., et al.; Petilla Interneuron Nomenclature Group (2008). Petilla terminology: nomenclature of features of GABAergic interneurons of the cerebral cortex. Nat. Rev. Neurosci. *9*, 557–568.

Ben-Ari, Y. (2002). Excitatory actions of gaba during development: the nature of the nurture. Nat. Rev. Neurosci. *3*, 728–739.

Cembrowski, M.S., Wang, L., Sugino, K., Shields, B.C., and Spruston, N. (2016). Hipposeq: a comprehensive RNA-seq database of gene expression in hippocampal principal neurons. eLife *5*, e14997.

Cohen, S., Aizer, A., Shav-Tal, Y., Yanai, A., and Motro, B. (2013). Nek7 kinase accelerates microtubule dynamic instability. Biochim. Biophys. Acta *1833*, 1104–1113.

Conde, C., and Cáceres, A. (2009). Microtubule assembly, organization and dynamics in axons and dendrites. Nat. Rev. Neurosci. *10*, 319–332.

Custo Greig, L.F., Woodworth, M.B., Galazo, M.J., Padmanabhan, H., and Macklis, J.D. (2013). Molecular logic of neocortical projection neuron specification, development and diversity. Nat. Rev. Neurosci. *14*, 755–769.

De Felipe, J., Marco, P., Fairén, A., and Jones, E.G. (1997). Inhibitory synaptogenesis in mouse somatosensory cortex. Cereb. Cortex *7*, 619–634.

de Wit, J., and Ghosh, A. (2016). Specification of synaptic connectivity by cell surface interactions. Nat. Rev. Neurosci. *17*, 22–35.

DeFelipe, J., Alonso-Nanclares, L., and Arellano, J.I. (2002). Microstructure of the neocortex: comparative aspects. J. Neurocytol. *31*, 299–316.

Dent, E.W., and Kalil, K. (2001). Axon branching requires interactions between dynamic microtubules and actin filaments. J. Neurosci. *21*, 9757–9769.

Dent, E.W., Gupton, S.L., and Gertler, F.B. (2011). The growth cone cytoskeleton in axon outgrowth and guidance. Cold Spring Harb. Perspect. Biol. *3*, 3.

Dumitriu, D., Cossart, R., Huang, J., and Yuste, R. (2007). Correlation between axonal morphologies and synaptic input kinetics of interneurons from mouse visual cortex. Cereb. Cortex *17*, 81–91.

Favuzzi, E., Marques-Smith, A., Deogracias, R., Winterflood, C.M., Sánchez-Aguilera, A., Mantoan, L., Maeso, P., Fernandes, C., Ewers, H., and Rico, B. (2017). Activity-dependent gating of parvalbumin interneuron function by the perineuronal net protein brevican. Neuron *95*, 639–655.e10.

Fishell, G., and Rudy, B. (2011). Mechanisms of inhibition within the telencephalon: "where the wild things are". Annu. Rev. Neurosci. *34*, 535–567.

Flames, N., Pla, R., Gelman, D.M., Rubenstein, J.L.R., Puelles, L., and Marín, O. (2007). Delineation of multiple subpallial progenitor domains by the combinatorial expression of transcriptional codes. J. Neurosci. *27*, 9682–9695.

Fry, A.M., O'Regan, L., Sabir, S.R., and Bayliss, R. (2012). Cell cycle regulation by the NEK family of protein kinases. J. Cell Sci. *125*, 4423–4433.

Geraldo, S., and Gordon-Weeks, P.R. (2009). Cytoskeletal dynamics in growth-cone steering. J. Cell Sci. *122*, 3595–3604.

Geraldo, S., Khanzada, U.K., Parsons, M., Chilton, J.K., and Gordon-Weeks, P.R. (2008). Targeting of the F-actin-binding protein drebrin by the microtubule plus-tip protein EB3 is required for neuritogenesis. Nat. Cell Biol. *10*, 1181–1189.

Gordon-Weeks, P.R. (2004). Microtubules and growth cone function. J. Neurobiol. *58*, 70–83.

Haq, T., Richards, M.W., Burgess, S.G., Gallego, P., Yeoh, S., O'Regan, L., Reverter, D., Roig, J., Fry, A.M., and Bayliss, R. (2015). Mechanistic basis of Nek7 activation through Nek9 binding and induced dimerization. Nat. Commun. *6*, 8771.

He, Y., Zeng, M.Y., Yang, D., Motro, B., and Núñez, G. (2016). NEK7 is an essential mediator of NLRP3 activation downstream of potassium efflux. Nature *530*, 354–357.

Hoogenraad, C.C., and Bradke, F. (2009). Control of neuronal polarity and plasticity–a renaissance for microtubules? Trends Cell Biol. *19*, 669–676.

Huang, Z.J., Di Cristo, G., and Ango, F. (2007). Development of GABA innervation in the cerebral and cerebellar cortices. Nat. Rev. Neurosci. *8*, 673–686.

Ihaka, R., and Gentleman, R. (1996). R: a language for data analysis and graphics. J. Comput. Graph. Stat. *5*, 299.

Jiang, X., Shen, S., Cadwell, C.R., Berens, P., Sinz, F., Ecker, A.S., Patel, S., and Tolias, A.S. (2015). Principles of connectivity among morphologically defined cell types in adult neocortex. Science *350*, aac9462.

Kalil, K., and Dent, E.W. (2014). Branch management: mechanisms of axon branching in the developing vertebrate CNS. Nat. Rev. Neurosci. *15*, 7–18.

Kim, S., Lee, K., and Rhee, K. (2007). NEK7 is a centrosomal kinase critical for microtubule nucleation. Biochem. Biophys. Res. Commun. *360*, 56–62.

Kolodkin, A.L., and Tessier-Lavigne, M. (2011). Mechanisms and molecules of neuronal wiring: a primer. Cold Spring Harb. Perspect. Biol. *3*, 3.

Kryuchkova-Mostacci, N., and Robinson-Rechavi, M. (2017). A benchmark of gene expression tissue-specificity metrics. Brief. Bioinform. *18*, 205–214.

Larsen, D.D., and Callaway, E.M. (2006). Development of layer-specific axonal arborizations in mouse primary somatosensory cortex. J. Comp. Neurol. *494*, 398–414.

Lein, E.S., Hawrylycz, M.J., Ao, N., Ayres, M., Bensinger, A., Bernard, A., Boe, A.F., Boguski, M.S., Brockway, K.S., Byrnes, E.J., et al. (2007). Genome-wide atlas of gene expression in the adult mouse brain. Nature *445*, 168–176.

Marín, O. (2012). Interneuron dysfunction in psychiatric disorders. Nat. Rev. Neurosci. *13*, 107–120.

Maroof, A.M., Brown, K., Shi, S.-H., Studer, L., and Anderson, S.A. (2010). Prospective isolation of cortical interneuron precursors from mouse embryonic stem cells. J. Neurosci. *30*, 4667–4675.

McAllister, A.K. (2007). Dynamic aspects of CNS synapse formation. Annu. Rev. Neurosci. *30*, 425–450.

Meyer, M.P., and Smith, S.J. (2006). Evidence from in vivo imaging that synaptogenesis guides the growth and branching of axonal arbors by two distinct mechanisms. J. Neurosci. *26*, 3604–3614.

Morris, N.R. (1976). Nucleosome structure in Aspergillus nidulans. Cell *8*, 357–363.

Nakajima, M., Görlich, A., and Heintz, N. (2014). Oxytocin modulates female sociosexual behavior through a specific class of prefrontal cortical interneurons. Cell *159*, 295–305.

Navarro, A.I., and Rico, B. (2014). Focal adhesion kinase function in neuronal development. Curr. Opin. Neurobiol. *27*, 89–95.

O'Connell, M.J., Krien, M.J.E., and Hunter, T. (2003). Never say never. The NIMA-related protein kinases in mitotic control. Trends Cell Biol. *13*, 221–228.

O'Regan, L., and Fry, A.M. (2009). The Nek6 and Nek7 protein kinases are required for robust mitotic spindle formation and cytokinesis. Mol. Cell. Biol. *29*, 3975–3990.

Paradis, S., Harrar, D.B., Lin, Y., Koon, A.C., Hauser, J.L., Griffith, E.C., Zhu, L., Brass, L.F., Chen, C., and Greenberg, M.E. (2007). An RNAi-based approach identifies molecules required for glutamatergic and GABAergic synapse development. Neuron *53*, 217–232.

Rico, B., Beggs, H.E., Schahin-Reed, D., Kimes, N., Schmidt, A., and Reichardt, L.F. (2004). Control of axonal branching and synapse formation by focal adhesion kinase. Nat. Neurosci. *7*, 1059–1069.

Roos, J., Hummel, T., Ng, N., Klämbt, C., and Davis, G.W. (2000). Drosophila Futsch regulates synaptic microtubule organization and is necessary for synaptic growth. Neuron *26*, 371–382.

Ruthazer, E.S., Li, J., and Cline, H.T. (2006). Stabilization of axon branch dynamics by synaptic maturation. J. Neurosci. *26*, 3594–3603.

Salem, H., Rachmin, I., Yissachar, N., Cohen, S., Amiel, A., Haffner, R., Lavi, L., and Motro, B. (2010). Nek7 kinase targeting leads to early mortality, cytokinesis disturbance and polyploidy. Oncogene *29*, 4046–4057.

Shen, K., and Scheiffele, P. (2010). Genetics and cell biology of building specific synaptic connectivity. Annu. Rev. Neurosci. *33*, 473–507.

Sommeijer, J.-P., and Levelt, C.N. (2012). Synaptotagmin-2 is a reliable marker for parvalbumin positive inhibitory boutons in the mouse visual cortex. PLoS ONE *7*, e35323.

Spruston, N. (2008). Pyramidal neurons: dendritic structure and synaptic integration. Nat. Rev. Neurosci. *9*, 206–221.

Stepanova, T., Slemmer, J., Hoogenraad, C.C., Lansbergen, G., Dortland, B., De Zeeuw, C.I., Grosveld, F., van Cappellen, G., Akhmanova, A., and Galjart, N. (2003). Visualization of microtubule growth in cultured neurons via the use of EB3-GFP (end-binding protein 3-green fluorescent protein). J. Neurosci. *23*, 2655–2664.

Stepanyants, A., Tamás, G., and Chklovskii, D.B. (2004). Class-specific features of neuronal wiring. Neuron *43*, 251–259.

Sugino, K., Hempel, C.M., Miller, M.N., Hattox, A.M., Shapiro, P., Wu, C., Huang, Z.J., and Nelson, S.B. (2006). Molecular taxonomy of major neuronal classes in the adult mouse forebrain. Nat. Neurosci. *9*, 99–107.

Tasic, B., Menon, V., Nguyen, T.N., Kim, T.K., Jarsky, T., Yao, Z., Levi, B., Gray, L.T., Sorensen, S.A., Dolbeare, T., et al. (2016). Adult mouse cortical cell taxonomy revealed by single cell transcriptomics. Nat. Neurosci. *19*, 335–346.

Tremblay, R., Lee, S., and Rudy, B. (2016). Gabaergic interneurons in the neocortex: from cellular properties to circuits. Neuron *91*, 260–292.

Wierenga, C.J., Becker, N., and Bonhoeffer, T. (2008). GABAergic synapses are formed without the involvement of dendritic protrusions. Nat. Neurosci. *11*, 1044–1052.

Yissachar, N., Salem, H., Tennenbaum, T., and Motro, B. (2006). Nek7 kinase is enriched at the centrosome, and is required for proper spindle assembly and mitotic progression. FEBS Lett. *580*, 6489–6495.

Zeisel, A., Muñoz-Manchado, A.B., Codeluppi, S., Lönnerberg, P., La Manno, G., Juréus, A., Marques, S., Munguba, H., He, L., Betsholtz, C., et al. (2015). Brain structure. Cell types in the mouse cortex and hippocampus revealed by single-cell RNA-seq. Science *347*, 1138–1142.

Cell Reports, Volume *24*

Supplemental Information

The Microtubule Regulator NEK7 Coordinates the Wiring of Cortical Parvalbumin Interneurons

Antonio Jesús Hinojosa, Rubén Deogracias, and Beatriz Rico

SUPPLEMENTAL INFORMATION

SUPPLEMENTAL FIGURES

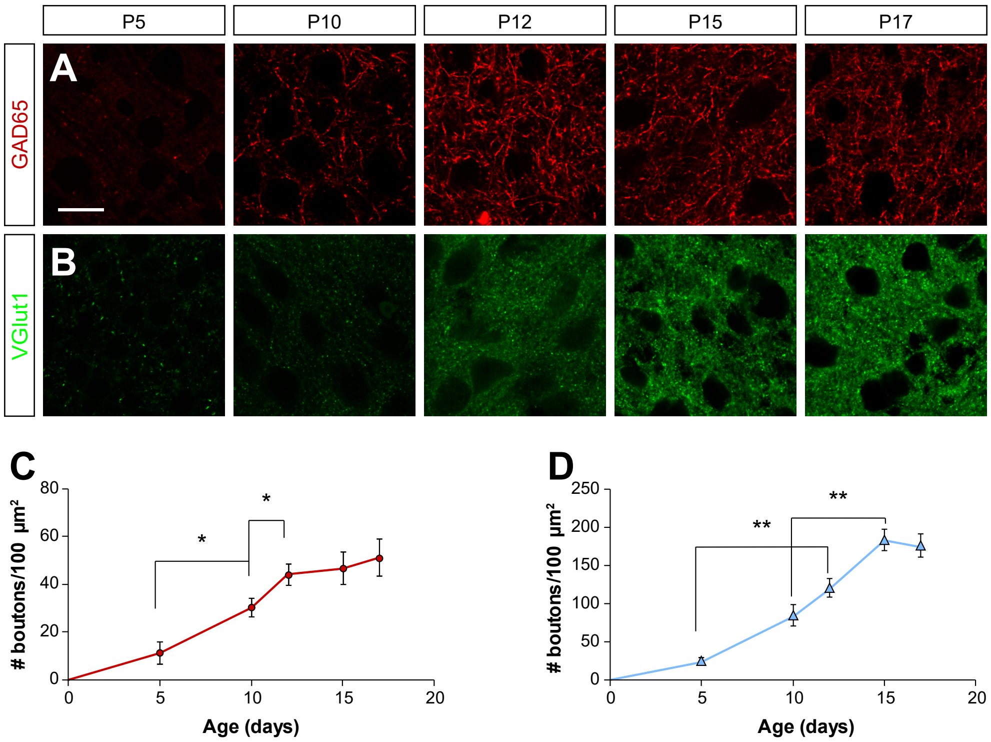

Figure S1. Time course of the development of GABAergic and glutamatergic inputs. Related to Figure 1. (A and B) Single confocal images showing GAD65 (A, red) and VGlut1 (B, green) immunostaining during postnatal development in layer II/III of wild-type somatosensory cortex.

(C and D) GAD65 (C) and VGlut1 (D) bouton density (n = 3 mouse per age).

One-way ANOVA, Post hoc Bonferroni, * $p < 0.05$, ** $p < 0.01$. Data are represented as mean ± SEM. Scale bar represents 10 µm.

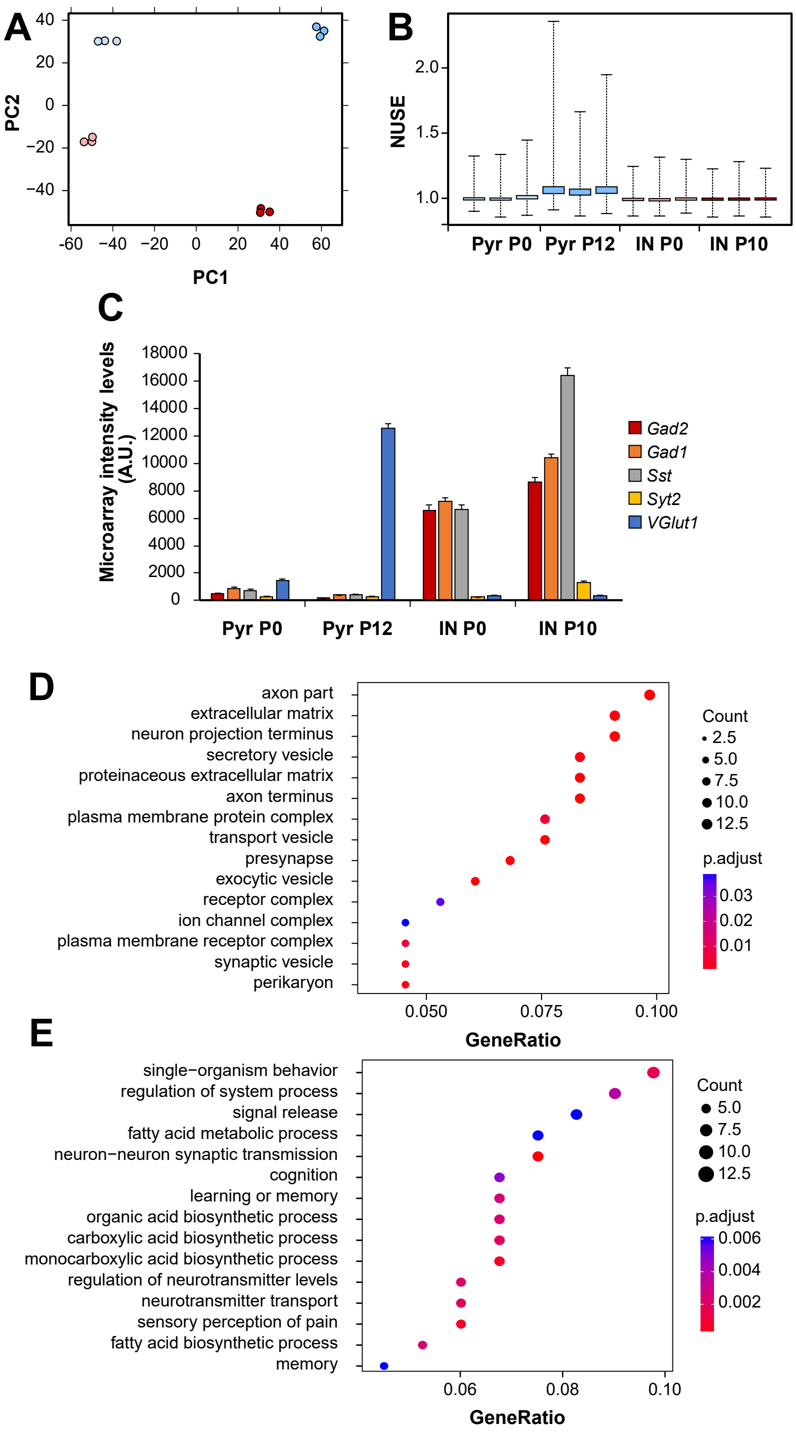

Figure S2. Quality control experiments for microarrays and gene ontology (GO). Related to Figure 1.
(A) Scatterplot showing the distribution of the different microarray condition replicates along the first two principal components.
(B) Boxplot showing the error of the median intensity values for each probe-set normalized to 1 (NUSE, Normalized Unscaled Standard Error).
(C) Microarray intensity levels of cell population specific markers [Interneurons: Gad1 (GAD67), Gad2 (GAD65), Sst and Syt2; pyramidal cells: Slc17a7 (VGlut1)] (A.U.: arbitrary units).
(D and E) Representative categories of the GO analysis. Ratio of genes from the filtered gene set belonging to the most representative categories of the cellular (D) and biological (E) component domain.
Data are represented as mean ± SEM.

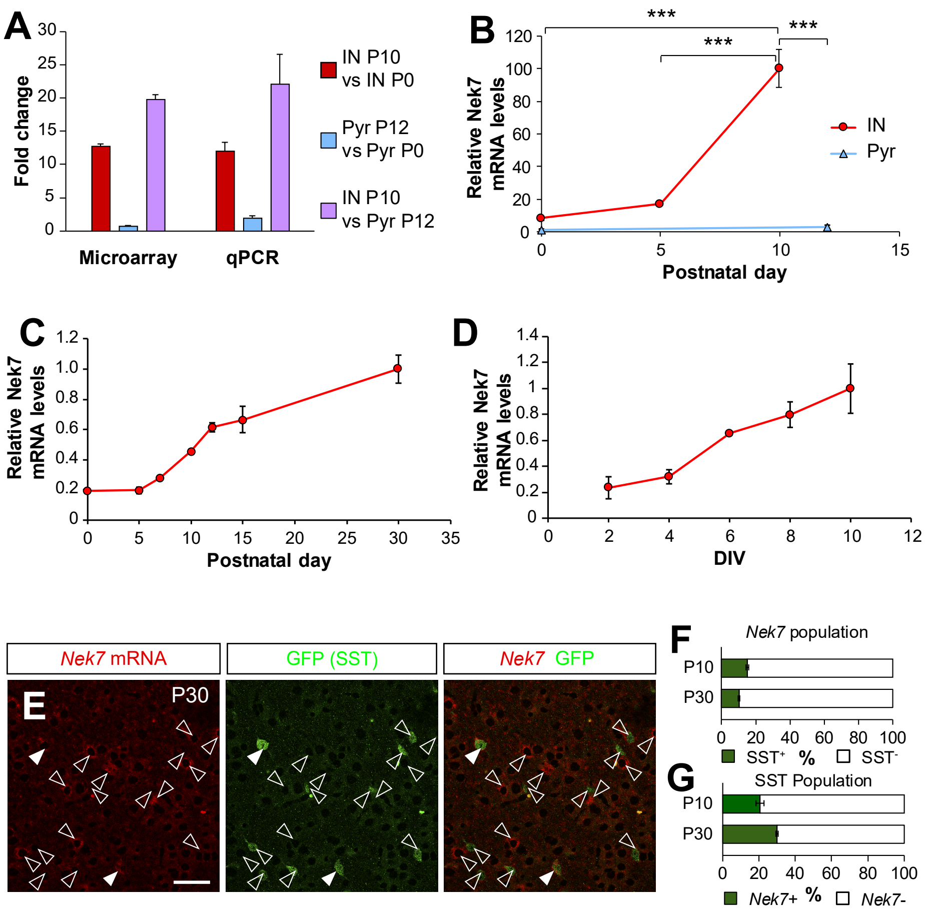

Figure S3. *Nek7* transcript expression levels along development. Related to Figure 1.
(A) Fold change values from comparisons between the different experimental conditions using Microarray and qPCR analyses.
(B) *Nek7* mRNA levels measured by qPCR in FACs sorted neurons from *Nkx2-1Cre;RCE* (IN, n = 3 mice per age) and *NexCre;RCE* (Pyr, n = 3 mice per age) relative to 18S.
(C) *Nek7* mRNA levels normalized to 18S in whole cortices at different postnatal stages (n = 4 mouse per age). All data is normalized to the average of the higher age.
(D) Same as in C for different DIV (n = 3 cultures per age).
(E) Confocal images showing in situ hybridization for *Nek7* (red) and immunohistochemistry for GFP (green) in the somatosensory cortex of P30 *SSTCre;RCE* mice. Filled arrowheads: colocalization, open arrowheads: no colocalization.
(F) Percentage of SST+ (Green) among all *Nek7*-expressing cells at P10 and P30.
(G) Percentage of *Nek7* positive cells among all SST expressing neurons at P10 and P30.
For representative pictures of P10 SST and *Nek7* staining refer to figure 1D.
One-way ANOVA, Post hoc Bonferroni, *** $p < 0.001$, Data are represented as mean ± SEM.

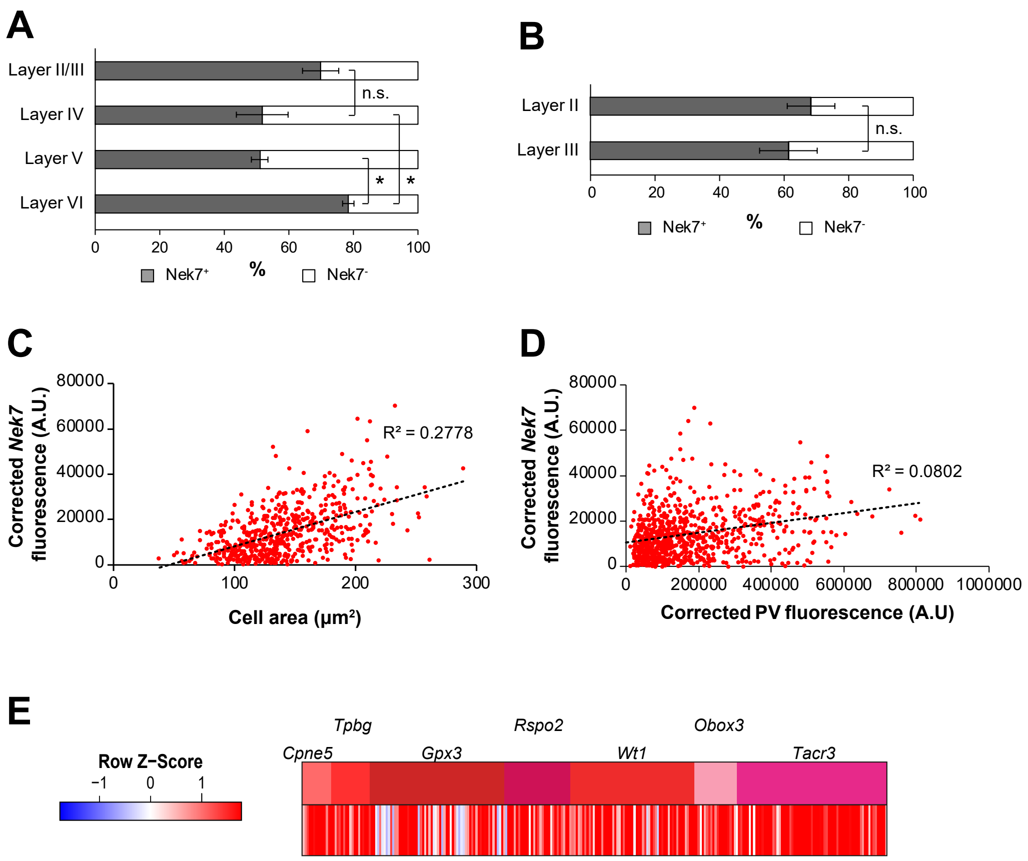

Figure S4. ***Nek7*** **seems to be expressed in most types of PV+ cells. Related to Figure 1.**
(A, B) Percentage of *Nek7* positive cells among PV expressing neurons across cortical layers in the somatosensory cortex of P30 wild-type mice. Layer II-III was divided in half to consider putative layer II and III.
(C) *Nek7 in situ* fluorescence intensity as a function of somatic area of PV+ cells.
(D) *Nek7 in situ* fluorescence as a function of PV fluorescence.
(E) Heatmap showing the expression levels of Nek7 in individual PV cells (lower row) from different genetic clusters (upper row). Row Z-score: standard deviation from the mean intensity values. (Data extracted from Tasic *et al.*, 2016).
Data are represented as mean ± SEM (A, B) or individual values for each cell (C, D).

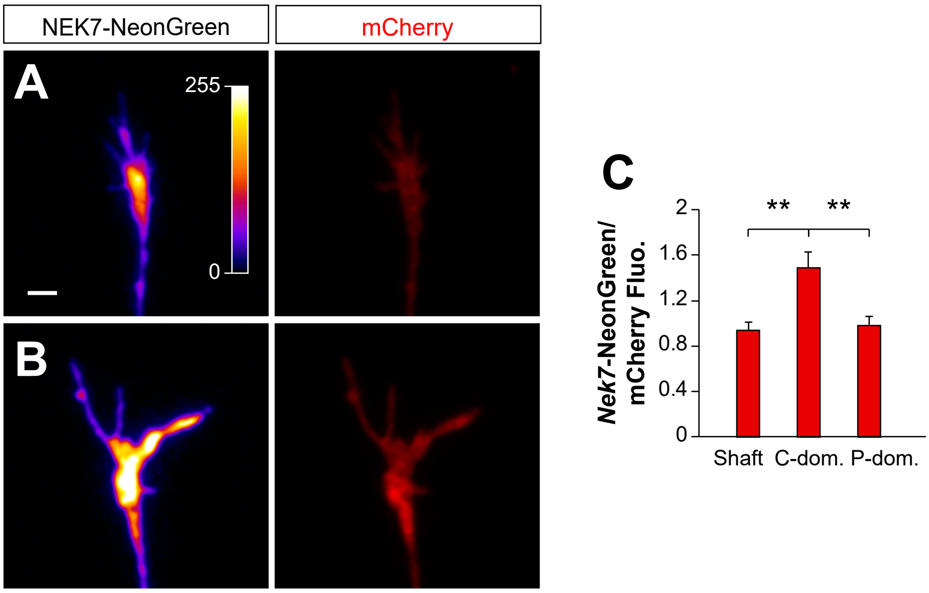

Figure S5. NEK7 is enriched in the central domain of growth cones. Related to Figure 2.
(A, B) Confocal Z-projection frames of two examples of Nkx2-1Cre growth cones co-expressing NEK7 NeonGreen (pseudocolor) and mCherry (red).
(C) NEK7 NeonGreen fluorescence intensity normalized to mCherry fluorescence in different regions of the growth cone (Shaft: axonal shaft, C-dom: central domain, P-dom: peripheral domain).
Kruskal-Wallis test, pairwise comparisons, ** $p < 0.01$. Data are represented as mean ± SEM.
Scale bar represents 2 µm.

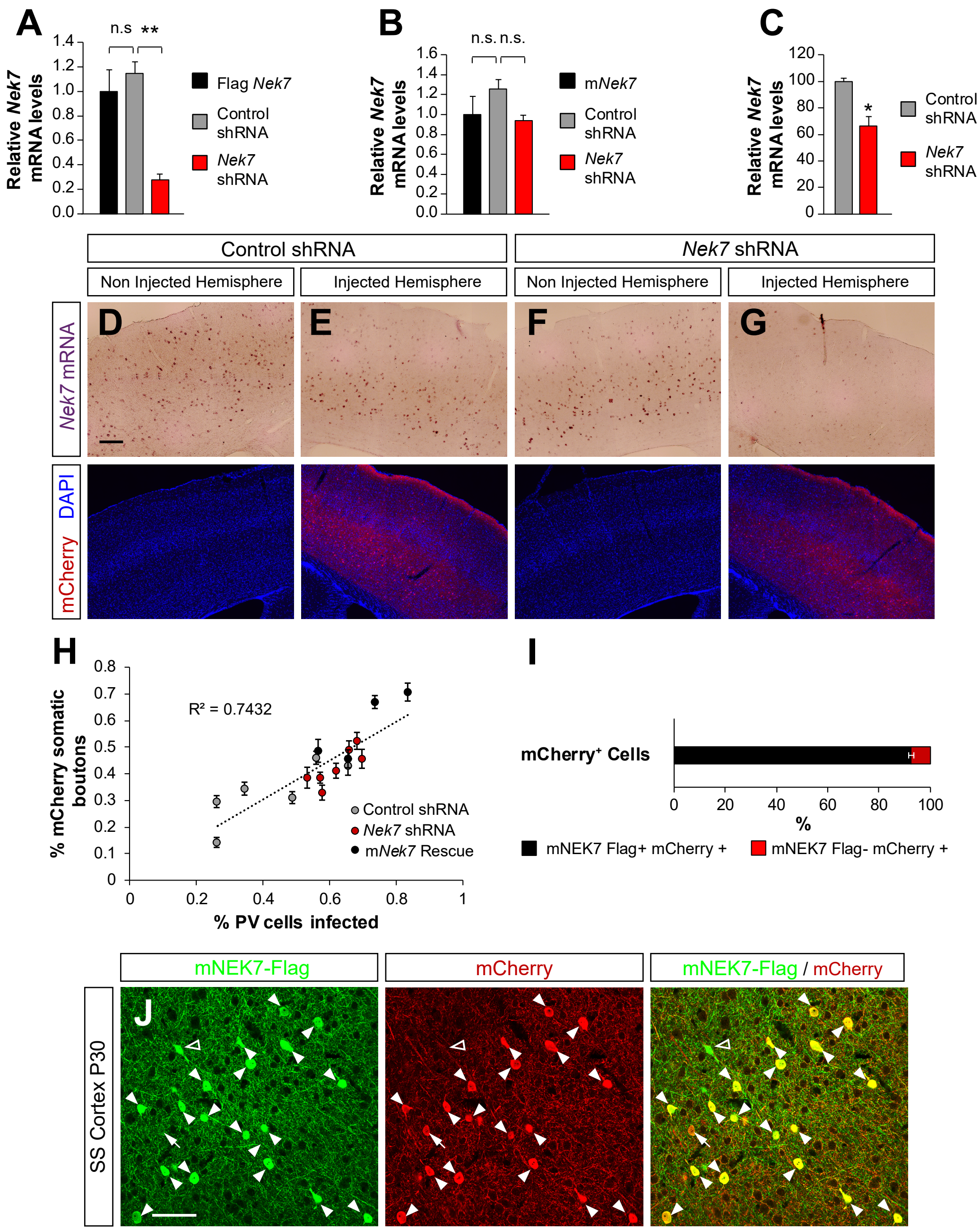

Figure S6. *Nek7* shRNA validation assays. Related to Figures 2, 3, 4, 6, 7.

(A) *Nek7* mRNA levels in HEK293 cells transfected with plasmids containing Cre, and the Cre-dependent plasmids Flag Nek7 alone (black, n = 5 cultures) or together with control shRNA (grey, n = 4 cultures) or *Nek7* shRNA (red, n = 5 cultures). Levels are relative to the non-shRNA condition (black).

(B) Same experimental conditions than (A) but using the synonymously mutated form of *Nek7* (m*Nek7*) alone (black, n = 5 cultures) or together with control shRNA (grey, n = 4 cultures) or *Nek7* shRNA (red, n = 3 cultures).

(C) *Nek7* mRNA levels in dissected cortices from P30 *Lhx6Cre* mice infected with an AAV containing control shRNA (n = 3 mice) or *Nek7* shRNA (n = 4 mice).

(D-G) Top panels, colorimetric in situ hybridization illustrating *Nek7* mRNA expression in P30 *Lhx6Cre* mice of non-injected hemispheres (D and F) and the contralateral hemispheres injected with AAV viruses expressing control shRNA (E) and *Nek7* shRNA (G). Bottom panels, contiguous coronal sections to the ones used in the top panels showing the area of infection labeled by mCherry (red). One-way ANOVA, Post hoc Bonferroni (A, B), Mann-Whitney test (C). * $p < 0.05$, ** $p < 0.01$, n.s. not significant.

(H) Percentage of mCherry+ somatic boutons (SYT2+) as a function of the percentage of PV+ cells infected in the area.

(I) Percentage of mCherry+ expressing cells that co-express m*Nek7* (Flag+) during coinfection of both AAVs.

(J) Single confocal images showing Flag+NEK7 (green) and mCherry+ (red) immunostaining in the somatosensory cortex of P30 Lhx6Cre mice coinfected with an AAV expressing *Nek7* shRNA and m*Nek7*. Colocalizing cells (filled arrowheads), mCherry- NEK7+ (open arrowheads). Data are represented as mean ± SEM.

Scale bars represent 200 μm (D-G) and 50 μm (J).

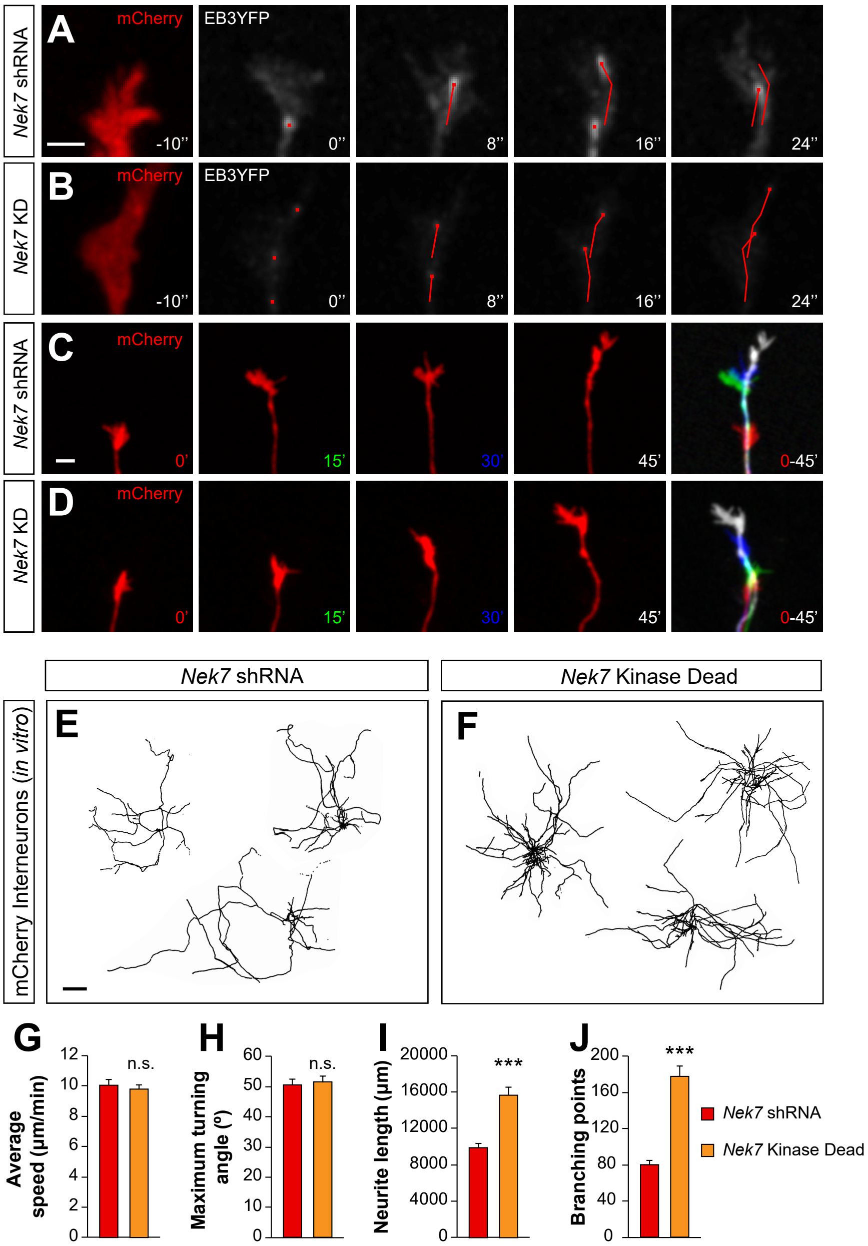

Figure S7. NEK7 kinase activity regulates different aspects of interneuron axonal development. Related to Figures 2, 3, 4.

(A, B) Confocal Z-projection frames from *Nkx2-1Cre* growth cones expressing mCherry (red, before the time-lapse) and EB3-YFP (gray, time-lapse). The path of EB3-YFP comets is tracked with a red line.

(C, D) Confocal Z-projection frames from *Nkx2-1Cre* growth cones. The last frame in the sequence shows superimposed images of the frames t = 0 min (red), t = 15 min (green), t = 30 min (blue) and t = 45 min (white).

(E, F) Confocal Z-projections of *Nkx2-1Cre* interneurons expressing mCherry. The cells were automatically reconstructed and masked in black at 12 DIV.

(G) Average speed of EB3-comets comparing *Nek7* depleted cells (A, n = 35 growth cones) and *Nek7* KD (B, n = 39 growth cones) from 3 independent cultures.

(H) Average of growth cone maximum turning angle from cells expressing *Nek7* shRNA (C, n = 59 growth cones), and *Nek7* KD (D, n = 75 growth cones) from 3 independent cultures.

(I-J) Neurite length (I) and branching points (J) comparing *Nek7* shRNA (E, n = 45 cells) and *Nek7* KD (F, n = 43 cells) transfected neurons from 3 independent cultures.

One-way ANOVA (G), Kruskal-Wallis test, pairwise comparisons (H-J). *** $p < 0.001$, n.s. not significant. Note that we used the panels and data from controls showed in Figures 2, 3 and 4 for the statistical comparisons. *Nek7* KD: *Nek7* Kinase Dead. Data are represented as mean ± SEM.

Scale bars represent 2 µm (A, B), 5 µm (C, D) and 200 µm (E, F).

SUPPLEMENTAL TABLES

Table S1

Gene symbol	Probeset	INP10 vs INP0		INP10 vs PyrP12		τ
		Q-value	Fold change	Q-value	Fold change	
Tac1	10536363	0.044	17.900	0.039	61.607	0.971
Hapln1	10406519	0.044	27.553	0.039	15.673	0.954
Nek7	10358259	0.044	12.648	0.039	19.695	0.944
Rbp4	10467319	0.044	14.438	0.039	13.506	0.935
Crhbp	10411215	0.044	10.481	0.039	18.558	0.932
Lgals1	10425161	0.044	13.226	0.039	17.109	0.931
Akr1c18	10407435	0.044	7.571	0.039	18.970	0.919
Pcp4l1	10360053	0.044	10.955	0.039	9.737	0.907
Th	10569370	0.044	8.353	0.039	8.551	0.882
Hpse	10531737	0.044	7.935	0.039	8.167	0.872
Gjc3	10534960	0.044	4.770	0.039	8.438	0.859
Sparc	10386058	0.044	4.282	0.039	7.682	0.847
Sst	10438730	0.044	2.487	0.039	45.753	0.845
Ret	10547227	0.044	6.475	0.039	6.853	0.842
Igf1	10365559	0.044	4.006	0.039	9.341	0.841
Cnp	10381154	0.044	3.692	0.039	7.468	0.837
Gpx3	10376201	0.044	4.649	0.039	6.088	0.832
Gjd2	10485979	0.044	3.607	0.039	6.528	0.823
Syt2	10350077	0.044	6.159	0.039	5.317	0.823
Slc32a1	10478124	0.044	2.378	0.039	18.729	0.821
Penk	10511363	0.044	7.371	0.039	3.847	0.815
Nrsn2	10488617	0.044	6.333	0.039	3.761	0.814
Grin2d	10563421	0.044	3.223	0.039	7.047	0.812
Moxd1	10362186	0.044	3.790	0.039	5.691	0.811
Pdyn	10487613	0.044	5.898	0.039	4.309	0.810
Mybpc1	10371627	0.044	5.144	0.039	4.897	0.803
Gpr83	10583286	0.044	5.038	0.039	4.653	0.799
Trmt2b	10606658	0.044	4.504	0.039	5.032	0.798
Zcchc12	10599187	0.044	2.612	0.039	8.489	0.798
Calb2	10581654	0.044	8.527	0.039	4.040	0.796
Nmbr	10361818	0.044	5.284	0.039	3.773	0.789
Oprd1	10516852	0.045	3.501	0.039	4.171	0.773
Pacsin2	10430997	0.044	4.949	0.039	3.109	0.769
Cnn2	10364593	0.044	3.602	0.039	4.961	0.766
Akr1b10	10537157	0.044	4.526	0.039	3.183	0.764
Adamts15	10591988	0.044	4.362	0.039	4.234	0.761
Ndrg2	10419578	0.045	2.510	0.039	7.708	0.759
Pnoc	10420853	0.044	3.341	0.039	5.230	0.758
Rassf4	10547177	0.044	3.435	0.039	5.002	0.758
Zfp804a	10473244	0.044	4.963	0.039	4.639	0.755
Lpl	10572130	0.044	16.734	0.039	2.390	0.750
Pdlim3	10571601	0.044	3.929	0.039	3.855	0.750
Mme	10492355	0.044	2.335	0.039	10.416	0.750
Cthrc1	10423836	0.044	3.551	0.039	3.857	0.747

Gpr37l1	10358023	0.045	2.745	0.039	4.659	0.746
Frmpd1	10504534	0.044	4.364	0.039	3.801	0.744
Alk	10452734	0.044	2.643	0.039	4.851	0.741
Sytl5	10598603	0.044	2.613	0.039	5.664	0.741
Filip1	10595298	0.044	4.430	0.039	2.740	0.729
Nnmt	10593219	0.044	3.796	0.039	3.562	0.724
Cplx1	10532180	0.044	4.292	0.039	2.718	0.723
Hap1	10391084	0.044	2.981	0.039	3.258	0.720
Ankrd29	10457536	0.044	3.376	0.039	3.274	0.719
Fam134b	10423333	0.044	4.010	0.039	2.596	0.719
Eya1	10353192	0.044	1.977	0.039	5.906	0.718
Vwc2	10374315	0.044	2.021	0.039	6.188	0.714
Pip5kl1	10471443	0.044	3.628	0.039	2.957	0.711
Igsf11	10435733	0.045	2.763	0.039	2.986	0.710
Ch25h	10467136	0.044	3.515	0.039	3.028	0.708
Ankrd34b	10406590	0.044	3.935	0.039	2.651	0.704
Cldn1	10438769	0.044	4.623	0.039	4.588	0.702
Egln3	10400304	0.044	2.301	0.039	6.583	0.701
Stac2	10390560	0.044	2.137	0.039	8.648	0.700
Adamts5	10440534	0.046	1.959	0.039	5.354	0.699
Btbd11	10365428	0.044	2.345	0.039	6.688	0.695
Arhgef6	10604713	0.044	2.447	0.039	4.205	0.694
Rab3b	10506883	0.044	2.805	0.039	3.261	0.693
Rcn1	10485645	0.044	3.935	0.039	2.581	0.692
Ptgs1	10471721	0.044	2.797	0.039	3.213	0.692
Gpr149	10498441	0.044	3.193	0.039	3.908	0.689
Klf3	10522051	0.044	2.136	0.039	6.698	0.685
Nacc2	10480901	0.044	2.290	0.039	3.502	0.685
Dmrt2	10462231	0.045	3.200	0.039	3.910	0.684
Pvt1	10424404	0.044	3.920	0.039	2.164	0.684
Ifit2	10462613	0.050	2.230	0.039	3.915	0.682
Thbs2	10447951	0.044	4.505	0.039	2.048	0.681
Ppif	10413222	0.044	2.884	0.039	3.283	0.681
Lypd6	10472034	0.044	2.586	0.039	3.589	0.680
Zim1	10559790	0.044	2.872	0.039	3.548	0.680
Flt3	10535780	0.044	3.764	0.039	2.261	0.675
Sfrp2	10492798	0.044	2.382	0.039	7.359	0.670
Grm1	10367830	0.044	2.394	0.039	3.162	0.666
Nefh	10383920	0.044	3.776	0.039	2.069	0.666
Id4	10404975	0.044	2.137	0.039	5.512	0.666
Kcnab1	10492402	0.044	8.561	0.039	2.133	0.665
Slc27a2	10475653	0.044	2.916	0.039	2.907	0.660
Aldh5a1	10408335	0.044	2.825	0.039	2.476	0.657
Doc2b	10388465	0.044	4.076	0.039	3.027	0.655
Alcam	10439895	0.044	3.266	0.039	2.335	0.651
Ndst3	10501924	0.044	5.214	0.039	2.368	0.645
Zfp385a	10433104	0.044	2.313	0.039	2.702	0.644
Myo5b	10456653	0.044	3.914	0.039	2.844	0.643

Phactr2	10367945	0.044	2.099	0.039	3.154	0.643
Ppapdc1a	10558049	0.044	2.180	0.039	3.363	0.642
Pgm2	10506188	0.044	2.049	0.039	2.787	0.640
Alox8	10387257	0.044	3.051	0.039	2.397	0.638
Rragb	10602692	0.044	3.048	0.039	2.288	0.636
Optn	10479833	0.044	3.082	0.039	2.670	0.634
Btn2a2	10408185	0.044	2.898	0.039	2.533	0.631
Adamts6	10406982	0.044	2.278	0.039	3.575	0.630
Drd2	10585169	0.044	2.433	0.039	3.023	0.628
Sema3c	10519886	0.045	3.229	0.039	2.020	0.619
Chrna2	10416071	0.045	2.605	0.039	2.591	0.619
Plch1	10498448	0.044	2.573	0.039	2.034	0.617
Crh	10497417	0.045	2.852	0.039	2.282	0.616
Pygb	10476969	0.044	3.053	0.039	2.589	0.616
Htr1d	10509238	0.044	2.567	0.039	2.414	0.608
Tmhs	10443383	0.044	2.706	0.039	2.633	0.606
Prdx3	10468869	0.046	2.663	0.039	2.242	0.604
Fam101b	10388488	0.044	2.716	0.039	2.010	0.603
Gys1	10552945	0.045	2.475	0.039	2.566	0.598
Acaa2	10456699	0.044	2.991	0.039	3.493	0.598
Kcnh2	10528548	0.044	2.146	0.039	2.885	0.591
Wif1	10366653	0.044	2.383	0.039	2.409	0.591
Dgkk	10598251	0.044	2.690	0.039	2.102	0.585
Tac2	10367024	0.048	2.440	0.039	2.167	0.582
Uprt	10601328	0.045	2.548	0.039	2.196	0.581
Acadl	10355246	0.044	2.529	0.039	2.074	0.579
Lypd6b	10472022	0.044	2.051	0.039	2.565	0.575
Shisa9	10433618	0.044	2.862	0.039	2.002	0.572
Fam195a	10449142	0.044	2.441	0.039	2.006	0.562
Prss23	10565456	0.044	2.218	0.039	2.168	0.561
Tacr1	10539244	0.044	2.092	0.039	2.440	0.558
C330016O10Rik	10385709	0.044	2.013	0.039	2.702	0.555
Rerg	10548899	0.044	2.217	0.039	2.989	0.554
Arhgap18	10362294	0.044	2.450	0.039	2.242	0.554
Baiap3	10448878	0.044	2.056	0.039	2.354	0.552
Fam150b	10395136	0.045	1.980	0.039	2.095	0.528
Fam43a	10434932	0.045	2.030	0.039	2.065	0.519
Hpcal1	10394778	0.045	2.052	0.039	2.531	0.511
Timp3	10365482	0.044	2.732	0.039	3.302	0.509
Luzp2	10553537	0.044	2.114	0.039	3.882	0.486
Rgag1	10602196	0.044	2.185	0.039	2.747	0.107

Table S1. Differentially and specifically upregulated genes during GABAergic wiring. Related to Figure 1. List of 133 genes upregulated during GABAergic wiring (IN P0 versus IN P10) and specifically expressed in interneurons (IN P10 versus Pyr P12). Pairwise comparisons were done using Significance Analysis of Microarrays (SAM) and genes with a false discovery rate (FDR) lower than 0.05 and a fold change higher than 2 were selected. The table reports: gene symbol, microarray probeset number, q-value (adjusted p-value), fold change for both comparisons, and specificity ratio for IN P10 (τ). Genes are sorted according to τ.

SUPPLEMENTAL EXPERIMENTAL PROCEDURES

Mice

Mice were maintained in a C57BL/6J background. *Nkx2-1Cre* (Sussel et al., 1999) and *NexCre* (Goebbels et al., 2006) mice were crossed with the Cre dependent reporter line R26R CAG-boosted EGFP (*RCE:LoxP*) (Miyoshi et al., 2010) for the genomic screening to label interneurons and pyramidal cells respectively. *Nek7 in vivo* loss of function experiments were carried out in *Lhx6Cre* mice (Fogarty et al., 2007), which were always kept in heterozygosis. Heterozygous *Nkx2-1Cre* mice were used for the primary cortical cultures. Homozygous Sst-IRES-Cre mice (Taniguchi et al., 2011) were crossed with *RCE:loxP* for colocalization with *Nek7* transcript.

FACS, microarrays and qPCR

For the FACS experiments, mice were anesthetized with sodium pentobarbital and the somatosensory cortex, in *NexCre;RCE:LoxP*, and whole neocortex, in *Nkx2-1Cre;RCE:LoxP* (to increase cell yield), were dissociated as described before (Catapano et al., 2001). Two to six mice were collected per run until having a minimum of 10^5 cells per microarray. The total number of cells per condition was a follows: for pyramidal cells, *NexCre;RCE,* $1.6 \cdot 10^6$ and $5.7 \cdot 10^5$ for P0 and P12, respectively; for interneurons, *Nkx2-1Cre;RCE*, $1.3 \cdot 10^6$ and $6.9 \cdot 10^5$ for P0 and P10, respectively. For RNA purity validation, microarray hybridization and bioinformatic analysis, RNA samples were sent to the genomics facility of "Centro de Investigación del Cancer" at University of Salamanca. Three replicates were done for all the conditions assayed. Briefly, 100-300 ng of total RNA were amplified using the WT Expression Kit (Ambion), labelled using the GeneChip WT Terminal Labeling Kit (Affymetrix) and hybridized to Mouse Gene 1.0 ST Array (Affymetrix).

Given the expression of *Nkx2-1* in a population of oligodendrocytes (Nery et al., 2001), we filtered our final list to remove genes specifically expressed in oligodendrocytes during early postnatal development (Cahoy et al., 2008). The final 133 genes were ranked according to the specificity ratio (τ) in IN P10. The 20 first genes were selected and scored according to both their specificity ratio and the total levels in IN P10. τ was calculated as described before (Kryuchkova-Mostacci and Robinson-Rechavi, 2017). Finally, gene lists were analyzed for possible known roles in the literature using Gene Ontology (Ashburner et al., 2000). To do this the R-package ClusterProfiler was used in R (Yu et al., 2012). A q-value cut off of 0.05 was applied in all the analysis carried out and the 15 most significant categories are shown.

RNA samples used in the quantitative PCR (qPCR) experiments were retronstranscribed using SuperScript IV according to manufacturer instructions (ThermoFisher Scientific). qPCR was carried out in triplicates using SYBR Green PCR Master Mix (Roche) on a LightCycler 96 Instrument (Roche). Normalized mRNA levels of *Nek7* relatively to those of 18S or mCherry were calculated using the number of cycles to reach a threshold signal (Cq) according to the $2^{-\Delta\Delta Cq}$ equation. qPCR primer sequences were as follows: *Nek7* (5'-AGCCACAGAAGGCATTACGG-3', 5'-CTACCGGCACTCCATCCAAG-3'), 18S (5'- GTAACCCGTTGAACCCCATTCGT-3', 5'-GTGTGTACAAAGGGCAGGGACTTAA-3') and mCherry (5'-CATCCTGTCCCCTCAGTTCATG-3', 5'- CATCCTGTCCCCTCAGTTCATG-3').

DNA constructs, viral production and viral injections

The ssDNA oligonucleotides to generate the shRNAs were: *Nek7* shRNA (5'-CTAGGAGAGAACCGTTTGGAAATACCCTGACCCAGTATTTCCAAACGGTTCTCTCTTTTTG-3' and 5'-AATTCAAAAAGAGAGAACCGTTTGGAAATACTGGGTCAGGGTATTTCCAAACGGTTCTCTC-3'), gfp shRNA (5'-CTAGGCCACAACGTCTATATCATGGCCTGACCCACCATGATATAGACGTTGTGGCTTTTTG-3' and 5'-AATTCAAAAAGCCACAACGTCTATATCATGGTGGGTCAGGCCATGATATAGACGTTGTGGC-3') and *lacZ* shRNA (5'-CTAGAAATCGCTGATTTGTGTAGTCCCTGACCCAGACTACACAAATCAGCGATTTTTTTTG-3' and 5'-AATTCAAAAAAAATCGCTGATTTGTGTAGTCTGGGTCAGGGACTACACAAATCAGCGATT

T-3').

NeonGreen was PCR amplified from a plasmid containing the gene (Allele biotechnology, Shaner et al., 2013), a Flag tag was added and it was subcloned in the C-terminus of m*Nek7* using *AscI* and *Sph1*. The kinase dead mutation was generated through targeted mutagenesis of m*Nek7* (K63M/K64M, (O'Regan and Fry, 2009).

We used the described constructs to generate AAVs (serotype rep2/cap8) as described previously (Favuzzi et al., 2017). Briefly, 80% confluent HEK293FT cells (ThermoFisher Scientific) were transfected with *pDIO-shRNA-mCherry* or m*Nek7* and AAV8 capside plasmid (pDP8.ape vector, PlasmidFactory) with polyethylenimine (PEI, SigmaAldrich) at a DNA:PEI ratio of 1:4. Viruses were harvested after three days, purified by ultracentrifugation in iodixanol gradients and filtered-concentrated using Amicon Ultra Centrifugal Filters 100 kDa (Millipore). Only AAV batches with a titer higher than 1 x 10^{12} viral genome copies/mL were used for *in vivo* experiments. For morphological reconstructions, we obtained isolated cells by injecting 1 mL of AAVs diluted 1:30, in PBS with FastGreen 0.5%, into the telencephalic lateral ventricle of E15.5 *Lhx6Cre* embryos. Pregnant females were deeply anesthetized with isoflurane (5% induction, 2% maintenance), the abdominal cavity was cut opened and the uterus exposed making the embryos accessible. After injection, the uterus was placed back in the abdominal cavity, the incision was sutured and buprenorphine 0.1 mg/Kg was used for analgesia. For the analysis of PV+ wiring in pyramidal cells, we increased the yield of infection by performing postnatal AAV stereotaxic injections in P3-P4 *Lhx6Cre mice*. After making a small incision in the skull (2.2 mm lateral and 2.6 mm anterior to interaural midpoint), two injections of 250 nL each containing these same viruses diluted 1:1 were infused at 50 nL/min using a Nanoinjector2010 (WPI).

Primary cultures and transfection

Cells were resuspended in supplemented Neurobasal A medium (Invitrogen) with 500 mM Glutamax-L, 2% v/v B-27 and 0.01 U/mL penicillin/streptomycin; and plated at a density of 100.000 cells/cm^2 on glass-bottom plates (MatTek corporation) previously coated with 0.5 mg/mL Poly-L-lysine (Sigma-Aldrich) overnight at 37ºC. Cultures at day *in vitro* 4 (4 DIV) were transfected with *Nek7* shRNA or *lacZ* shRNA using Lipofectamine 2000 (ThermoFisher Scientific) during 3 hours at a final total concentration of 0.5 µg/mL of DNA on the plate according to manufacturer's instructions. To assay microtubule dynamics, plasmids expressing EB3-YFP were co-transfected with the shRNA plasmids (ratio 1:3) using the same culture conditions as described above.

Rescue experiments were carried out by co-transfection of Cre-dependent *Nek7* shRNA together with m*Nek7* or *Nek7* KD at a ratio 1:1. To assay the effect of Nek7 in pyramidal cells, Cre-dependent constructs expressing m*Nek7* or an empty vector together with mCherry were co-transfected in *NexCre* primary cultures (ratio 1:1). Finally, NEK7 dynamics was visualized co-transfecting Cre-dependent plasmids reporting mCherry and *Nek7* NeonGreen (ratio 1:1).

Immunohistochemistry and immunocytochemistry

P21 or P30 mice were anesthetized with sodium pentobarbital and perfused transcardially with PBS followed by 4% paraformaldehyde (PFA) in PBS. Coronal slices of 40 µm (or 100 µm for the morphological reconstructions) were cut in a sliding microtome (Leica) for immunohistochemistry. Free-floating brain sections were blocked with 5% BSA, 0.3% Triton and 10% normal donkey serum during 2 hours at room temperature and incubated with primary antibodies at 4ºC in 1% BSA, 0.3% triton and 5% normal donkey serum overnight (40 µm sections) or 2 days at room temperature (100 µm). The following day, sections were rinsed in PBS and incubated in the appropriated secondary antibodies for 2 hours (40 µm sections) or 4 hours (100 µm) at room temperature in the same solution as the primary antibodies. Finally, the slices were rinsed in PBS, incubated with DAPI and mounted in Mowiol/DABCO.

Fixed primary cultures were rinsed and permeabilized with 0.5% triton during 15 min. The plates were blocked in blocking solution (2% BSA and 2% normal donkey serum) during 1 hour and incubated with primary antibodies diluted in the same solution overnight. The following day they were rinsed in PBS, incubated with secondary antibodies for 1 hour, stained with DAPI and mounted with Mowiol/DABCO.

The following primary antibodies were used: rabbit anti-DsRed (1:500, Clontech 632496), mouse anti-Flag (1:500, Sigma F1804), goat anti-mCherry (1:500-1:1000, antibodies-online ABIN1440057), mouse anti-NeuN (1:500, Millipore MAB377), rabbit anti-PV (1:5000, Swant PV-25), chicken anti-PV (1:500, SySy 195 006), mouse anti-SYT2 (1:500, ZFIN ZDB-ATB-081002-25), chicken anti-GFP (Aves labs GFP-1020) and rat anti-SST (1:200, Chemicon MAB354). Secondary antibodies and Alexa-conjugated streptavidin were purchased from Molecular Probes (#A-21206, #A-31572, #A-21202, #A-31571, #A-21241, #A-21121, #A-21432, #A-21208), Jackson (#711-165-152, #016-470-084, #703-225-155) and Vector (#BA-9500).

Image acquisition and analysis

The number of mCherry+ SYT2+ boutons depends on the infection efficiency. Indeed, the proportion of SYT2+ mCherry+ boutons increased linearly with the number of PV+ infected (Fig S9A). For this reason, we normalized bouton percentages to the number of infected PV+ cells in the area (200 μm from the quantified pyramidal cell) (Fino et al., 2013).

To measure PV and NEK7 intensity levels in these *in situ* colocalizations, 12-bit images were acquired and fluorescence levels were measured with ImageJ. The Corrected fluorescence was calculated with the following formula:

Corrected fluo. = Integrated intensity- (Cell Area · Background mean fluo.)

NEK7 NeonGreen intensity levels *in vitro* were acquired in 8-bit and normalized with mCherry to correct for growth cone volume as described before (Myers et al., 2012).

For EB3 comets, Imaris spots were manually drawn on EB3-YFP comets in each frame and their tracks automatically quantified along time. For growth cone dynamics analysis, Imaris filaments were semi-automatically drawn in each frame and their tracks followed and quantified automatically along time. The turning angle was quantified as the angle formed between the growth cone center of mass and the underlying axon shaft. Neuronal morphology was automatically reconstructed by generating, first, an Imaris surface comprising the whole cell morphology, then producing a new masked channel containing exclusively the signal within this surface and, finally, automatically creating a filament with the signal coming from the masked channel.

For synaptic bouton quantification, Spots, putatively labeling PV synaptic terminals reported with SYT2 staining, were identified in the SYT2 channel (average diameter 0.5 μm) and the Imaris XTension "Split spots into surface" was used to quantify the synaptic boutons (spots) within the neurites. For the analysis of somatic SYT2 boutons onto pyramidal cells, we used the mCherry surface, SYT2 boutons and a surface from NeuN channel. First the XTension "Spots close to surface" was used to filter the spots at 0.25 μm from the NeuN surfaces, *i.e.* the somatic boutons. Subsequently, somatic boutons coming from infected cells were identified using "Split spots into surface".

SUPPLEMENTAL REFERENCES

Ashburner, M., Ball, C.A., Blake, J.A., Botstein, D., Butler, H., Cherry, J.M., Davis, A.P., Dolinski, K., Dwight, S.S., Eppig, J.T., et al. (2000). Gene ontology: tool for the unification of biology. The Gene Ontology Consortium. Nat. Genet. *25*, 25–29.

Cahoy, J.D., Emery, B., Kaushal, A., Foo, L.C., Zamanian, J.L., Christopherson, K.S., Xing, Y., Lubischer, J.L., Krieg, P.A., Krupenko, S.A., et al. (2008). A transcriptome database for astrocytes, neurons, and oligodendrocytes: a new resource for understanding brain development and function. J. Neurosci. 28, 264–278.

Catapano, L.A., Arnold, M.W., Perez, F.A., and Macklis, J.D. (2001). Specific neurotrophic factors support the survival of cortical projection neurons at distinct stages of development. J. Neurosci. *21*, 8863–8872.

Fino, E., Packer, A.M., and Yuste, R. (2013). The logic of inhibitory connectivity in the neocortex. Neuroscientist *19*, 228–237.

Fogarty, M., Grist, M., Gelman, D., Marín, O., Pachnis, V., and Kessaris, N. (2007). Spatial genetic patterning of the embryonic neuroepithelium generates GABAergic interneuron diversity in the adult cortex. J. Neurosci. 27, 10935–10946.

Goebbels, S., Bormuth, I., Bode, U., Hermanson, O., Schwab, M.H., and Nave, K.-A. (2006). Genetic targeting of principal neurons in neocortex and hippocampus of NEX-Cre mice. Genesis 44, 611–621.

Miyoshi, G., Hjerling-Leffler, J., Karayannis, T., Sousa, V.H., Butt, S.J.B., Battiste, J., Johnson, J.E., Machold, R.P., and Fishell, G. (2010). Genetic fate mapping reveals that the caudal ganglionic eminence produces a large and diverse population of superficial cortical interneurons. J. Neurosci. 30, 1582–1594.

Myers, J.P., Robles, E., Ducharme-Smith, A., and Gomez, T.M. (2012). Focal adhesion kinase modulates Cdc42 activity downstream of positive and negative axon guidance cues. J. Cell Sci. *125*, 2918–2929.

Nery, S., Wichterle, H., and Fishell, G. (2001). Sonic hedgehog contributes to oligodendrocyte specification in the mammalian forebrain. Development 128, 527–540.

Shaner, N.C., Lambert, G.G., Chammas, A., Ni, Y., Cranfill, P.J., Baird, M.A., Sell, B.R., Allen, J.R., Day, R.N., Israelsson, M., et al. (2013). A bright monomeric green fluorescent protein derived from Branchiostoma lanceolatum. Nat. Methods *10*, 407–409.

Sussel, L., Marin, O., Kimura, S., and Rubenstein, J.L. (1999). Loss of Nkx2.1 homeobox gene function results in a ventral to dorsal molecular respecification within the basal telencephalon: evidence for a transformation of the pallidum into the striatum. Development 126, 3359–3370.

Taniguchi, H., He, M., Wu, P., Kim, S., Paik, R., Sugino, K., Kvitsiani, D., Fu, Y., Lu, J., Lin, Y., et al. (2011). A resource of Cre driver lines for genetic targeting of GABAergic neurons in cerebral cortex. Neuron 71, 995–1013.

Yu, G., Wang, L.-G., Han, Y., and He, Q.-Y. (2012). clusterProfiler: an R package for comparing biological themes among gene clusters. OMICS *16*, 284–287.
